# Developing a User-Friendly Code for the Fast Estimation of Well-Behaved Real-Space Partial Charges

**DOI:** 10.1021/acs.jcim.3c00597

**Published:** 2023-06-20

**Authors:** Miguel Gallegos, Ángel Martín Pendás

**Affiliations:** Departamento Química Física y Analítica, Universidad de Oviedo, 33006 Oviedo, Spain

## Abstract

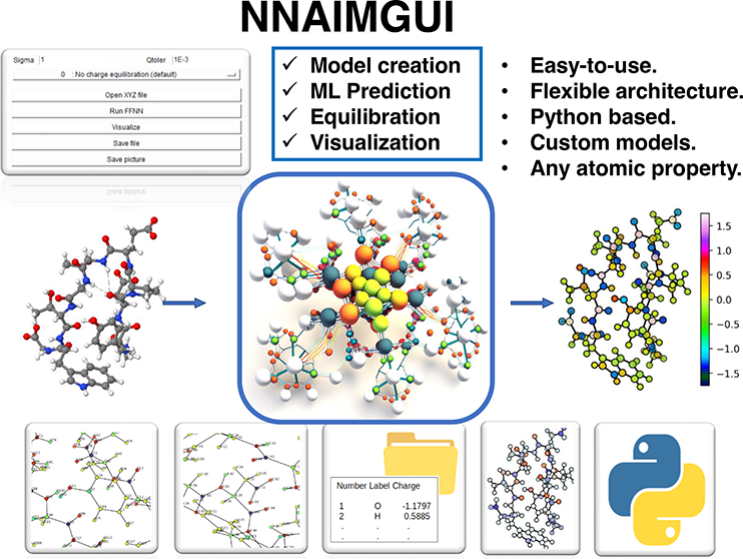

The Quantum Theory of Atoms in Molecules (QTAIM) provides an intuitive, yet physically sound, strategy to determine the partial charges of any chemical system relying on the topology induced by the electron density ρ(**r**) . In a previous work [*J. Chem. Phys.***2022**, *156*, 014112], we introduced a machine learning (ML) model for the computation of QTAIM charges of C, H, O, and N atoms at a fraction of the conventional computational cost. Unfortunately, the independent nature of the atomistic predictions implies that the raw atomic charges may not necessarily reconstruct the exact molecular charge, limiting the applicability of the latter in the chemistry realm. Trying to solve such an inconvenience, we introduce NNAIMGUI, a user-friendly code which combines the inferring abilities of ML with an equilibration strategy to afford adequately behaved partial charges. The performance of this approach is put to the test in a variety of scenarios including interpolation and extrapolation regimes (e.g chemical reactions) as well as large systems. The results of this work prove that the equilibrated charges retain the chemically accurate behavior reproduced by the ML models. Furthermore, NNAIMGUI is a fully flexible architecture allowing users to train and use tailor-made models targeted at any atomic property of choice. In this way, the GUI-interfaced code, equipped with visualization utilities, makes the computation of real-space atomic properties much more appealing and intuitive, paving the way toward the extension of QTAIM related descriptors beyond the theoretical chemistry community.

## Introduction

Most of the classical, and even some of the modern, chemical narrative used to understand and predict the properties and reactivities of a wide variety of compounds has relied, and still does, on the electron density ρ(**r**). Moreover, chemical intuition, and especially within the organic chemistry realm, is inevitably grounded in ρ-related concepts such as electron localization and delocalization. Indeed, the latter terms have been very appealingly employed to rationalize multiple chemical phenomena,^1,2^ including the reactivity,^3^ stability,^4^ and chemical properties^5^ (e.g., basicity vs nucleophilicity) exhibited by a large collection of scaffolds and supramolecular systems.^6,7^ Such a trend, triggered by the birth and development of theoretical and computational chemistry, has crystallized in numerous quantum-chemical (QM) descriptors aimed at measuring the magnitude of such an electron rearrangement. Within these, atomic charges, condensing the extent of the local accumulation or depletion of ρ(**r**) in a molecule, have spread widely across the chemistry community, given their simplicity, low computational cost, and intuitive analysis. For instance, they are known to play a crucial role in current state-of-the-art analyses such as those employed in the computational modeling of complex supramolecular processes.^8−10^

Given the importance that partial charges play in chemistry, numerous methodologies and approximations have been derived for their computation. In this context, the Quantum Theory of Atoms in Molecules (QTAIM),^11^ formulated by Richard Bader, offers one of the most rigorous and intuitive ways to estimate the local electron count of an atom. To do so, the topology of ρ(**r**) is used to decompose the *R*^3^ space into a collection of attraction domains from which atomic and pairwise properties can be obtained upon integration of the corresponding quantum mechanical operators. Unlike fitting or semiempirical approaches, such as the commonly employed Restrained Electrostatic Potential (RESP) model,^12^ QTAIM charges are solely derived from first principles, resulting in more robust and reliable values. However, just as it happens with other descriptors grounded in quantum chemical topology (QCT), the fairly high computational cost of QTAIM calculations has limited their applicability to small systems. Aiming at ameliorating such a problem, we recently presented NNAIMQ,^13^ which, according to our knowledge, is one of the first^14^ neural network based machine learning (ML) models designed for the fast and accurate computation of Bader quantities. The latter comprises four atomistic feed-forward neural networks (FFNN) trained to compute QTAIM atomic charges of C, H, O, and N atoms embedded in neutral gas-phase molecules at the M06-2X/def2-TZVP level of theory. This approach has been proven to exhibit reasonable prediction accuracies for never-seen data, with mean absolute errors between 0.007 and 0.015 electrons on average, while being several orders of magnitude faster than standard quantum-chemical calculations. However, the use of independent atomistic models implies that the expected electroneutrality of the global systems is not ensured by construction, something which is furthermore accentuated by the, usually, additive nature of the atomic errors. Such an inconvenience, particularly problematic in extrapolation regimes, hinders the application of the ML models in certain scenarios where quantitatively correct electron counts are required. For instance, accurate and well-behaved electron distributions are an absolute must when it comes to estimating the electrostatic component to the total interaction energy, where small errors in the former can easily have detrimental effects on the reliability of the latter.^15−17^

To tackle the aforementioned problem, and thus increase the applicability of previous models, in this work, we implement different charge equilibration schemes which redistribute the excess molecular charge to recover the desirable neutral character. Moreover, and trying to spread the use of ML models outside the computational chemistry community, we have designed a user-friendly graphical user interface (GUI) which makes the computation of Bader atomic properties much easier and appealing to the average user. This Python based code, named NNAIMGUI, bears multiple features to facilitate the calculation and analysis of the results including: a built-in functionality to load the starting geometries, a chemical featurization module, multiple charge equilibration schemes, and a 3D-visualization tool, among others. Moreover, its flexible architecture allows users to easily train and apply tailor-made FFNN models, enabling the prediction of atomic properties within any chemical space of choice. Besides presenting the NNAIMGUI code, the reliability and performance of the implemented electron redistribution approaches is put to the test in multiple scenarios going from standard equilibrium geometries to chemical reactions and to even large size systems. The manuscript is organized as follows: first, a brief overview of the theoretical foundations behind the NNAIMQ approach and the charge equilibration schemes is presented. Then, the algorithmic details of the NNAIMGUI code are introduced, and the performance of the latter is finally put to the test, paying special attention to the accuracy of the resultant atomic charges and the chemical insights that can be distilled from their analysis. The final section gathers the conclusions which can be drawn from this work.

## Theoretical Background

As first introduced by Bader,^11^ the Quantum Theory of Atoms in Molecules (QTAIM) describes a chemical system relying entirely on the topological features induced by the ρ(**r**) scalar field, resulting in a partition of *R*^3^ into a collection of well-defined domains or basins (Ω) separated by zero-flux surfaces of the ∇ρ(**r**) field. Given the nonoverlapping nature of the QTAIM basins, the expectation value of any one-electron operator Θ can be exactly reconstructed in terms of local basin expectation values, Θ_Ω_. Hence, under the QTAIM approach, the local electron population of an atom (A) can be obtained from the integration of ρ(**r**) within the attraction basin of A (Ω_A_), and thus, the atomic charge (*q*_A_) becomes readily available as
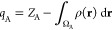
1where *Z*_A_ is the atomic number of atom A. And, similarly, the molecular charge (*Q*) of an *N*-atom system is obtained from the summation of all the local values:
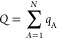
2As previously mentioned, the ML predicted atomic charges are, unfortunately, not free of errors. The latter can easily add up to each other, resulting in a predicted molecular charge different from the exact (quantum-chemically derived) one. It is thus convenient to measure this excess or deficiency of electrons for a given system as
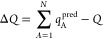
3where *q*_A_^pred^ is the predicted atomic charge for atom A. It should be noticed that, for neutral molecules (*Q* = 0), the previous expression is simplified to the first term (Δ*Q* = ∑_*A*=1_^*N*^*q*_A_^pred^).

**Figure 1:**
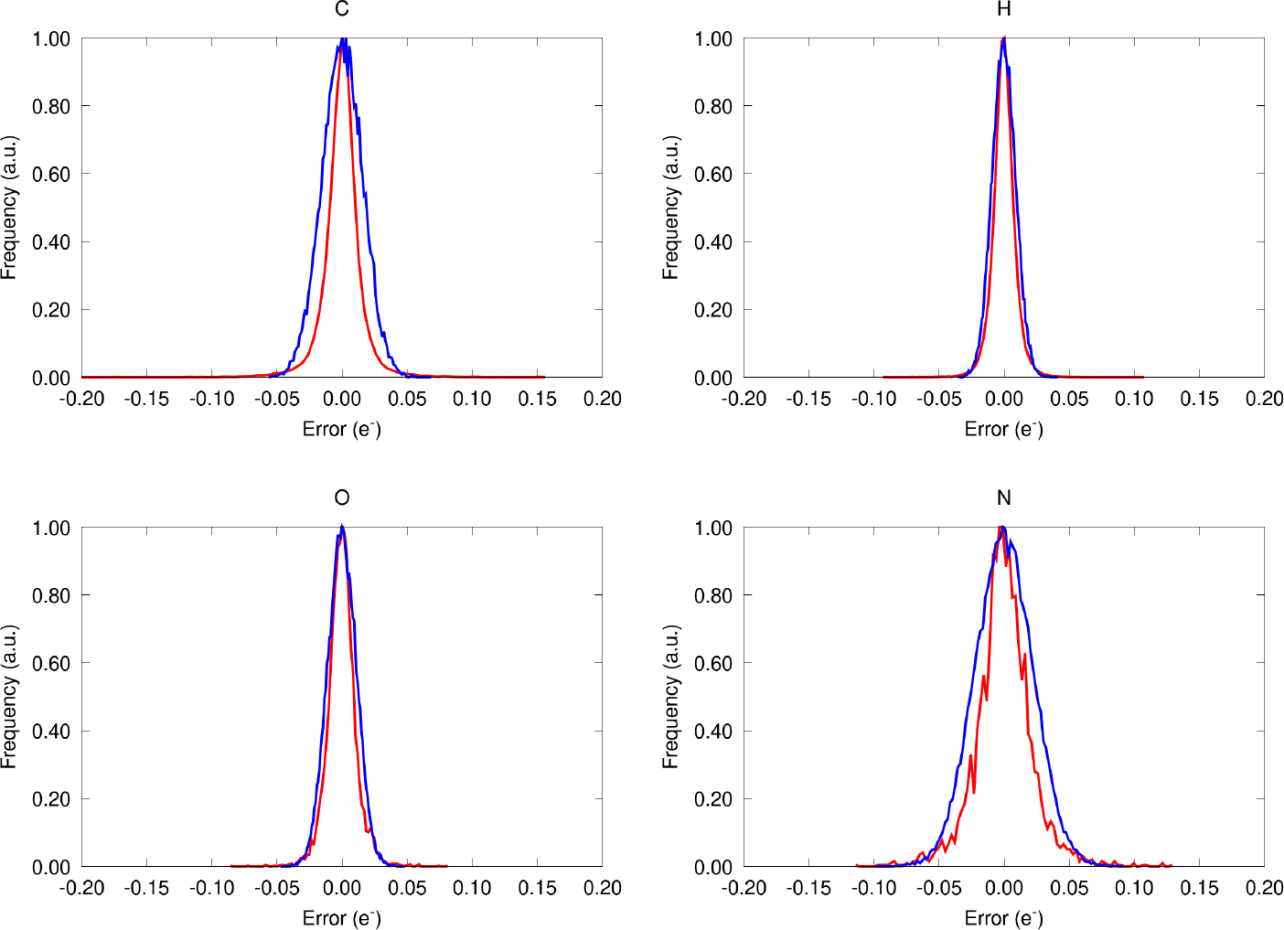
Error distribution of the NNAIMQ predictions evaluated with the external validation (testing) data set. The real and fitted distributions are shown in red and blue, respectively.

To alleviate this offset in the reconstruction of the neutral molecular character, electron redistribution techniques (here referred to as charge equilibration) can be applied. For the sake of clarity, it may be worth pointing out that the equilibration schemes used in this context are rescaling techniques which try to ameliorate the faults of ML models and should not be mistaken for the quantum-chemical approaches used sometimes to compute atomic charges in the first place, something which is particularly common within standard molecular dynamics simulations and related fields^18−22^ and which has been even merged with modern ML models.^23^ In the context of ML, different electron redistribution algorithms have been developed over the years.^24−26^ Under most common approaches, a subtle correction is applied to every atomic charge (η) such that the final Δ*Q* vanishes. For a given atom A, the correction may take the following general functional form:
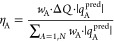
4where *w*_A_ represents the weight given to a particular atom A. After this, the corrected atomic charges can be readily obtained by subtracting the η correction to the predicted atomic charge:
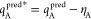
5From the aforementioned expressions, it becomes clear that a plethora of different algorithms can be employed to assign the atomic weights (*w*), leading, hence, to numerous equilibration schemes. Under the simplest approximation, for instance, the weights can be homogeneously distributed among all of the constituting particles, such that for an *N*-atom system, *w* is set to 1/*N* for all atoms. Despite its simplicity, this trivial weighting scheme has been successfully applied in the literature^24,25^ to correct ML predicted partial charges. Additionally, some other more sophisticated and robust methods have also been proposed in recent years. For instance, Rai and Bakken^26^ employed the uncertainty of the predictions to refine the atomic charges coming from Random Forest Regressions models, something which has also been successfully used by Bleiziffer et al.^27^ to obtain robust density derived electrostatic and chemical DDEC charges^28^ as well as other relevant properties of lead-like molecules. Unfortunately, this type of approach cannot be straightforwardly applied to independently derived atomic charges, owing to the fact that each prediction arises from a single ML model. Nevertheless, an alternative, yet analogous, strategy can be derived from averaging the errors committed by each atomistic NN. Although the partial charge of each atom is derived by a single model, and thus we cannot assign a conventional standard deviation to a prediction, it is still possible to use the average error expected in this prediction as a measure of the uncertainty. For such a purpose, we decided to employ the error metrics exhibited by the NN models when evaluated within the NNAIMQ testing database, as gathered in Figure 1. The former, also referred to as external validation, comprises a varied collection of 3865 CHON molecules corresponding to the near-equilibrium chemical space (further details can be found in the SI, section S5). Such a database has been proven able to offer an unbiased picture of the prediction abilities of the NN models. Hence, the error metrics evaluated within the latter should provide a reliable and trustworthy estimation of the actual performance of the predictions.

As shown in Figure 1, although all of the errors are roughly centered at the ideal values, the offsets are scattered to different extents, depending on the chemical nature of the atom. Indeed, this becomes even more evident if one takes a look at the parameters of the normal distributions fitted to that data (with parameters μ and σ), collected in Table 1.

**Table 1:** Mean (μ) and Standard Deviation (σ) of the Error Distributions for the Validation Data Sets in NNAIMQ^a^

	C	H	O	N
μ	3.77 × 10^–5^	–2.98 × 10^–4^	–1.97 × 10^–4^	–2.83 × 10^–4^
σ	1.54 × 10^–2^	8.98 × 10^–3^	1.11 × 10^–2^	2.21 × 10^–2^

a All values are reported in electrons.

It is reasonable to assume that the atomistic predictions coming from heavily dispersed error distributions (such as the one found for *N* atoms) are likely to contribute more to the total molecular charge error. We have thus decided that they should be subjected to larger corrections (η), defining weights proportional to the σ parameter of each error distribution. Such a strategy offers an approach analogous to that of Bleiziffer et al.^27^ Pushing these ideas a bit further, we have found it rather easy to derive different intuitive functional forms to relate the weights (*w*) to representative statistical and chemical parameters of the predictions. In this work, we have thus implemented a number of different *w* flavors for an *N*-atom system. For instance, the behavior of the atomic charge of those atoms bearing a larger nominal electron count is more likely to withstand to a larger extent the corrections made upon equilibration. In this way, the weights can be made proportional to the electron counts (*Z* – *q*^pred^):
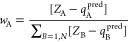
6Analogously, it is reasonable to assume that, given the lower prediction accuracy found for the heteroatoms (O,N) when compared to the lightest species (C,H), the corrections can be made proportional to the corresponding electronegativities (χ):
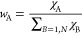
7

Altogether, and following an analogous approach, we have derived a total of 11 routines for the assignment of the corresponding weights, as gathered in Table. 2.

**Table 2:** Algorithm Used to Assign the Atomic Weights Throughout the Different Charge Equilibration Schemes^a^

equilibration scheme	atomic weight (*w*)
1	1/*N*
2	|*q*_A_^pred^|/(∑_*B*=1_^*N*^|*q*_B_^pred^|)
3	[*Z*_A_ – *q*_A_^pred^]/(∑_*B*=1_*^N^*[*Z*_B_ – *q*_B_^pred^])
4	χ_A_^S^/(∑_*B*=1_^*N*^χ_B_^S^)
5	χ_A_^P^/(∑_*B*=1_^*N*^χ_B_^P^)
6	σ_A_/(∑_*B*=1_^*N*^σ_B_)
7	|μ_A_|/(∑_*B*=1_^*N*^|μ_B_|)
8	(|μ_A_|·σ_A_)/(∑_*B*=1_^*N*^[|μ_B_|·σ_B_])
9	(|μ_A_|·[*Z*_A_ – *q*_A_^pred^]/*r*_A_)/(∑_*B*=1_^*N*^|μ_B_|·[*Z*_B_ – *q*_B_^pred^]/*r*_B_)
10	(|μ_A_|·[*Z*_A_ – *q*_A_^pred^])/(∑_*B*=1_^*N*^|μ_B_|·[*Z*_B_ – *q*_B_^pred^])
11	(|μ_A_|·σ_A_·[*Z*_A_ – *q*_A_^pred^]/*r*_A_)/(∑_*B*=1_^*N*^|μ_B_|·σ_B_·[*Z*_B_ – *q*_B_^pred^]/*r*_B_)

a Further details about the main parameters of each charge equilibration kernel can be found in the SI, section S5.2.

Besides these strategies, relying on different chemical or statistical features of the data, it should be mentioned that two additional approaches have been proposed. The latter are grounded in an iterative procedure that draws noise from the Gaussian distribution of each NN model to correct the corresponding partial charges (see SI section S5.2). However, given their computational cost and debatable performance, they will not be discussed in detail in the main manuscript. It is also worth mentioning that the redistribution of the excess molecular charge by these charge equilibration schemes will ensure the exact reconstruction of the molecular observables at the expense of (probably) increasing the noise in the atomistic predictions. Thus, special care should be taken when equilibrating the partial charges, as large correcting factors can partially hinder the quantum chemically accurate trends offered by the ML models, especially in highly polarized scenarios with a large excess of molecular charge. Finally, in order to evaluate the performance of the electron redistribution schemes used to correct the raw atomic charges, different error metrics will be used, particularly the mean absolute error (MAE), the root mean squared error (RMSE), and the Pearson correlation coefficient (see SI section S5 for more details). As a general trend, and unless otherwise specified, all errors are reported relative to the quantum chemical data, used as a reference.

## Algorithmic Details

**Figure 2:**
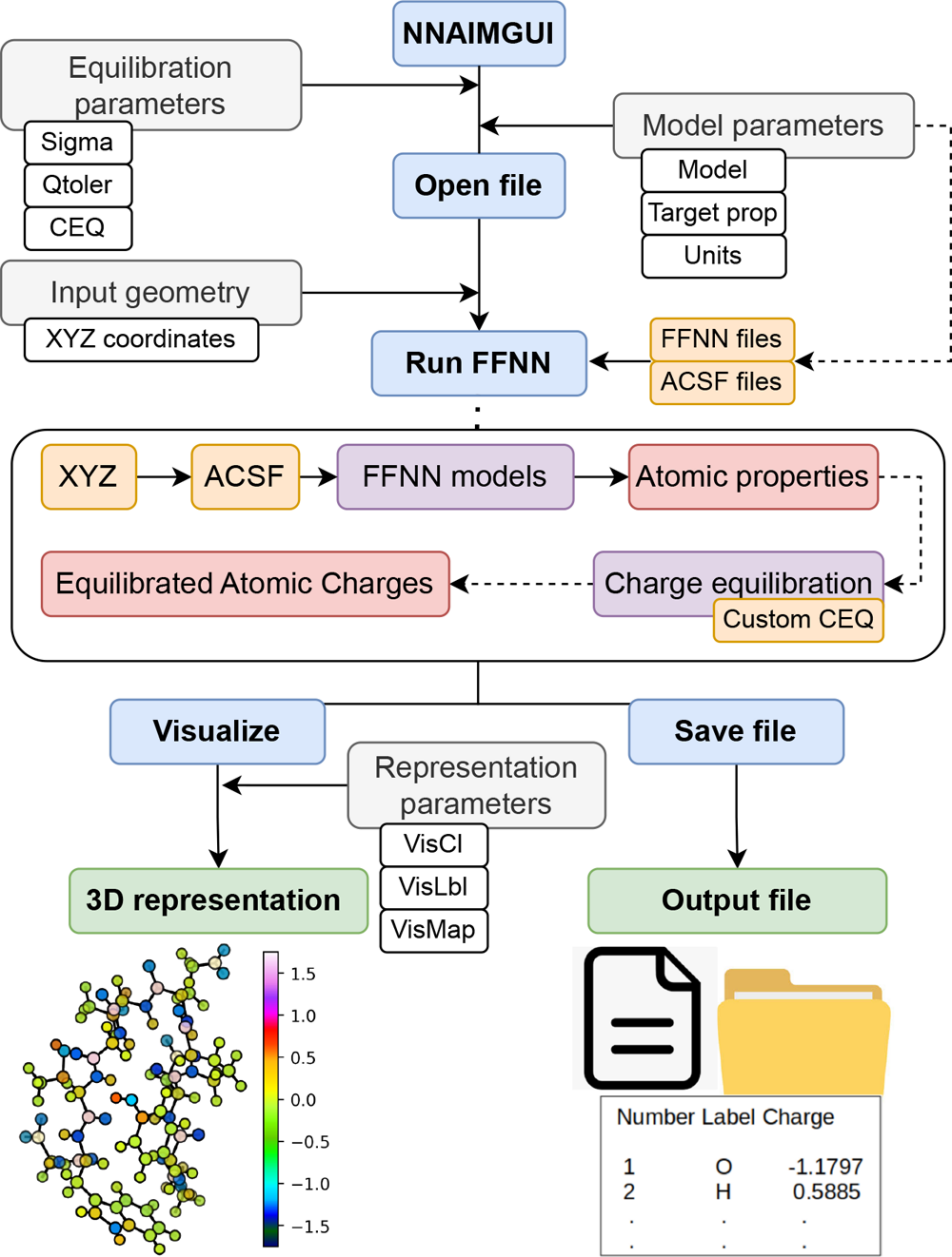
Flowchart for the main routines and features of the NNAIMGUI code. For the sake of clarity, the following color code has been used: gray for input features, orange for internal inputs, red for internal outputs, and green for output features. Additionally, the main routines involved in the calculation of the atomic properties are shown in violet.

Figure 2 gathers the general protocol involved in the calculation of equilibrated atomic charges (or any atomic property in general) with the NNAIMGUI code. The program requires three main input types:

• **Equilibration parameters**: These are related to the electron redistribution strategy to be employed when predicting QTAIM atomic charges: the factor used to bias the standard deviation of the normal distributions (Sigma), the maximum allowed residual molecular charge in the iterative methods (Qtoler), and the algorithm used to assign the weights (CEQ). Although NNAIMGUI has a wide collection of built-in charge equilibration approaches, custom algorithms can be also employed by selecting CEQ= −1. In the case of the latter, the path to the corresponding module must be also specified (for further details check the SI, section S5).

• **Model parameters:** Specify the main ML kernel used to obtain the raw atomic properties in the first place. Both built-in (NNAIMQ^13^) and tailor-made models can be used. If custom kernels are employed, which can be straightforwardly trained with NNAIMGUI, the path to the model folder (Model) must be given along with the name of the target property (Target prop) and property units (Units). The model folder contains both ACSF (input.ang, input.rad, and input.type) and FFNN files, the former indicate the parameters of the collection of Atom Centered Symmetry Functions (ACSF)^29^ used to describe the local chemical environments, whereas the latter gather the actual models along with some statistical parameters required for the standardization of the data. Further details on how to load custom ML kernels can be found in the SI and the NNAIMGUI GitHub.^30^

• **Input geometry:** The input file (with .xyz extension) gathering the atomic labels and positions must be provided in terms of general XYZ Cartesian coordinates (given in Å).

**Figure 3:**
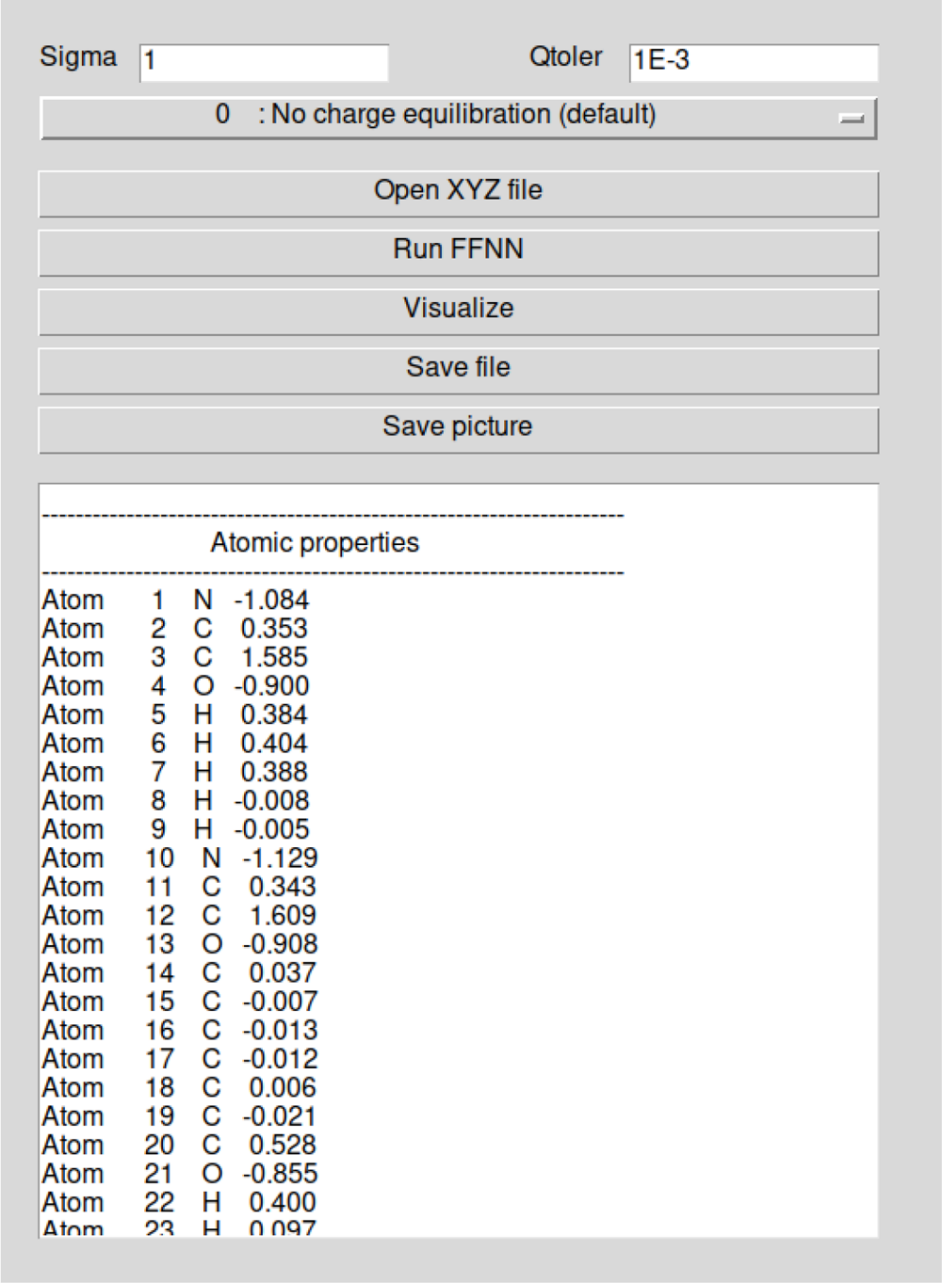
Main control and input dialogue of the NNAIMGUI code.

**Figure 4:**
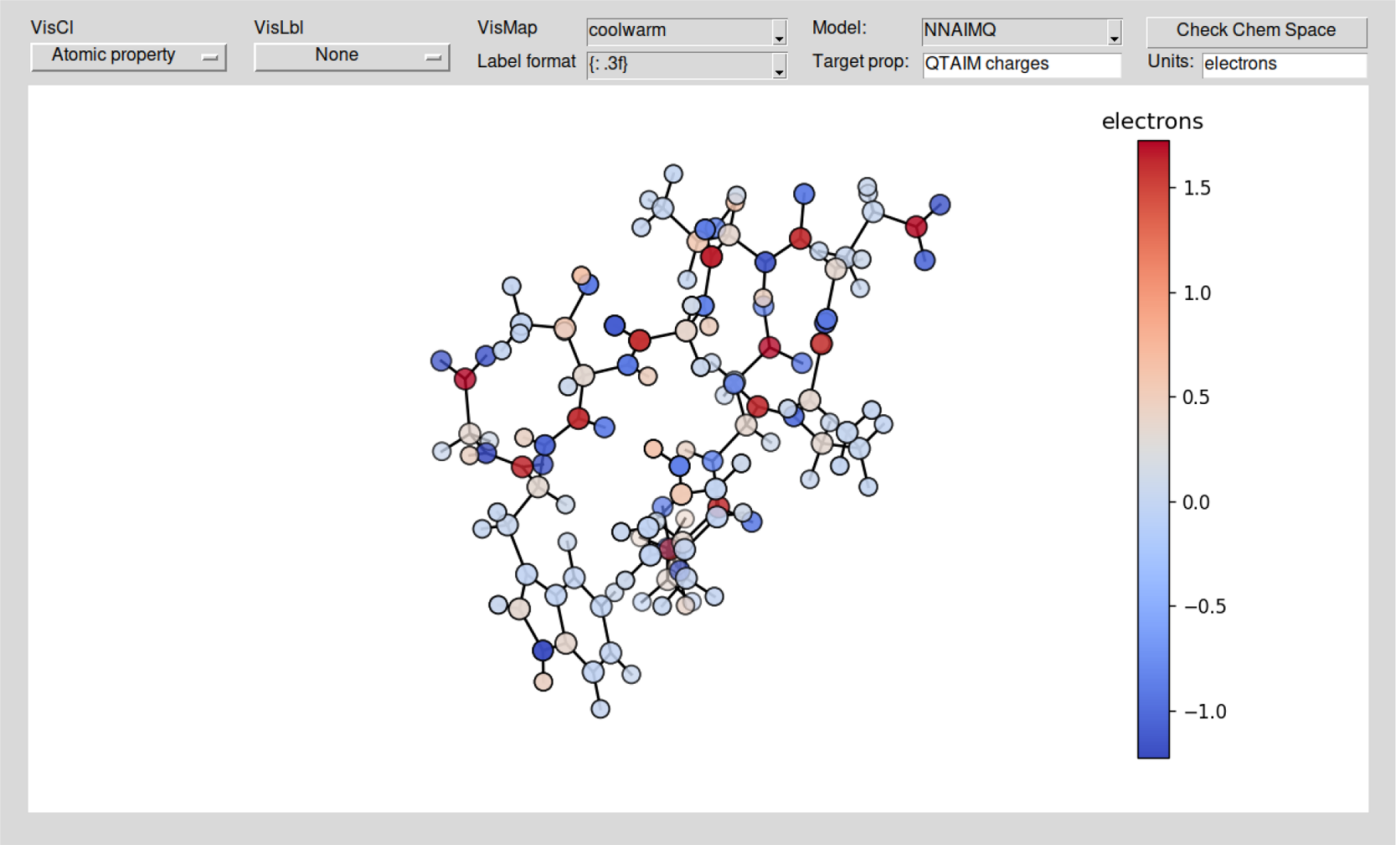
Visualization frame of the NNAIMGUI code showing the molecular representation of the previously estimated and corrected QTAIM atomic charges.

These three pieces of information constitute the basic input of the code, which can be specified either as arguments during the execution or through the main control dialogue of the GUI, as shown in Figure 3. The XYZ coordinates are then fed into the main ML kernel, which employs the internal SFC (Symmetry Function Calculator) module to compute the ACSFs used to describe the chemical environment in the near vicinity of each atom. Our SFC module can handle a wide spectrum of ACSF functional forms such that the chemical features can be optimized for particular applications. The resultant Atomic Environment Vectors (AEVs) are then received by the atomistic FFNNs which output the (nonequilibrated) atomic properties. If dealing with QTAIM atomic charges, the molecular error accompanying the latter can then be redistributed across the molecule using one of the aforementioned correction schemes in such a way that the desired equilibrated atomic charges are obtained. Finally, the predictions are shown on the standard output of the code. At this point, the results can be either saved as a .nnaim file or visualized with the built-in plotting utility, as shown in Figure 4. In the latter case, additional input information will be required: VisCl, VisLbl, and VisMap. The colors and labels of the atoms can be determined either by the atomic number (using the CPK coloring scheme) or by the value of the resultant atomic properties, as given by the visualization parameters VisCl and VisLbl. On the other hand, different color maps, as implemented in Matplotlib,^31^ can be chosen with the aid of the VisMap variable, making the analysis of the results easier.

**Figure 5:**
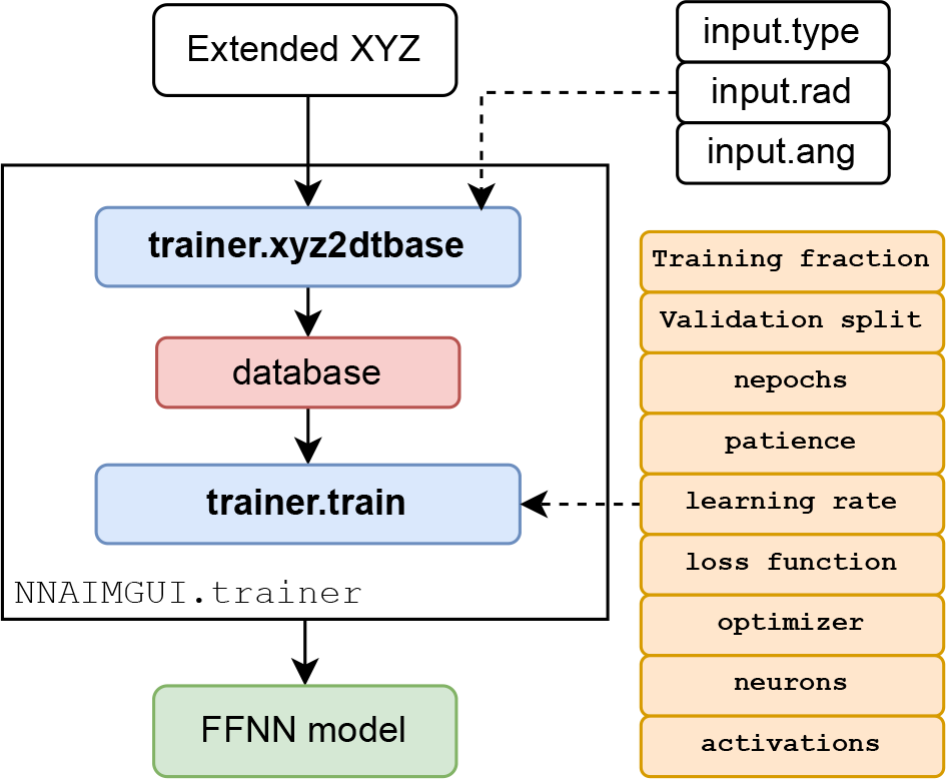
Flowchart of the NNAIMGUI trainer module showing how FFNN can be easily trained from plain XYZ files.

Furthermore, the built-in trainer module, shown in Figure 5, accounts for all of the basic steps required to create atomistic FFNN models from scratch. Starting from plain data in extended XYZ format, the chemical features are computed, and the database is then obtained with the xyz2dtbase function. The latter is parsed to the trainer.train, which, after setting the model architecture and hyper-parameters, trains the FFNNs on the desired target property. In this way, NNAIMGUI allows nonexperienced users to create ML models in a few lines of code, which can be later used to estimate and visualize atomic properties of interest. Further details on model creation can be found in the GitHub repository.^30^ Altogether, we consider that the here implemented features of the NNAIMGUI code could further aid the intuitive and straightforward determination of reliable QTAIM atomic properties, paving the way toward the extension of this and other QCT techniques within the noncomputational community of the chemistry realm.

## Computational Details

All of the additional geometry optimizations, single point calculations, intrinsic reaction coordinate (IRC) path analyses, and wave function generations used in this paper were performed in the gas phase at the M06-2X/def2-TZVP level of theory, as implemented in the *Gaussian 09* quantum chemistry package.^32^ The nature of the stationary points found along the reaction coordinates was characterized through the analysis of the eigenvalues of the Hessian matrix. Similarly, the QTAIM atomic charges were obtained from the corresponding wave functions with the PROMOLDEN^33^ code. As far as the proteins considered are regarded, single point calculations were performed on the structures available in the literature;^34,35^ further details can be found in the SI (section S4).

## Results and discussion

We comment now on the NNAIMGUI predictions of well-behaved QTAIM atomic charges when using different charge equilibration schemes. We have put to the test the performance of the code in a wide variety of scenarios, each of which will be discussed in a particular subsection. As a clarifying note, and for the sake of simplicity, the different electron redistribution strategies will be generally referred to by their previously assigned numbers (as shown in Table. 2). Additionally, the uncorrected atomic charges will be designated by the number 0. Similarly, notice that, regardless of the actual weight assignment strategy employed, atoms bearing large partial charges are likely to undergo larger corrections after charge equilibration, as reflected by eq 4. This becomes particularly prominent in the case of some heteroatoms, which, owing to their relatively large electronegativity, are more likely to be embedded in highly polarized local chemical fragments, resulting in large partial charges. Thus, it should not come as a surprise that the largest equilibration corrections will be found for N and O atoms.

### Interpolation Regimes

For the sake of convenience, we start by analyzing the performance of each charge equilibration scheme within the interpolation regime of the parent NN model, where even the raw values should be moderately well behaved. For such a purpose, the external validation (testing) data of the original work,^13^ gathering a total of 3865 CHON molecules, was used as our testbed model.

**Figure 6:**
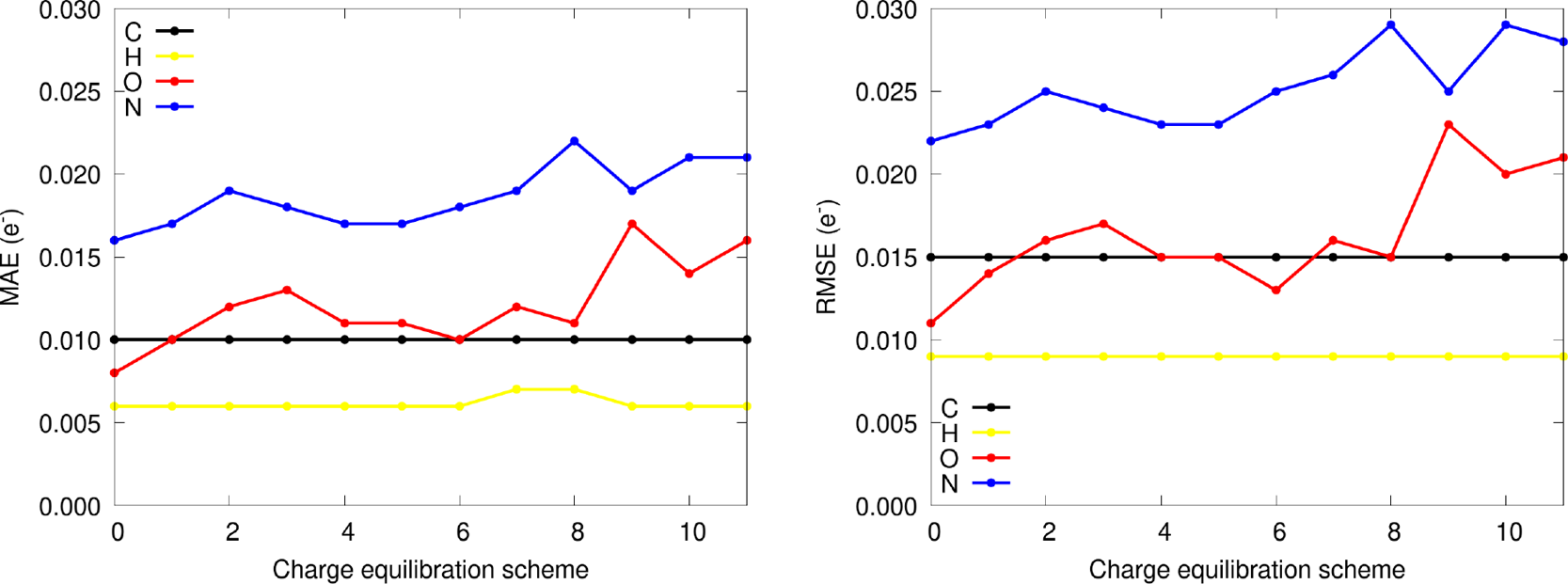
MAE and RMSE errors for the atomistic predictions of the NNAIMQ external validation data set in combination with different charge equilibration schemes.

Figure 6 shows the evolution of the errors in the atomic predictions (as given by the MAE and RMSE errors) as a function of the equilibration scheme. First of all, it is worth pointing out that the prediction errors follow the same behavior found previously for the uncorrected values, with heteroatoms (O, N) showing, on a general basis, lower accuracies. This finding arises from the lower number of training data available for the latter, which, coupled with the much more dispersed range of values visited by Q(O) and Q(N) (see SI section 1), results in larger estimation errors. On the other hand, as far as the charge equilibration scheme is regarded, two very different behaviors can be discerned: for C and H atoms, the error metrics are fairly stable, being almost independent of the electron redistribution strategy employed. This observation, with MAEs of 0.010 (C) and 0.006 (H) electrons on average, suggests that the corrections applied to the C and H atoms do not have a huge impact on the resulting qualitative and quantitative trends of their electron counts. Contrarily, the errors made by the equilibrated predictions of the O and N atoms are notably higher and fluctuate much more with the equilibration scheme used. This can be very appealingly explained after taking a look at the distribution of values of the QTAIM atomic charges (see SI section 1 for more details): whereas C and H tend to exhibit an almost neutral character on average, the partial charges of the heteroatoms (N and O) are generally clustered within a range of −1.5 and −1.0 electrons, owing to the much larger electronegativity of the latter. Hence, the almost locally neutral C and H atoms are subjected to much more subtle corrections, which allow the latter to recover, to a higher extent, the quantum chemically accurate trends reproduced by the ML models. Indeed, this is also reflected in the dispersion plots of the corrected partial charges (see SI section 1), which reveal that the equilibration of the NN predicted values increases, in a more prominent way, the dispersion of the O and N atoms, in agreement with the previously shown results.

To further investigate the actual performance achieved by each charge equilibration scheme, it may be enlightening to have a look at Table 3, which gathers the average errors as well as the Pearson correlation coefficients for the equilibrated atomic charges. As can be seen, all schemes seem to perform reasonably well, leading only to a very small worsening of the error metrics (e.g., the MAE increases by 0.001 electrons at most) with respect to the uncorrected values. This result is not surprising at all, as in interpolation regimes, the ML models are usually capable of reconstructing the neutral molecular character, giving rise to small Δ*Q* values and thus relatively innocent equilibration corrections. Nevertheless, those equilibration strategies relying on the standard deviation, the electronegativity, or even the straightforward homogeneous assignment of the weights offer the best performance on average. Indeed, the application of schemes 1, 4, 5, and 6 provides almost identical MAE and RMSE metrics to those found for the uncorrected atomic charges, while yielding quite decent *r* coefficients (0.996 on average). These findings indicate that those weighting schemes which promote larger corrections on the heteroatoms are more successful, something which fits well with intuition and the previously mentioned trends: the atomistic predictions of the N and O atoms are the least reliable ones, being likely to contribute the most to the total Δ*Q* and thus undergoing larger corrections.

**Table 3:** Global Errors, As Given by the L1 (MAE) and L2 (RMSE) Norms along with Pearson Correlation Coefficient (*r*) for Different Charge Equilibration Schemes

CEQ	MAE (e^–^)	RMSE (e^–^)	*r* (a.u.)	*r*^2^ (a.u.)
0	0.0079	0.0118	0.9963	0.9926
1	0.0081	0.0120	0.9962	0.9924
2	0.0083	0.0124	0.9959	0.9919
3	0.0083	0.0123	0.9961	0.9922
4	0.0081	0.0121	0.9962	0.9924
5	0.0082	0.0121	0.9962	0.9924
6	0.0081	0.0121	0.9962	0.9923
7	0.0083	0.0124	0.9962	0.9923
8	0.0083	0.0126	0.9962	0.9924
9	0.0086	0.0131	0.9962	0.9924
10	0.0085	0.0130	0.9962	0.9924
11	0.0086	0.0131	0.9962	0.9924

### Deep Extrapolation Regimes: the Particularly Problematic Case of Chemical Reactions

Besides testing the performance of the proposed charge equilibration schemes in interpolation regimes (where only minor corrections are usually applied), it is also crucial to test their reliability in more challenging scenarios. For such a purpose, the evolution of the equilibrated atomic charges throughout some of the chemical reactions (Rx) used to test the original version of NNAIMQ were studied (further information about the computational details employed for the determination of the corresponding reaction profiles can be found in the original ref (13)). Chemical reactions, involving bond breaking and bond formation typically explore situations that lie well within the extrapolation regimes of ML models. They are thus used to test their performance since, on a general basis, NN models are not expected to afford reliable predictions when extrapolating.

**Figure 7:**
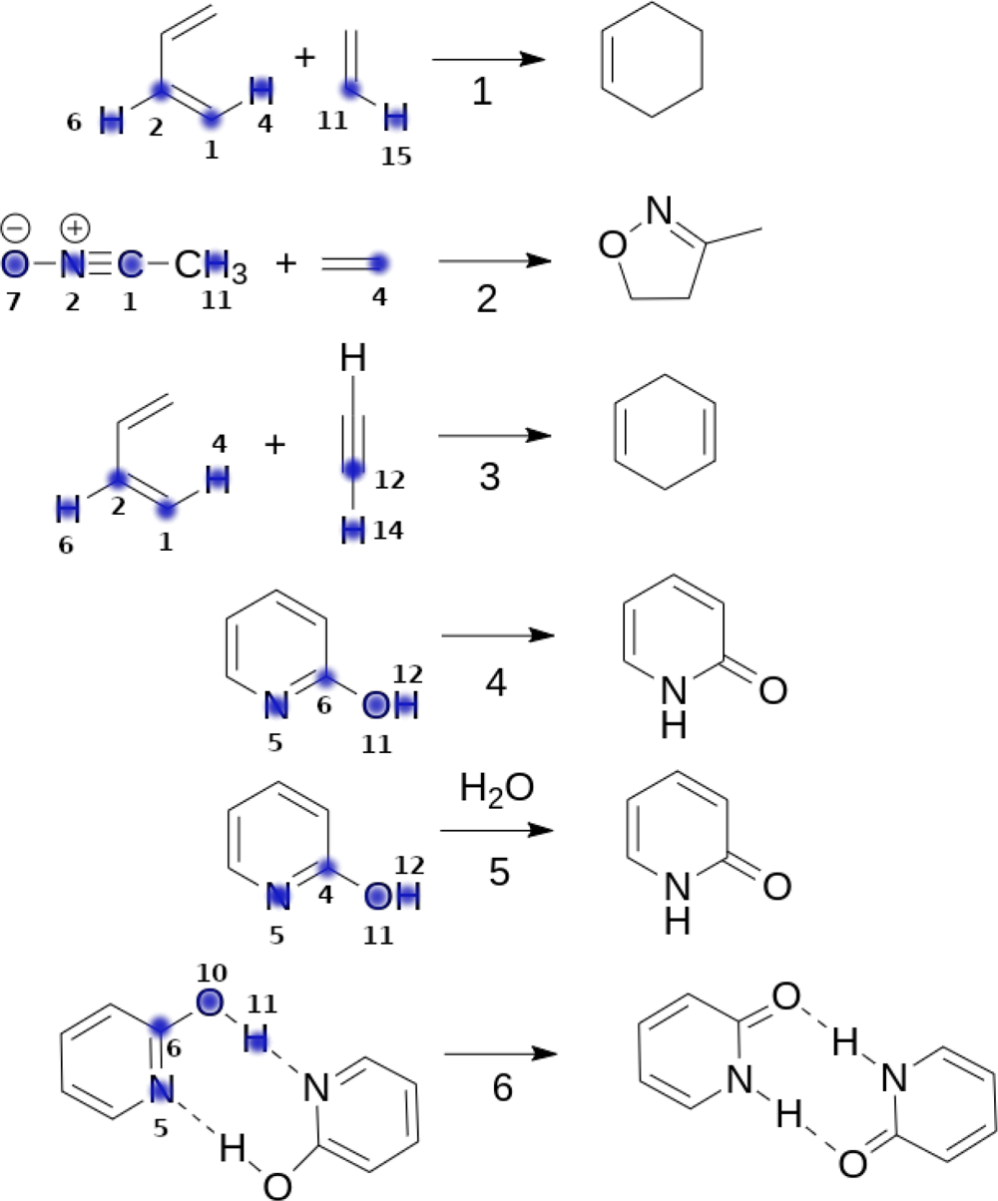
Testbed reactions examined in this work. (1) Diels–Alder cycloaddition between 1,3-butadiene and ethylene, (2) 1,3-dipolar cycloaddition between acetonitrile oxide and ethylene, (3) Diels–Alder cycloaddition between 1,3-butadiene and acetylene, (4) tautomerism of 2-hydroxypyridine, (5) water-catalyzed tautomerism of 2-hydroxypyridine, and (6) tautomerism of the 2-hydroxypyridine dimer. The most relevant atoms involved in each chemical transformation are highlighted in blue.

**Figure 8:**
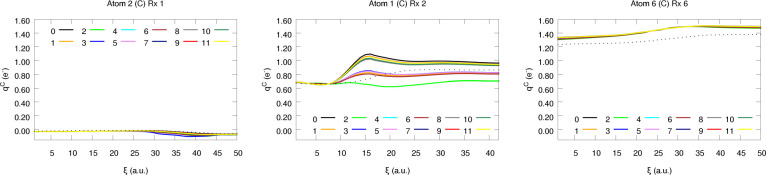
Atomic charges of a few selected C atoms along reactions 1, 2, and 6.

**Figure 9:**
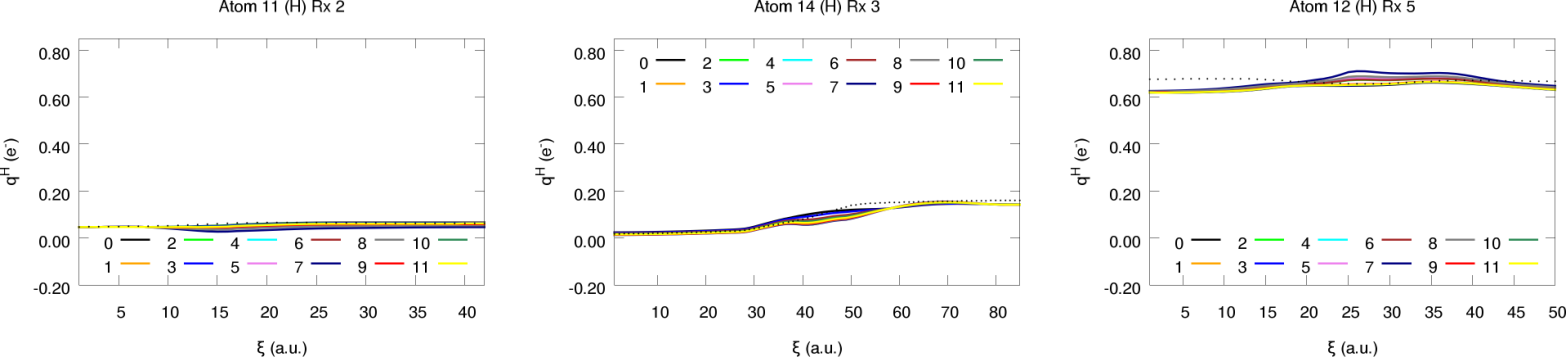
Atomic charges of a few selected H atoms along reactions 2, 3, and 5.

**Figure 10:**
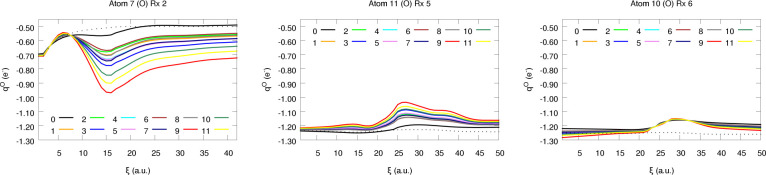
Atomic charges of a few selected O atoms along reactions 2, 5, and 6.

**Figure 11:**
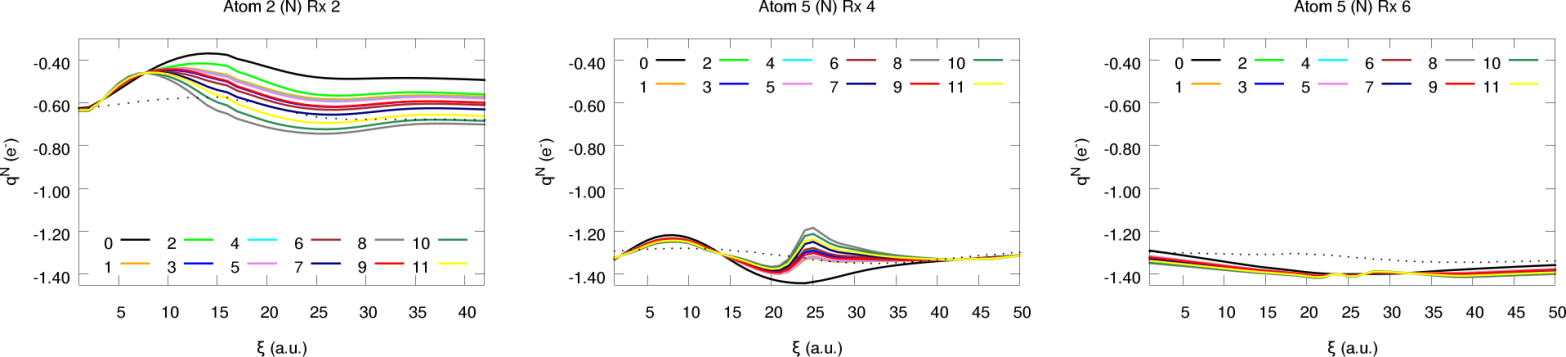
Atomic charges of a few selected N atoms along reactions 2, 4, and 6.

Figure 7 shows a sketch of the reactions that we have employed as testbed model. Besides the chemical transformations studied before, and for the sake of completeness, the Diels–Alder cycloaddition between 1,3-butadiene and acetylene (Rx 3) was also analyzed (see SI section S2 for more details). Altogether, these systems, combining both polar and nonpolar species, σ and π bond rearrangements, and inter- and intramolecular transformations, should be diverse enough to address the performance of the electron redistribution approach in common extrapolation regimes. The most relevant atoms, those which are likely to undergo a significant change in their electron count throughout the reactions, are highlighted in blue. Given the large amount of data, only a collection of representative atoms embedded in different chemical environments will be selected and discussed in detail for each element type, as gathered in Figures 8–11. For the sake of simplicity, the *x*-axis (ξ) is simply an integer reaction coordinate. In this way, we can get a grasp of the actual performance of all of the electron redistribution schemes in a wide variety of chemical scenarios. The trends for the remaining atoms can be found in the SI (section S2.3).

First of all, it is evident that the largest discrepancies between the equilibrated and the raw (CEQ = 0) values are usually observed in the vicinity of the transition state (TS) structures (located at ξ = 31, 17, 50, 25, 25, and 25 for Rx 1–6, respectively). Such a finding is in perfect agreement with our intuition: TSs are usually those configurations differing the most from the equilibrium geometries used to train the NN models and thus represent the most extreme scenario of extrapolation. In this regime, ML predictions are likely to fail, with atomic errors accumulating instead of canceling, which gives rise to a large Δ*Q* that in turn yields substantial correcting factors (η). Indeed, the spurious accumulation of molecular charge can easily yield nonphysical trends in the atomic charges, as evidenced by some of the kinks found in Figures 10 and 11. This is also reflected in the difference between the uncorrected atomic charges and the quantum-mechanically computed ones, as represented by black points (·) in the aforementioned figures. Actually, the extent of the offset between the latter values follows essentially the behavior of Δ*Q*, as gathered in SI S2.1. Although we will not enter into more details, the raw predictions show, in general, a good correspondence with the QM data, represented as dotted lines, at least on a qualitative level. Such a result is only slightly worse for N atoms, given the considerably reduced number of training points used for the construction of its atomistic NN model as compared to other species such as C and H.

**Figure 12:**
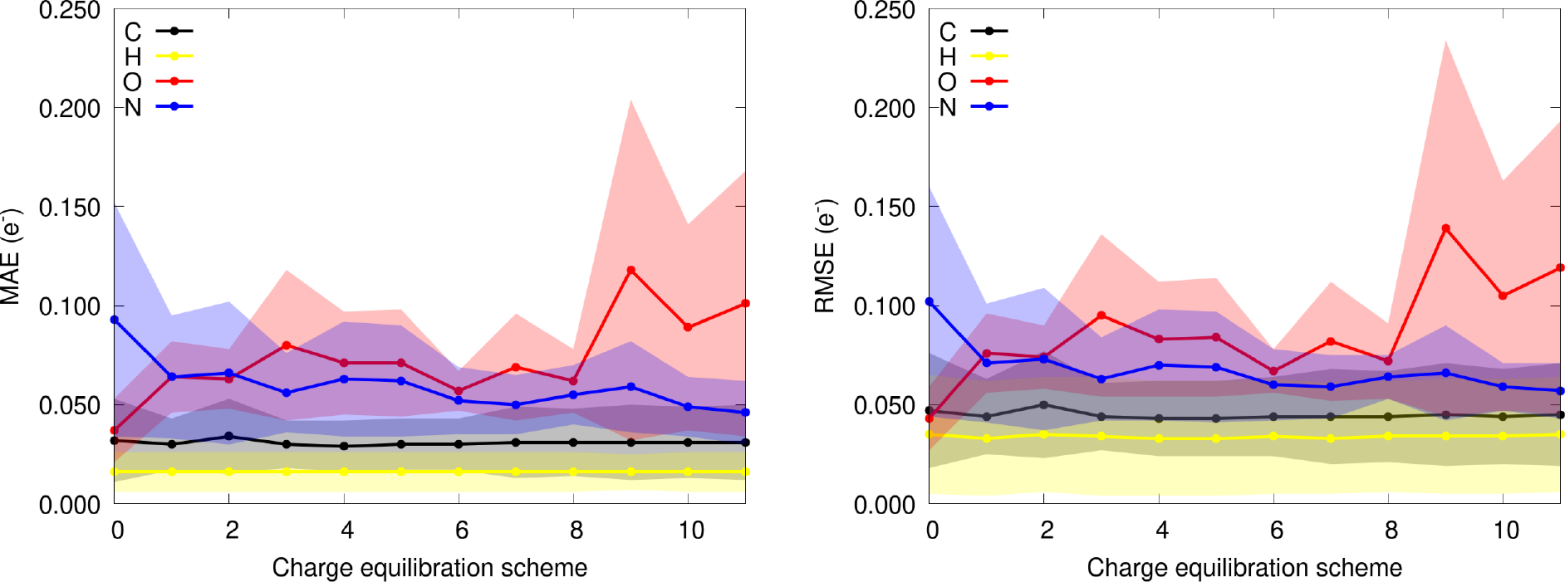
Mean MAE and RMSE errors for the equilibrated atomistic predictions in extrapolation regimes. Partially transparent filled curves indicate the standard deviation of the results, as computed with all of the chemical reactions here studied.

Now, let us test the reliability of the resultant equilibrated charges for each chemical element in the set. For C and H atoms, all schemes afford nearly equivalent trends, in agreement with the previously observed results for the interpolation data (see Figure 6). This can be, once again, attributed to the moderate nominal charge values usually found for *q*_C_ and *q*_H_. Additionally, it is also worth pointing out that the behavior found for the different schemes seems quite homogeneous across the different chemical environments found in these reactions. Something which becomes even more evident if we take a look at the average performance, reported in terms of the L1 and L2 based error metrics, of each electron redistribution strategy, as gathered in Figure 12. Not only is the MAE of the C and H atoms quite independent of the equilibration strategy employed, exhibiting mean values of 0.03 and 0.02 electrons, but so are the standard deviations of the latter, as represented by the shadow of the filled curves.

Altogether, these results suggest that a good performance is likely to be achieved by any of the equilibration schemes explored in the extrapolation regime of any of the C or H atoms. Indeed, and as a general trend, distributing Δ*Q* does not seem to significantly worsen the prediction accuracy of these two species, as reflected by the almost negligible increase in the errors found for the equilibrated results when compared to the raw ones (CEQ = 0 in Figure 12). It may be noted in passing, however, that although the same evolution of the errors is found for the interpolation and extrapolation regimes, the absolute values for the former are noticeably lower than for the latter, with a scale factor of ∼2. Such a result, also observed in the uncorrected data, is not surprising as the accuracy of the models is known to significantly drop when extrapolating.

Considerably different findings are found for N and O, being much more sensitive to the weight assignment strategy (see Figures 10 and 11). In parallel to the observations already reported under interpolation conditions, larger errors are found when these atoms are compared to their lightest analogs (C, H). This is not restricted to raw predictions, but the prominent corrections arising from the large *q*_O_ and *q*_N_ values also have a non-negligible impact on the dispersion of the latter. Nonetheless, and to our surprise, redistributing Δ*Q* does not always increase the prediction errors, as proven by the partial charges of the N atoms (see Figure 12). Despite being counterintuitive at first glance, this result can be explained attending to the particularly bad extrapolation abilities of the nitrogen NN model, something which, coupled to the unfamiliar chemical environment visited throughout the reactions, inevitably increases the uncertainty of the predictions. This lack of accuracy can easily result in heavily polarized N atoms (bearing abnormally high or low electron counts) which in turn build up large Δ*Q* values. In this scenario, equilibrating the charges may be particularly beneficial as it counteracts the bias of the model, resulting in the spurious decrease of the prediction errors. This effect can be readily seen in Figure 11, where the corrected atomic charges are closer to the quantum chemically computed ones than the starting raw values. On the other hand, opposed trends are observed for the O atoms: charge equilibration results in a slight worsening of the *q*_O_ values, as evidenced by the moderate increase of the L1 and L2 metrics. Indeed, and unlike in the case of nitrogen, the evolution of the prediction errors with the scheme employed is essentially analogous to that found for the reference database (Figure 6). The success of the weight assignment strategies in this case depends to a much higher extent on the chemical environment, as proven by the increase in the standard deviation of the errors. This is also observed, although in a much more subtle way, for the N atoms. In this context, it may be worth highlighting that the highest discrepancy between different electron redistribution strategies is found for Rx 2: the prominent polarization of the system, as reflected by the large partial charges, in combination with the significant Δ*Q* values (≈ 0.5*e*) reached in the vicinity of the TS (see SI section S2.1) gives rise to a quite noticeable deviation between the corrected and the raw values, explaining the behavior observed in Figures 10 and 11. The previously mentioned trend is furthermore enhanced by the considerably lower number of data points available for O and N when compared to C and H. Notice that this is not a result of a biased selection of the training data, but it is instead a consequence of the natural occurrence of these elements in common molecules. In fact, in organic chemistry, these heteroatoms are usually embedded in local functional groups (e.g OH, NH_2_) and thus do not constitute the main skeleton of the molecules. Finally, and as far as the strategy used to assign the atomic weights is regarded, it seems that charge equilibration scheme 6 offers the best performance, in agreement with the results found in interpolation regimes.

### Molecular Dynamics (MD) Simulations

**Figure 13:**
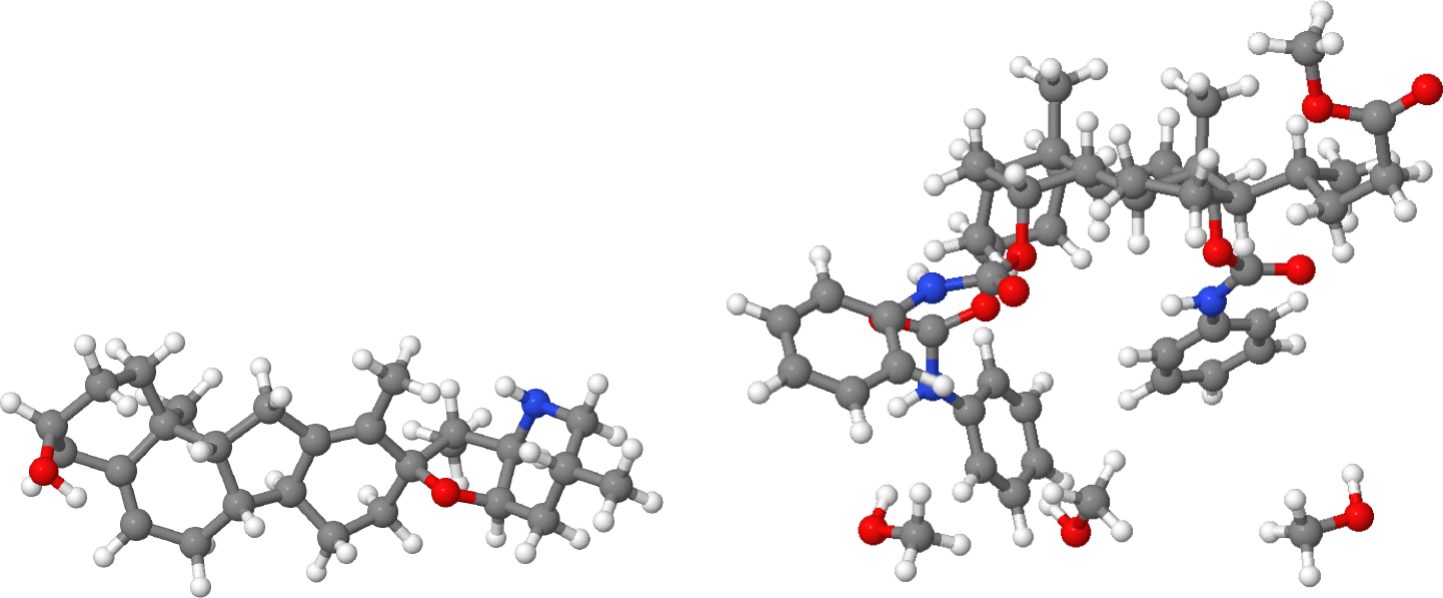
Cyclopamine molecule (left) and the {steroid: [CH_3_OH]_3_} supramolecular complex (right).

Besides the quantitatively correct values many times employed in the context of computational chemistry, very valuable chemical insights can be often drawn from qualitative analyses. It is thus important to explore how the application of the charge equilibration schemes alters the response of the electron count of an atom to a perturbation of its nearby chemical environment, as measured by the relative changes in the atomic charges, Δ*q*. With that aim, the behavior of the equilibrated partial charges along MD simulations of some medium to large size systems, a cyclopamine molecule and a steroid-based supramolecular complex {steroid: [CH_3_OH]_3_} (see Figure 13), was studied. Further information about these systems and the computational details involved in their MD simulations can be found in ref (13). The combination of the near-equilibrium structures visited throughout the MD simulations and the large molecular size places these systems in a particularly sweet and convenient spot between interpolation and extrapolation regimes. Additionally, given the large computational cost of the conventional QM calculations required to obtain the QTAIM atomic charges explored during the simulation, the performance metrics will be reported relative to the raw values (CEQ = 0).

**Figure 14:**
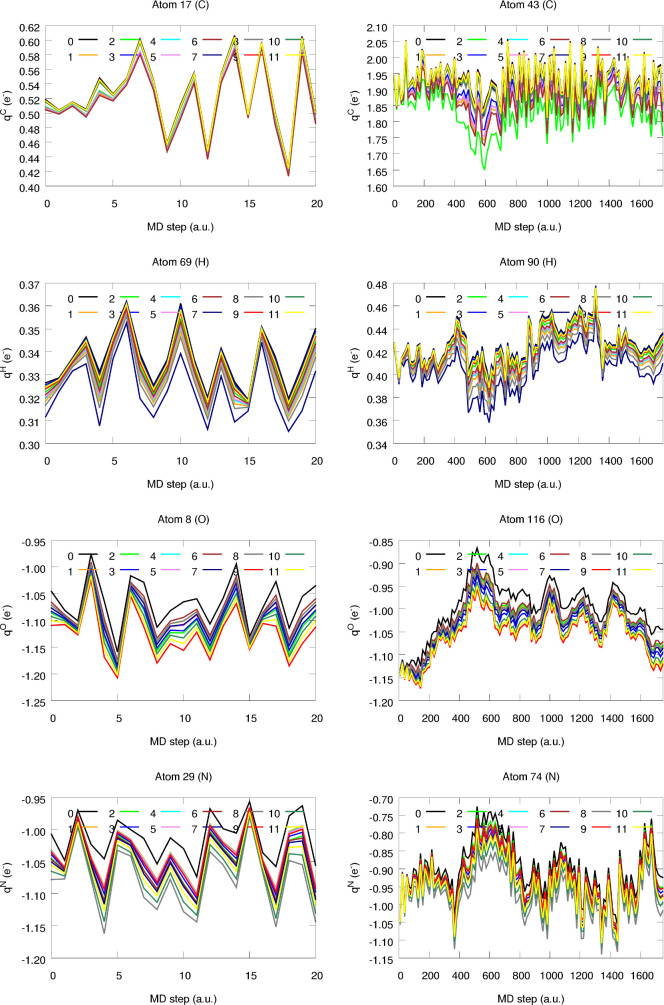
Evolution of the equilibrated atomic charges of relevant atoms of the cyclopamine (left) and steroid (right) molecules throughout the MD simulations. The numbering shown in the XYZ Cartesian coordinates of both molecules, gathered in the SI, section S3, was employed.

Let us start by analyzing the evolution of the local electron counts throughout the simulations, as gathered in Figure 14. For the sake of convenience, a selection of atoms belonging to widely different chemical environments was selected. Moreover, and accounting for the large amount of data-points available for the steroid supramolecular complex, only bin-averaged data obtained by averaging every 15 MD steps are reported. Despite the evident offset between the raw and the corrected charges, all equilibration schemes seem to appropriately maintain the qualitative trends exhibited by *q*. It is worth noticing that this behavior is not exclusive to the previously shown collection of atoms but that similar results are obtained for the remaining molecular constituents (see SI section S3). This clearly proves that distributing Δ*Q* is unlikely to significantly alter the trends in the individual charges. In this way, the resultant corrected partial charges do hold the chemically intuitive behavior obtained from the quantum chemical calculations. As an example, one can have a look at the O atom of the OH group of one of the CH_3_OH molecules that binds to the active pocket of the steroidal scaffold (e.g., O 116 in Figure 14). As shown, the formation of fairly strong intermolecular interactions (e.g., H-bonding) triggers a quite prominent electron redistribution between the molecules. More specifically, as the substrate approaches one of the carbamate moieties of the steroid skeleton, a non-negligible decrease in the electron count of the aforementioned O atom occurs, very clearly reflected by the decrease of *q*_O_. Considerably smaller fluctuations are found throughout the simulation of the cyclopamine molecule, where atomic charges are only slightly perturbed by the normal vibration modes of the molecule. Despite being subtle, these oscillations can be appealingly recovered by all of the here explored charge equilibration schemes.

**Figure 15:**
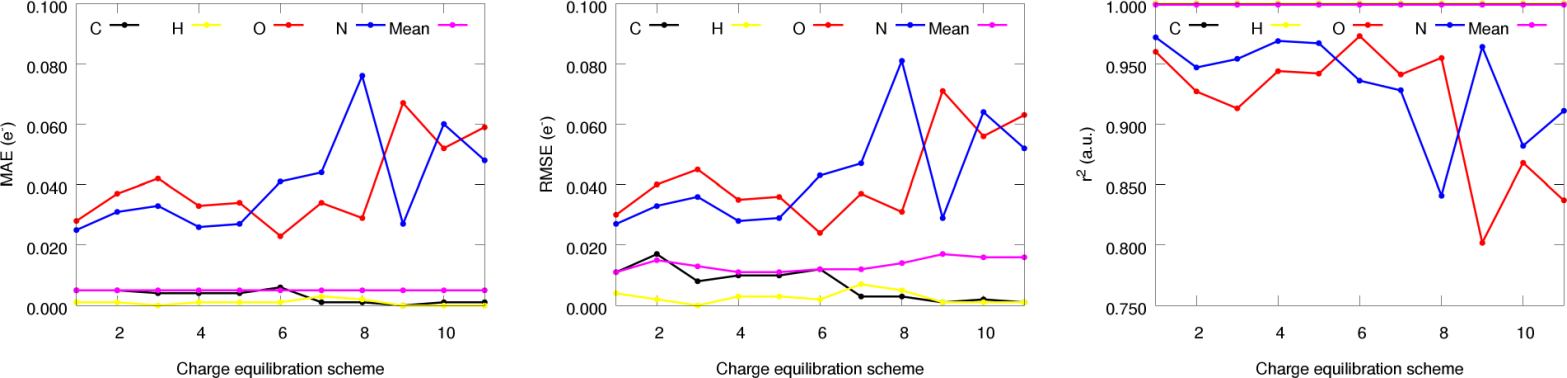
MAE, RMSE, and Pearson’s correlation coefficient (*r*^2^) of the equilibrated partial charges obtained with different charge equilibration schemes. All reported values are the average obtained in the MD simulations examined in this work.

Although all of the weight assignment approaches recover quite successfully the qualitative trends in the partial charges, a more robust comparison is provided by the analysis of the mean correlation and error metrics, as collected in Figure 15.

Once again, it seems that applying larger corrections to the heteroatoms achieves the best average performance. Indeed, equilibration schemes 1, 4, 5, and 6 show the best results, displaying decent error metrics as well as good correlation coefficients for all of the chemical elements.

## Applications: Adequately Behaved QTAIM Charges in Large Systems

**Figure 16:**
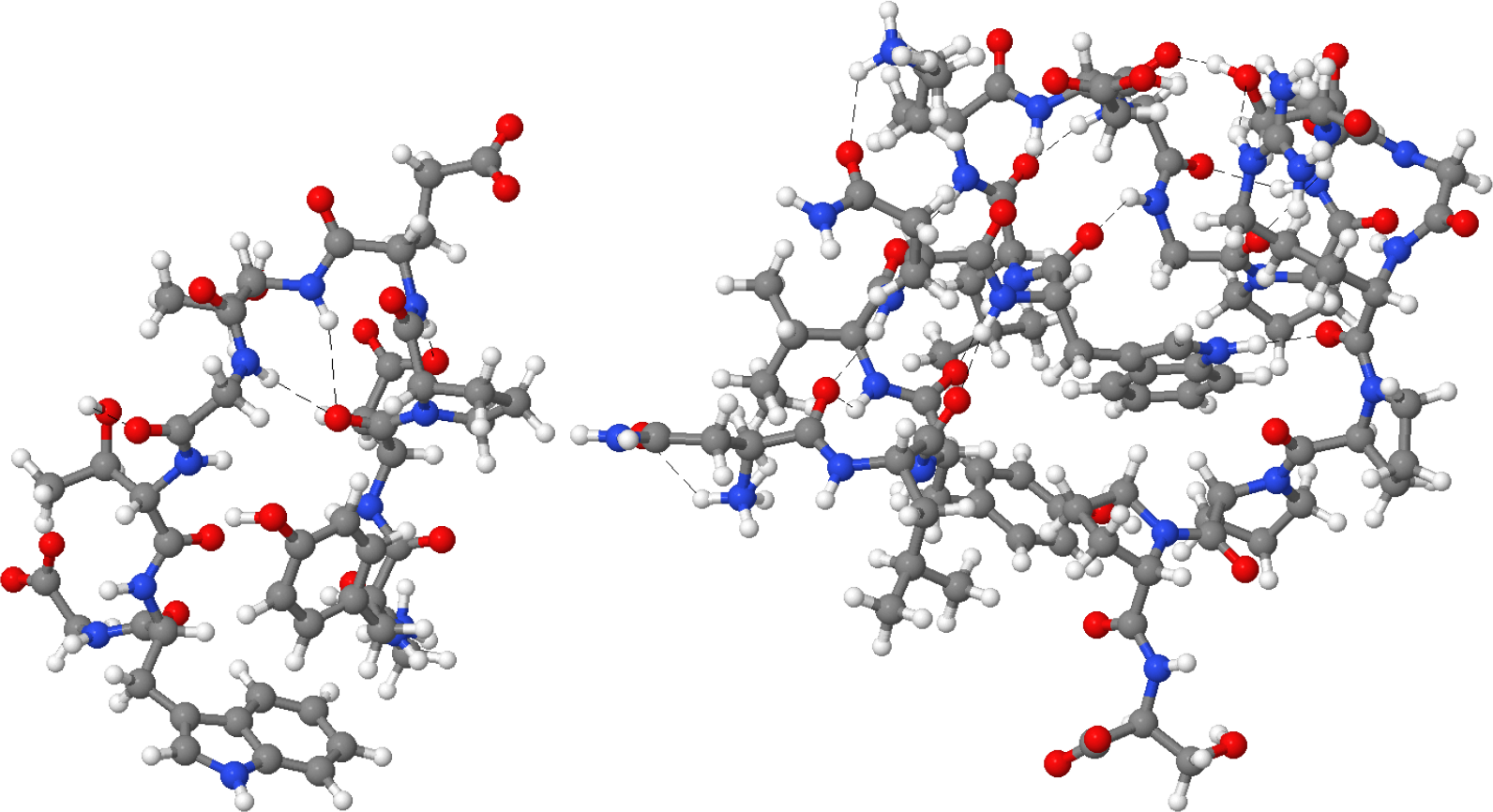
Ball and stick representation of Chignolin (left) and TC5b (right) proteins.

As a final proof of concept, we use NNAIMGUI in a real, and more challenging, scenario. For such a purpose, we decided to predict the QTAIM atomic charges of a collection of CHON mini-proteins: Chignolin^34^ and TC5b,^35^ as shown in Figure 16. Both represent examples of tailor-made protein models which have been successfully proposed in the literature^36−38^ as testbed systems to study the nature of a wide variety of supramolecular phenomena. Hence, these scaffolds, bearing 138 and 309 atoms, respectively, are particularly convenient to test the applicability of our code for larger systems.

**Figure 17:**
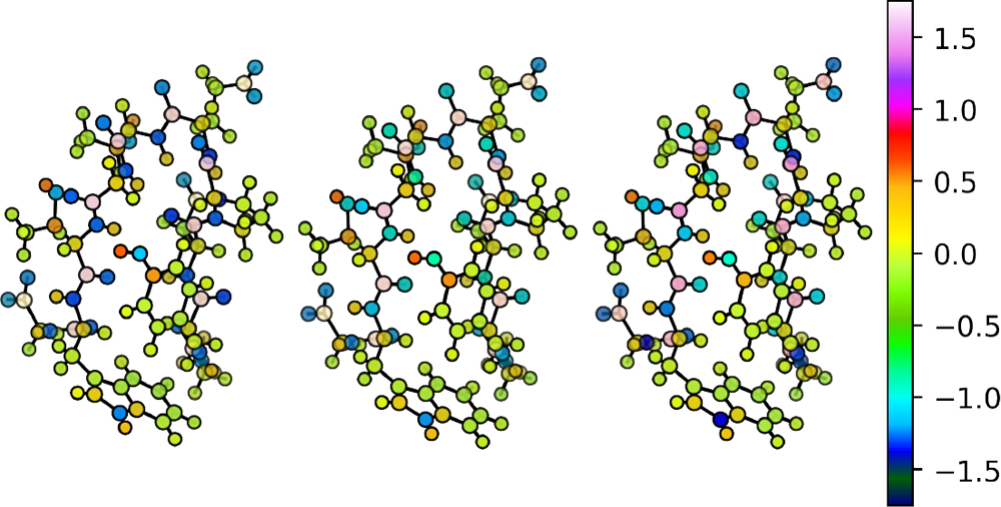
Distribution of partial charges (in electrons) in the Chignolin molecule as computed by electronic structure calculations (left) and by the NNAIMGUI code. For the latter, the raw (center) and equilibrated (right) values are shown.

**Figure 18:**
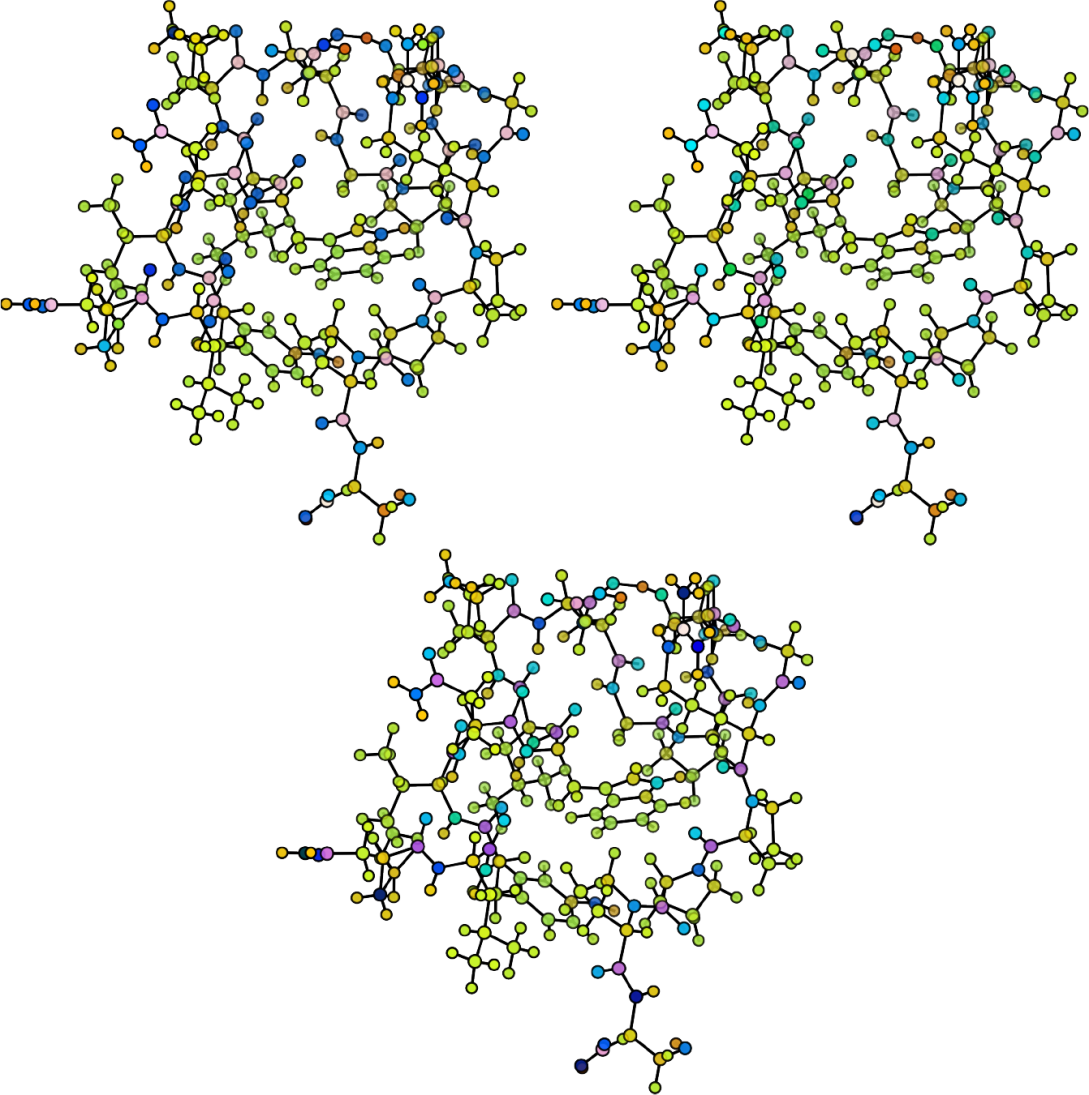
Distribution of partial charges (in electrons) in the TC5b molecule as computed by electronic structure calculations (left) and by the NNAIMGUI code. For the latter, the raw (center) and equilibrated (right) values are shown.

Figures 17 and 18 gather the distribution of partial charges, given in electrons, for both proteins as computed according to QM and ML methods. All of the molecular graphs have been rendered with the NNAIMGUI code. Considering the superior performance of heteroatom penalizing schemes, and in particular those relying on the standard deviation of the FFNN error distributions (CEQ = 6), we will restrict our discussion to this equilibration algorithm.

**Figure 19:**
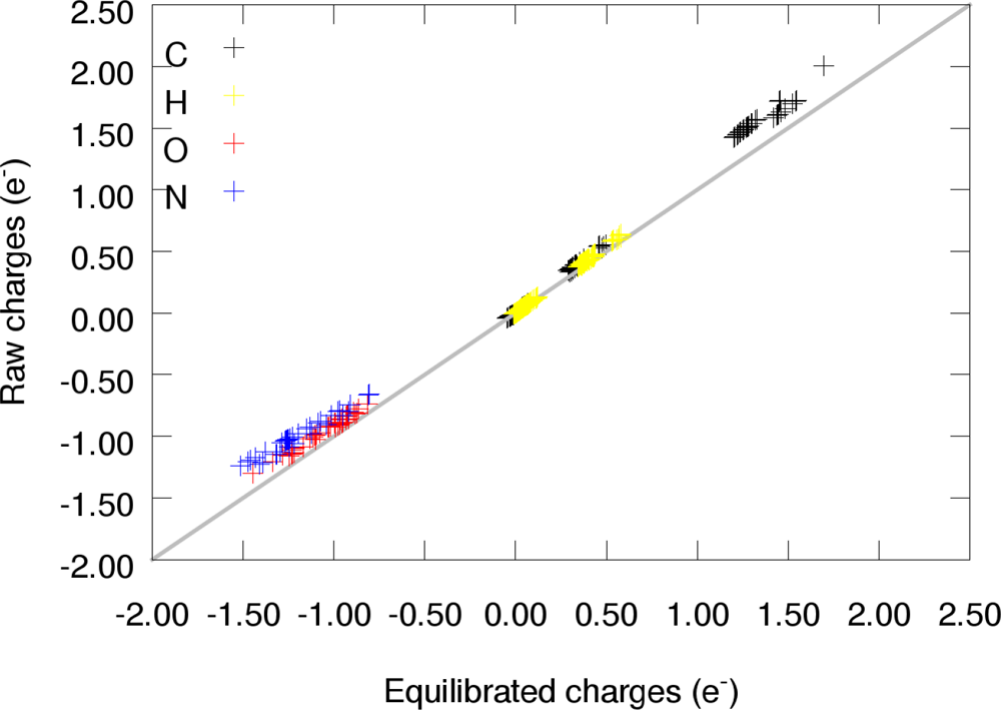
Dispersion of the equilibrated vs raw atomic charges of both proteins as predicted by NNAIMGUI.

From the aforementioned figures, it becomes evident that the ML model is able to appealingly reproduce the quantum chemically computed distribution of partial charges for both proteins. Moreover, correcting the atomic charges with the aid of the σ-based weight assignment (CEQ = 6) does not seem to significantly alter the results. It should be pointed out that such a trend is not exclusive to this particular correction scheme, but that similar results can be found for the remaining ones (see SI section S4 for more details). Analogously, and in agreement with the results found in previous sections, the atomic errors are not affected up to a large extent by the redistribution of the excess molecular charge. This is clearly reflected by the linear and narrow dispersion found in Figure 19, the correlation plot between the raw and equilibrated atomic charges for both supramolecular structures.

**Figure 20:**
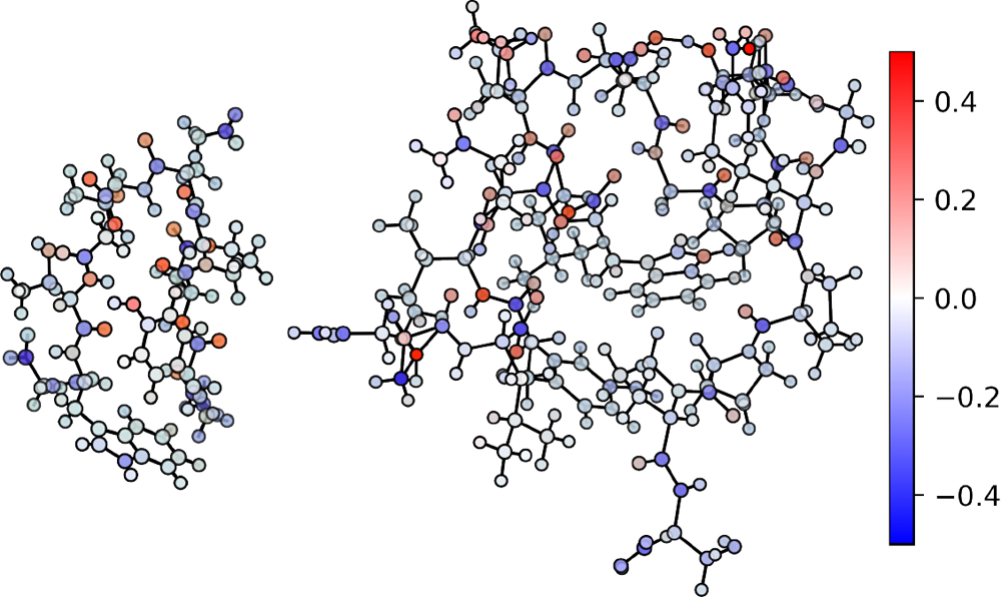
Distribution of the errors made by NNAIMGUI (CEQ = 6) in the estimation of the partial charges of the Chignolin (left) and TC5b (right) proteins. All of the errors, reported in electrons, are computed as the difference between the predicted and the computed values.

Finally, and as can be seen in Figure 20, the prediction accuracy of the models is not heavily biased by the application of the charge equilibration strategy. As expected, the highest errors are usually observed in the vicinity of the heteroatom containing scaffolds, such as C=O or NH_2_ groups, owing to the already mentioned limited extrapolation abilities of the ML atomistic models for O and N atoms. Such a distribution of errors does not arise from the application of the equilibration step, but it is rather intrinsic to the NN models. Indeed, the results found for the uncorrected values (see SI section 4) reveal that correcting the atomic charges may actually soften the topology of the error distributions. Something which is particularly pronounced for the O and N atoms and can even result in a subtle improvement of the accuracy of the predicted atomic charges, as shown in Figure 17, for instance.

## Conclusions

NNAIMQ offers a fairly robust approach to compute QTAIM atomic charges at a much reduced computational cost. However, the independent nature of the atomistic predictions does not guarantee the exact reconstruction of the total molecular charge. In this work, we have explored the possibility of correcting the raw ML predictions with a simple charge equilibration scheme which can be controlled by tuning the weight attributed to each atom. These features have been implemented in a new Python based code, named NNAIMGUI, which is moreover equipped with a Graphical User Interface (GUI). The results of this work suggest that, though simple, the here explored charge equilibration provides exactly canceling atomic charges which still hold the quantum-chemically accurate behavior recovered by the ML models. Besides partial charges, NNAIMGUI can deal with any other atomic property and chemical space of choice, allowing the user to easily build and apply their own FFNN models trained for any particular applications. Altogether, the findings obtained in this work coupled to the flexibility and visualization abilities of our code prove that NNAIMGUI could significantly ease the estimation of well-behaved QTAIM atomic properties even for noncomputational chemistry users, paving the way toward the rigorous study of computationally demanding problems on feasible time scales.

## Data Availability

The data and code supporting this work are available at the NNAIMGUI GitHub repository (https://github.com/m-gallegos/NNAIMGUI).

## Supporting Information Available

(Sections 1–4) Results obtained when evaluating the performance of the code in interpolation and extrapolation (chemical reactions) regimes, molecular dynamic simulations, and supramolecular systems; (section 5) algorithmic details of the NNAIMGUI code (the latter is freely available in the main NNAIMGUI GitHub project: https://github.com/m-gallegos/NNAIMGUI) (PDF)
