# Supplementary material for: Developing a User-Friendly Code for the Fast Estimation of Well-Behaved Real-Space Partial Charges
Source: J Chem Inf Model. 2023 Jun 20;63(13):4100–14. doi: 10.1021/acs.jcim.3c00597 (PMC10336973; doi:10.1021/acs.jcim.3c00597)
Supplement: Supplementary file 1 — ci3c00597_si_001.pdf [file ci3c00597_si_001.pdf]

Developing a User-Friendly Code for the Fast Estimation of Well-Behaved Real-Space Partial Charges

Miguel Gallegos[1] and Ángel Martín Pendás*[1]

[1]Department of Analytical and Physical Chemistry, University of Oviedo, E-33006, Oviedo, Spain.

*To whom correspondence should be addressed : Prof. Ángel Martín Pendás: ampendas@uniovi.es;

Contents

1 NNAIMQ database **3**

2 Chemical reactions **12**
2.1 Reconstruction of the molecular charge . . . 12
2.2 Diels-Alder reaction between 1,3-butadiene and acetylene . . . 13
2.3 Evolution of the atomic charges . . . 14

3 Molecular Dynamics simulations **16**
3.1 Cyclopamine molecule . . . 16
3.2 Steroid supramolecular complex . . . 20
3.3 Reconstructed molecular charge . . . 26

4 Large systems **27**

5 Further algorithmic details **39**
5.1 Non-tunable hyper-parameters . . . 39
5.2 Weight distribution schemes . . . 39
5.3 Performance metrics . . . 42
5.4 NNAIMQ dataset . . . 42
5.5 Main work-flow . . . 43
5.5.1 Command-line execution . . . 44
5.5.2 GUI-interfaced execution . . . 46
5.6 Using custom models in NNAIMGUI . . . 49
5.6.1 Loading FFNN models . . . 49
5.6.2 Loading tailor-made charge equilibration schemes . . . 53
5.7 Training models in NNAIMGUI . . . 53

1 NNAIMQ database

The following figures collect the dispersion plots for the predicted QTAIM atomic charges of C, H, O and N atoms of the NNAIMQ testing database, as corrected by different charge equilibration schemes.

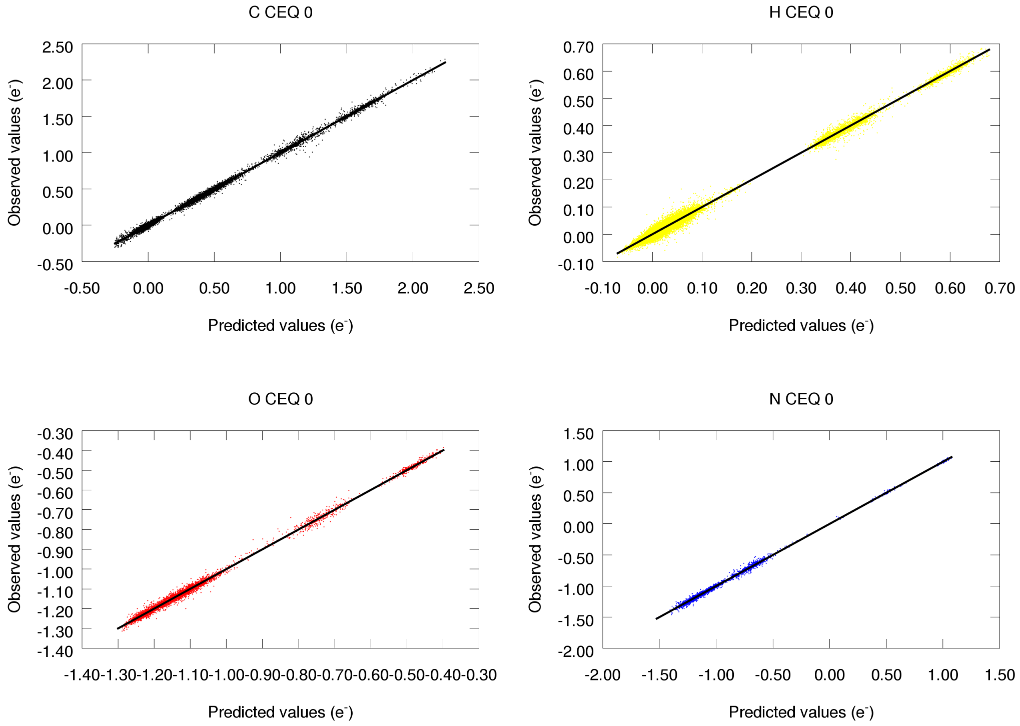

Figure S1: Dispersion plots for the charge equilibration scheme 0.

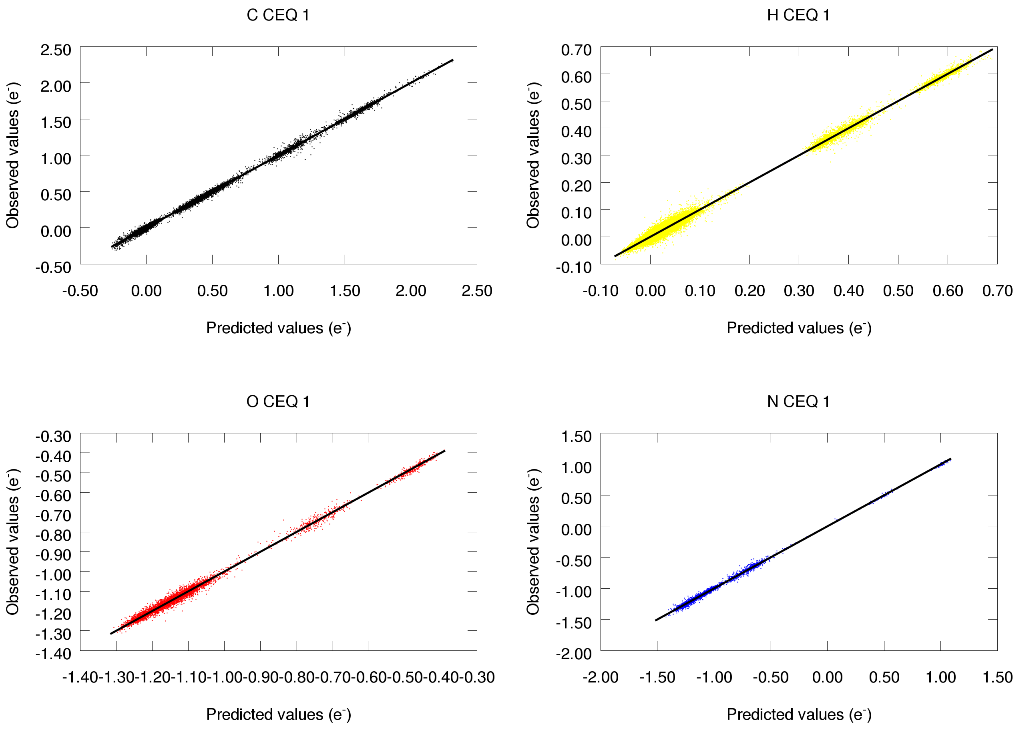

Figure S2: Dispersion plots for the charge equilibration scheme 1.

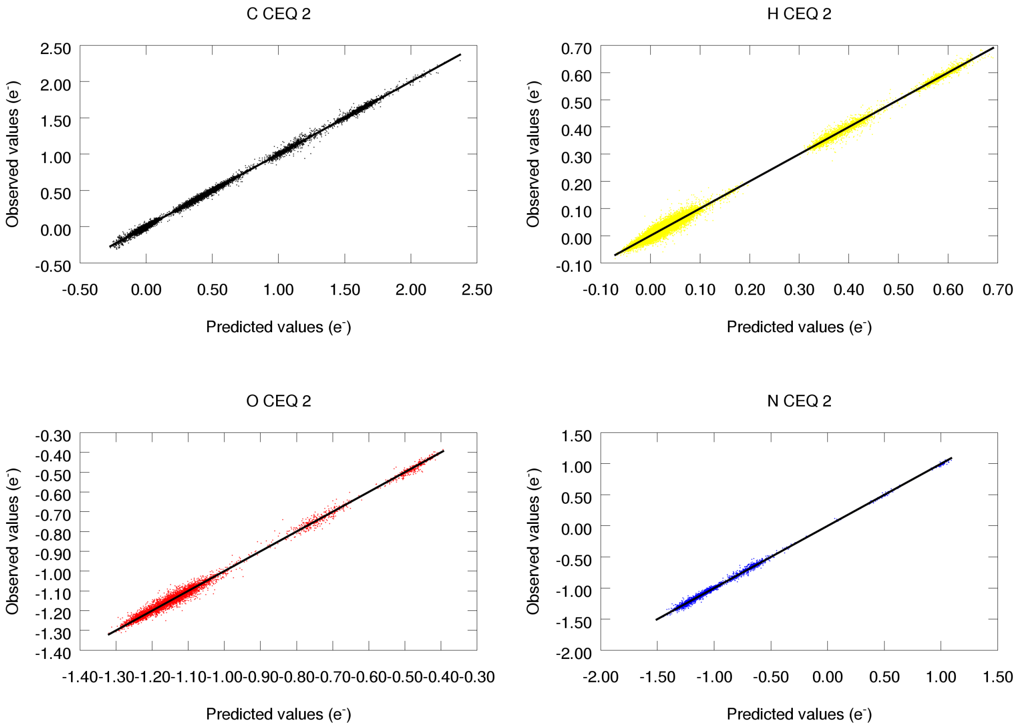

Figure S3: Dispersion plots for the charge equilibration scheme 2.

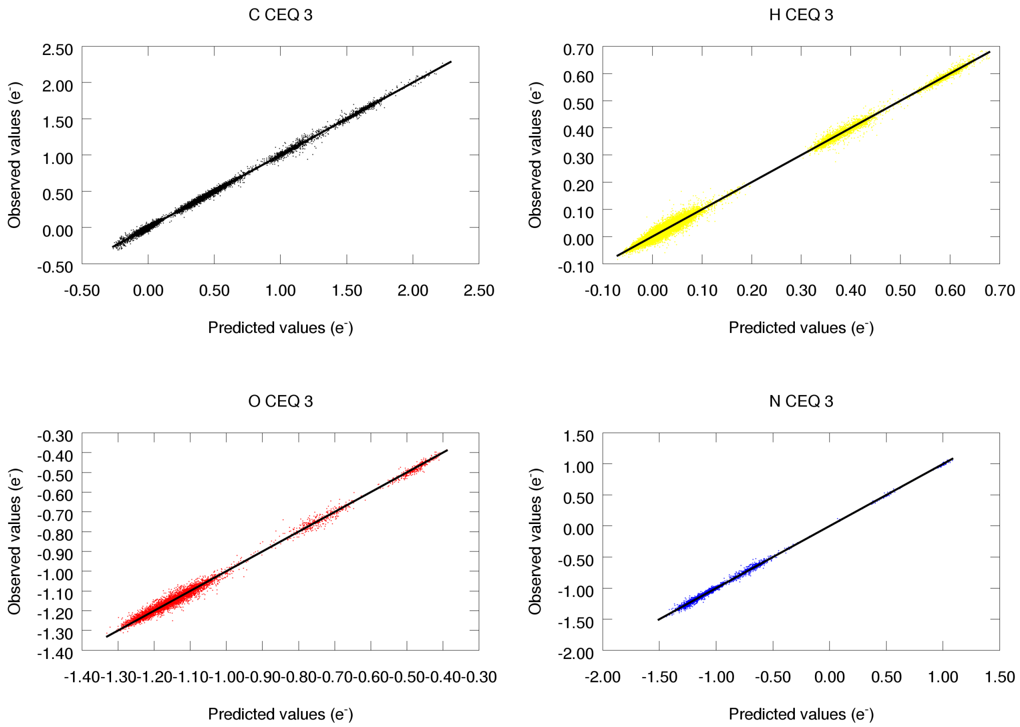

Figure S4: Dispersion plots for the charge equilibration scheme 3.

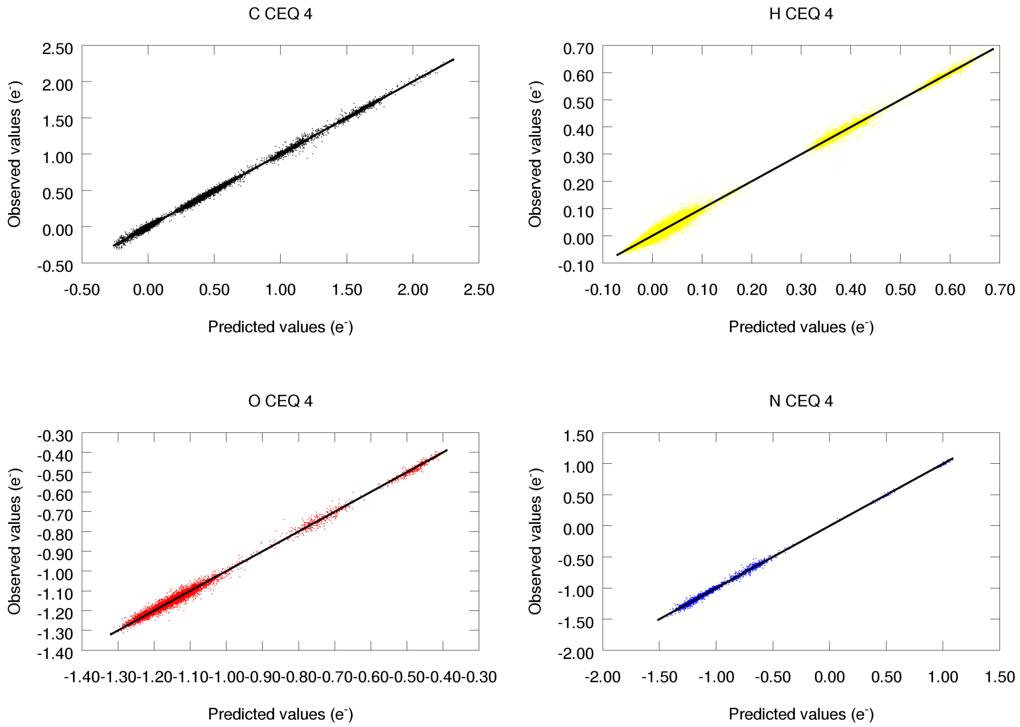

Figure S5: Dispersion plots for the charge equilibration scheme 4.

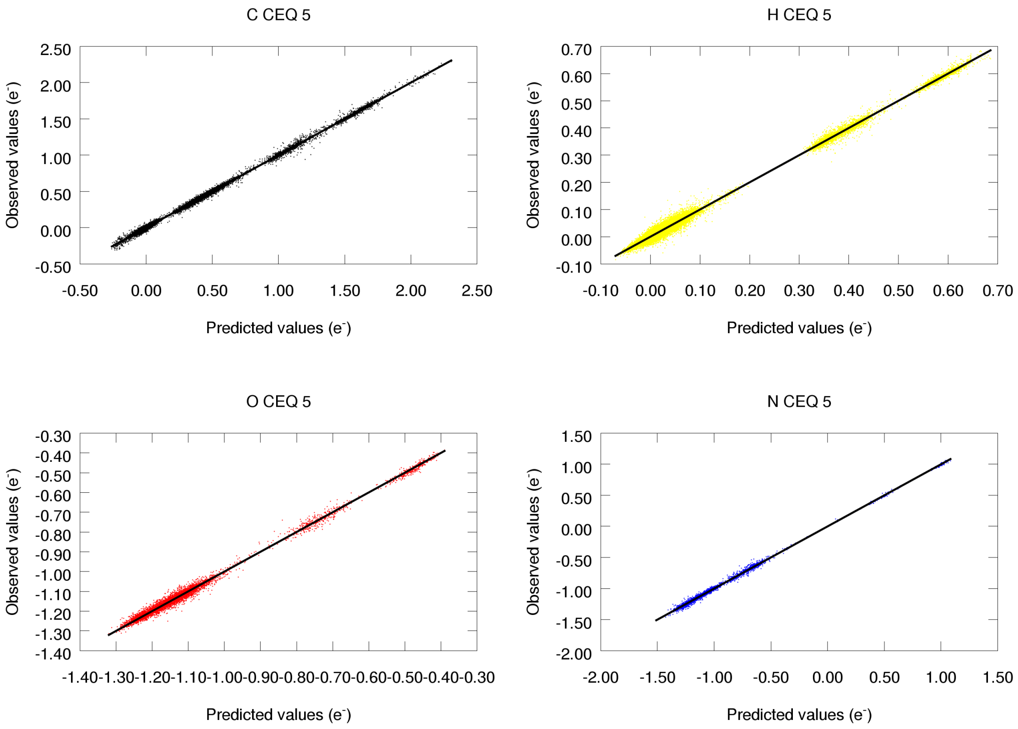

Figure S6: Dispersion plots for the charge equilibration scheme 5.

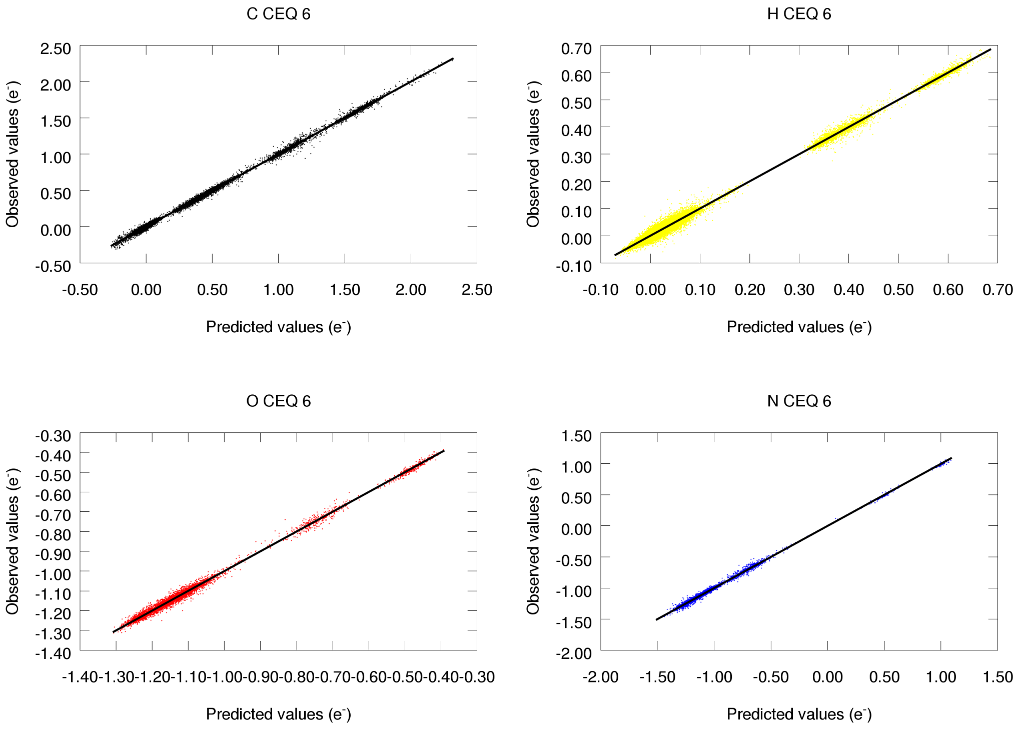

Figure S7: Dispersion plots for the charge equilibration scheme 6.

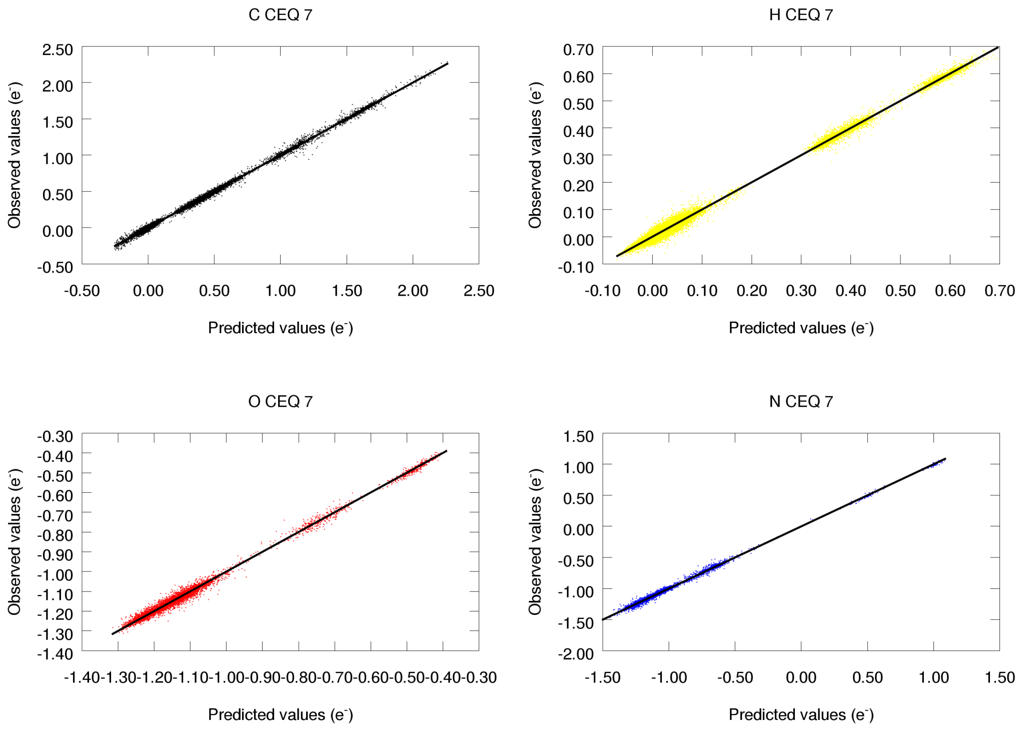

Figure S8: Dispersion plots for the charge equilibration scheme 7.

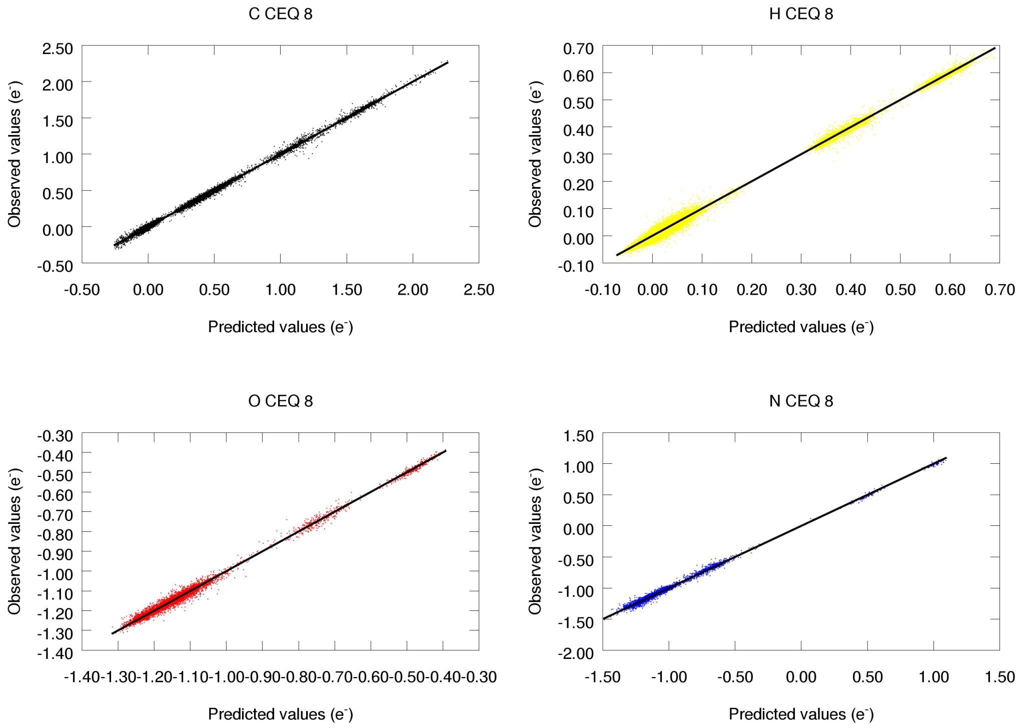

Figure S9: Dispersion plots for the charge equilibration scheme 8.

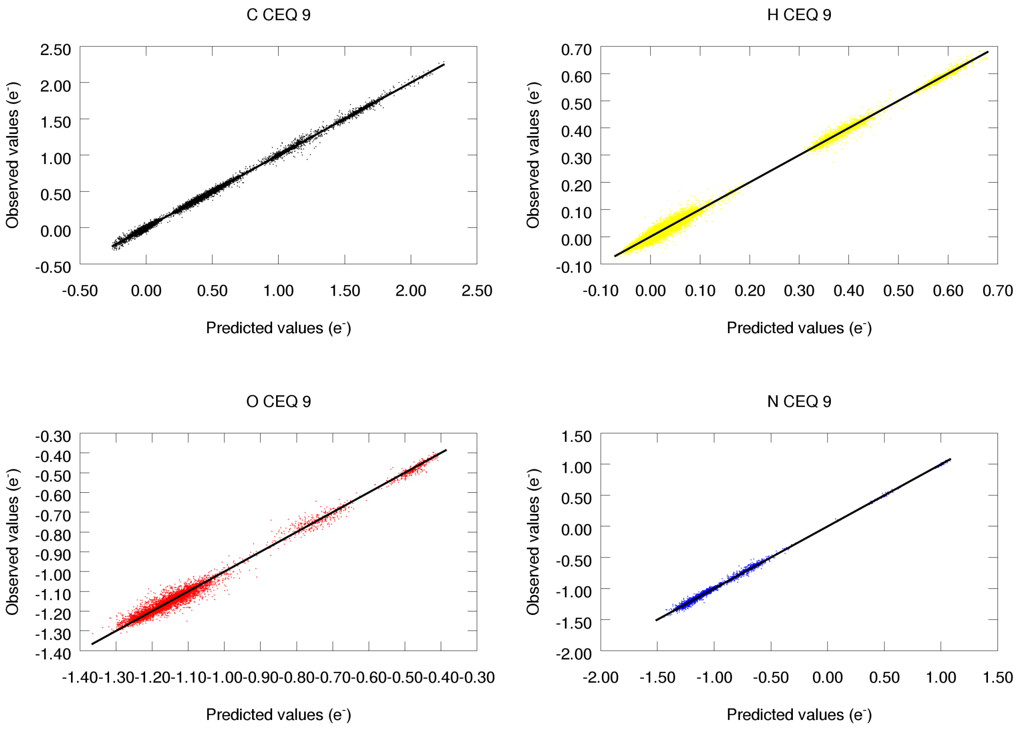

Figure S10: Dispersion plots for the charge equilibration scheme 9.

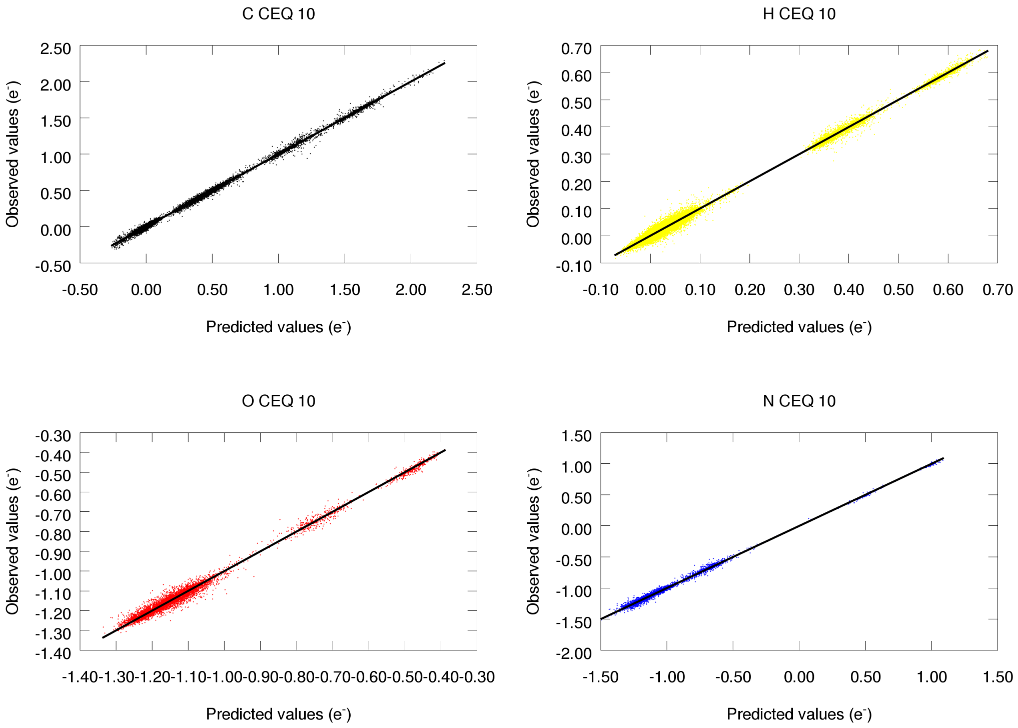

Figure S11: Dispersion plots for the charge equilibration scheme 10.

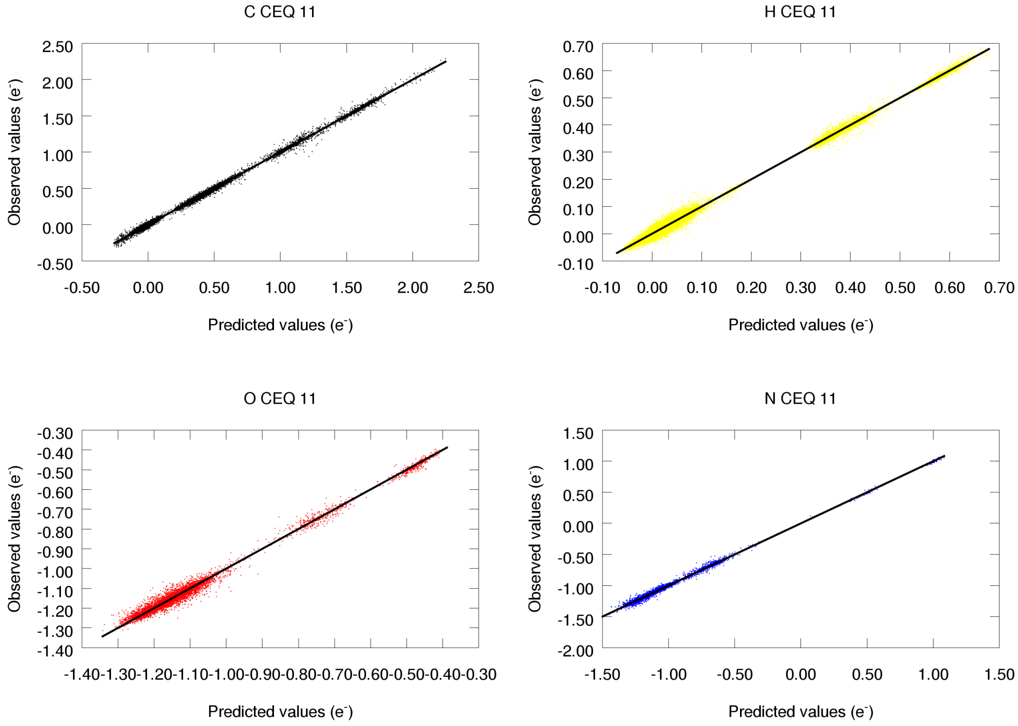

Figure S12: Dispersion plots for the charge equilibration scheme 11.

The following table gathers the errors, as given by the MAE and RMSE metrics, committed by the NNAIMQ model (in combination with different charge equilibration schemes) in the prediction of the atomic charges.

Table S1: Atomic Mean Absolute Errors (MAE) of the NNAIMQ predictions with different charge equilibration schemes (CEQ). All values are given in electrons.

CEQ	C	H	O	N
–	0.010	0.006	0.008	0.016
1	0.010	0.006	0.010	0.017
2	0.010	0.006	0.012	0.019
3	0.010	0.006	0.013	0.018
4	0.010	0.006	0.011	0.017
5	0.010	0.006	0.011	0.017
6	0.010	0.006	0.010	0.018
7	0.010	0.007	0.012	0.019
8	0.010	0.007	0.011	0.022
9	0.010	0.006	0.017	0.019
10	0.010	0.006	0.014	0.021
11	0.010	0.006	0.016	0.021

Table S2: Atomic Root Mean Squared Errors (RMSE) of the NNAIMQ predictions with different charge equilibration schemes (CEQ). All values are given in electrons.

CEQ	C	H	O	N
–	0.015	0.009	0.011	0.022
1	0.015	0.009	0.014	0.023
2	0.015	0.009	0.016	0.025
3	0.015	0.009	0.017	0.024
4	0.015	0.009	0.015	0.023
5	0.015	0.009	0.015	0.023
6	0.015	0.009	0.013	0.025
7	0.015	0.009	0.016	0.026
8	0.015	0.009	0.015	0.029
9	0.015	0.009	0.023	0.025
10	0.015	0.009	0.020	0.029
11	0.015	0.009	0.021	0.028

The following figures gather the distribution of the observed values of the atomic charges of the C, H, O and N atoms of the NNAIMQ external validation (testing) dataset.

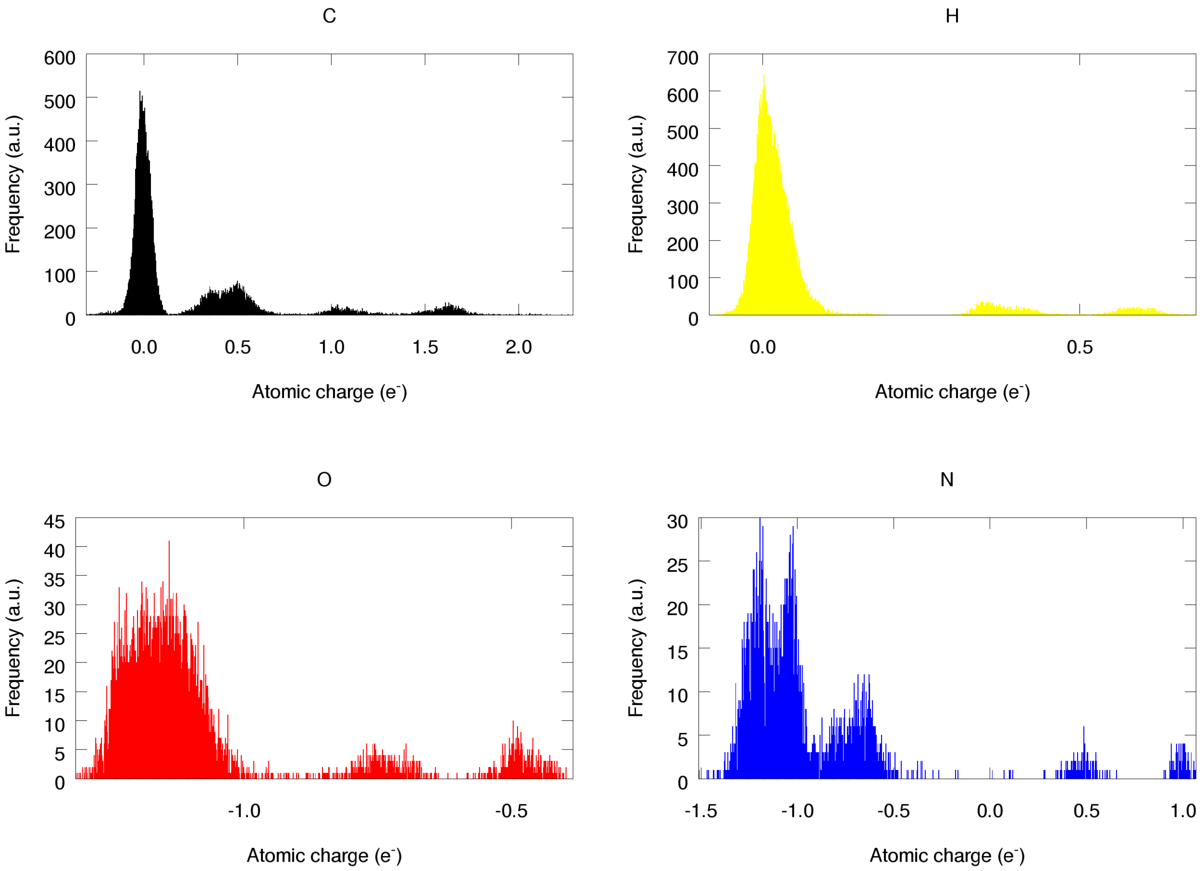

Figure S13: Distribution of the quantum chemically computed atomic charges within the NNAIMQ external validation dataset.

2 Chemical reactions

2.1 Reconstruction of the molecular charge

The following figures show the evolution of the excess of molecular charge arising from the uncorrected NNAIMQ predictions throughout the different chemical transformations under study.

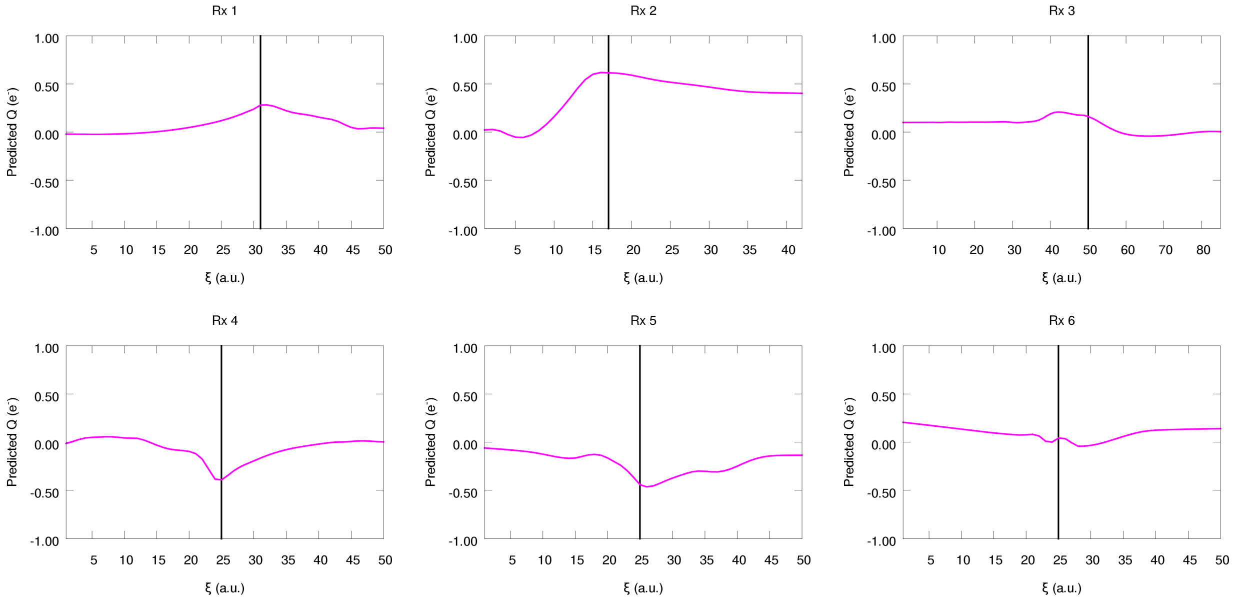

Figure S14: Evolution of the NNAIMQ predicted molecular charge throughout the chemical reactions under study. For the sake of clarity, the relative position of the TS in the IRC path is highlighted as a vertical line.

2.2 Diels-Alder reaction between 1,3-butadiene and acetylene

The Diels-Alder cycloaddition reaction between 1,3-butadiene and acetylene was studied in the gas phase and at the DFT level of theory (M06-2X Minnesota functional in combination with the def2-TZVP basis set). All the calculations (geometry optimizations, single point calculations and normal mode analysis) were performed with the Gaussian09 quantum chemistry package[1]. The nature of the potential wells (reactants and products) and the transition state was characterized as local minimum and first order saddle points, respectively, throughout the analysis of the eigenvalues of the Hessian matrix. Similarly, the analysis of the QTAIM atomic charges was achieved thanks to the PROMOLDEN[2] code.

The following figure gathers the reaction energy profile of the aforementioned Diels-Alder reaction.

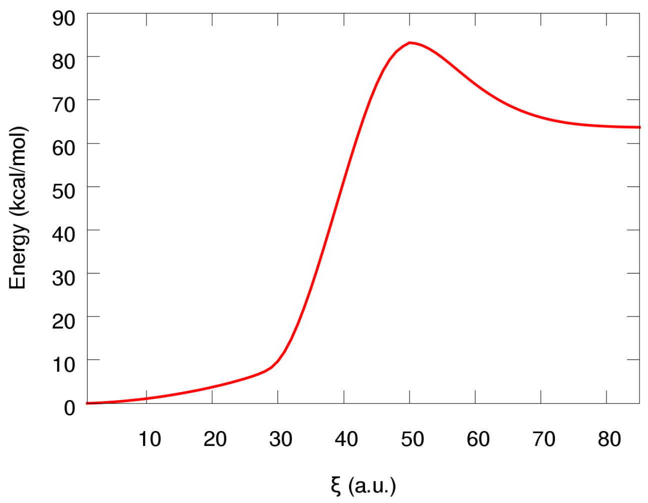

Figure S15: Reaction energy profile for the Diels-Alder reaction between 1,3-butadiene and acetylene. The electronic energy is reported relative to that of the starting reactant complex (-233.391 Hartrees).

On the other hand, the following table collects the optimized geometry of the transition state involved in the reaction.

Table S3: XYZ coordinates of the optimized transition state (TS) of the Diels-Alder reaction between 1,3-butadiene and acetylene. All values are reported in Å.

Atom	X	Y	Z
C	-0.358310	-1.420910	0.476580
C	-1.229170	-0.706160	-0.297390
H	-0.285380	-2.495110	0.358480
H	0.009290	-1.035730	1.418240
C	-1.232940	0.699770	-0.297810
H	-1.777080	-1.219430	-1.079040
C	-0.367080	1.419800	0.476660
H	-0.299250	2.494210	0.357880
H	0.004340	1.036360	1.417590
H	-1.783760	1.209760	-1.079560
C	1.601760	0.616980	-0.234530
C	1.604280	-0.609400	-0.234020
H	2.006710	1.595210	-0.365020
H	2.013930	-1.585620	-0.365450

2.3 Evolution of the atomic charges

The current section gathers the evolution of the equilibrated atomic charges along the different chemical transformation under study.

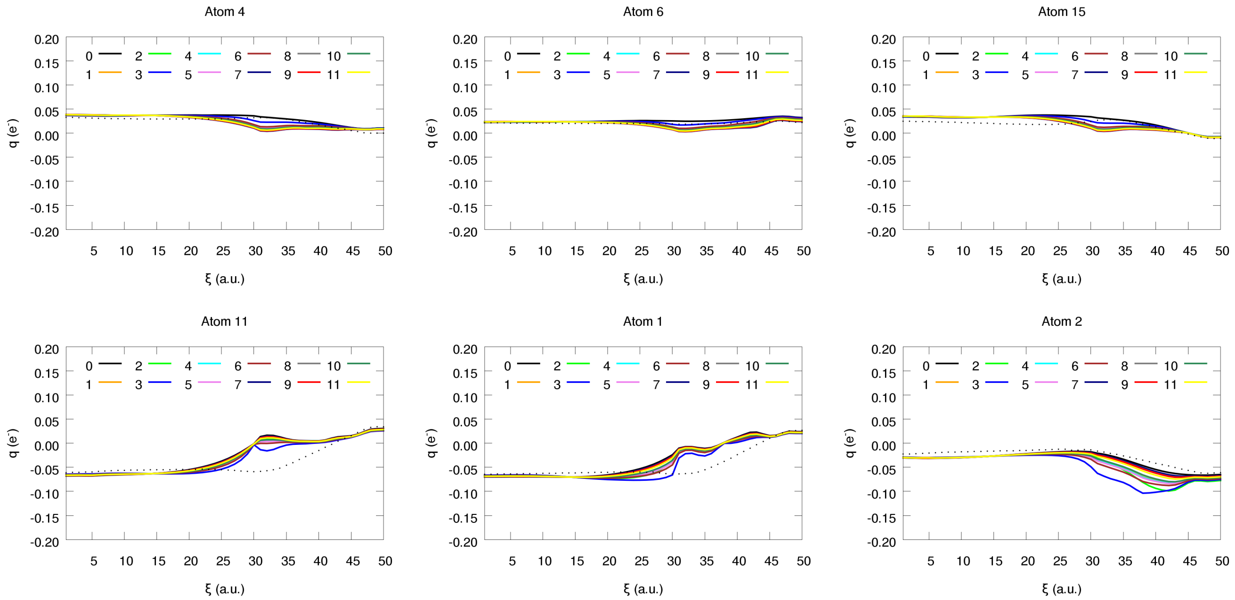

Figure S16: Evolution of the NNAIMQ equilibrated atomic charges along the Diels-Alder cycloaddition between 1,3-butadiene and ethylene (Rx 1).

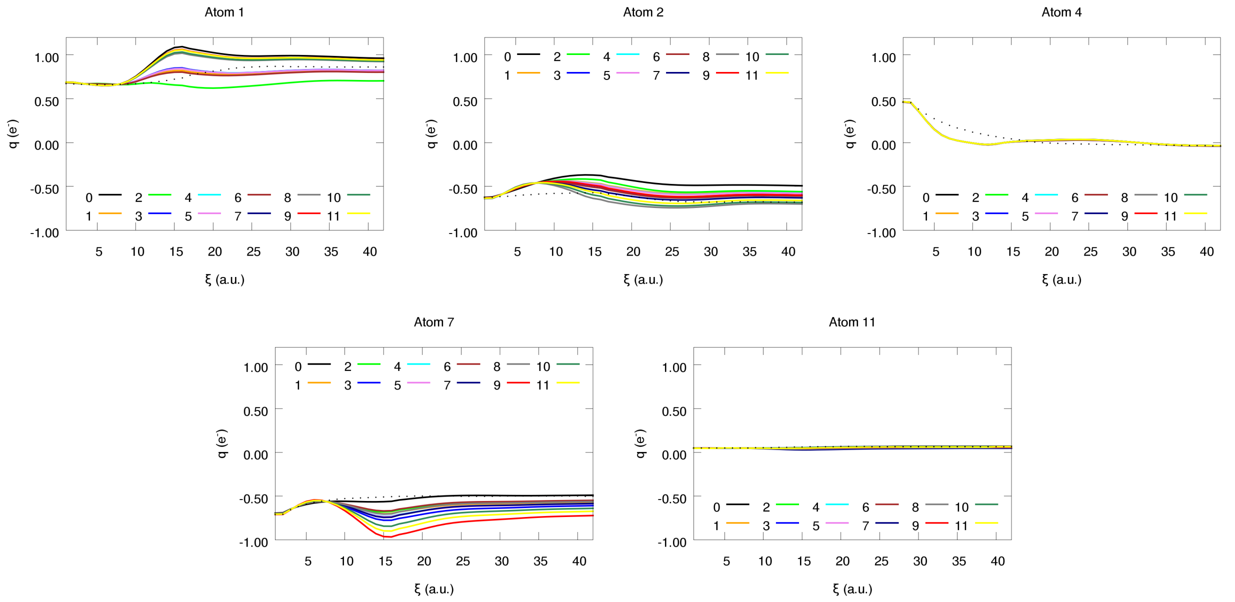

Figure S17: Evolution of the NNAIMQ equilibrated atomic charges along the 1,3-dipolar cycloaddition between acetonitrile oxide and ethylene (Rx 2).

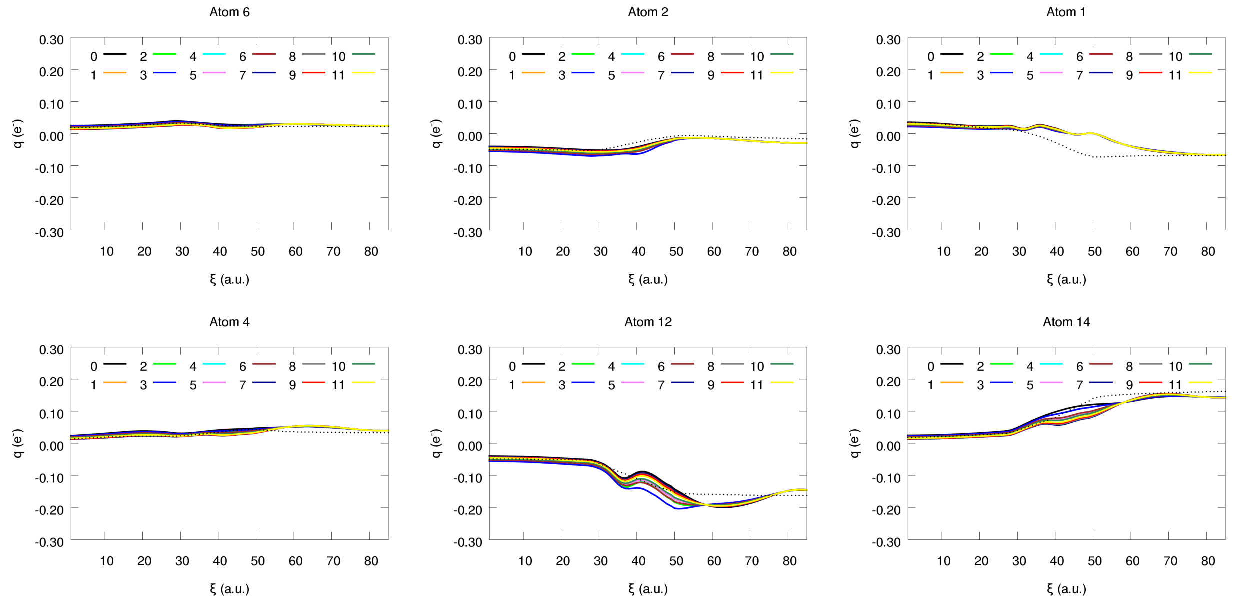

Figure S18: Evolution of the NNAIMQ equilibrated atomic charges along the Diels-Alder cycloaddition between 1,3-butadiene and acetylene (Rx 3).

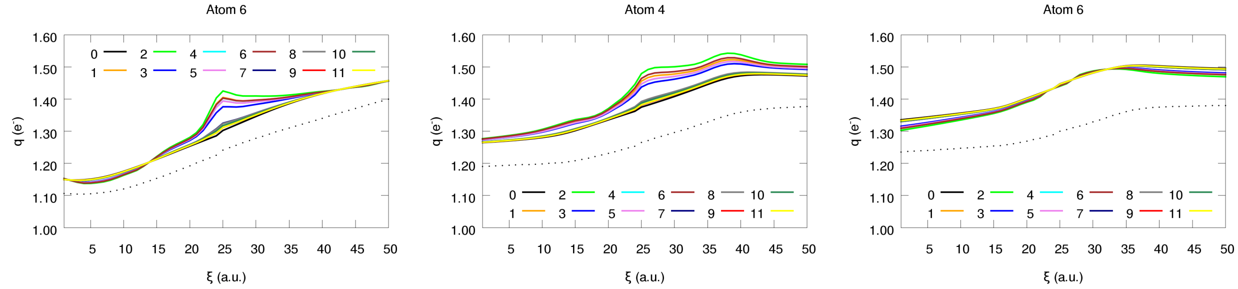

Figure S19: Evolution of the NNAIMQ equilibrated atomic charges of the C atom along the tautomerism reactions of 2-hydroxypyridine (Rx 4, 5 and 6, respectively).

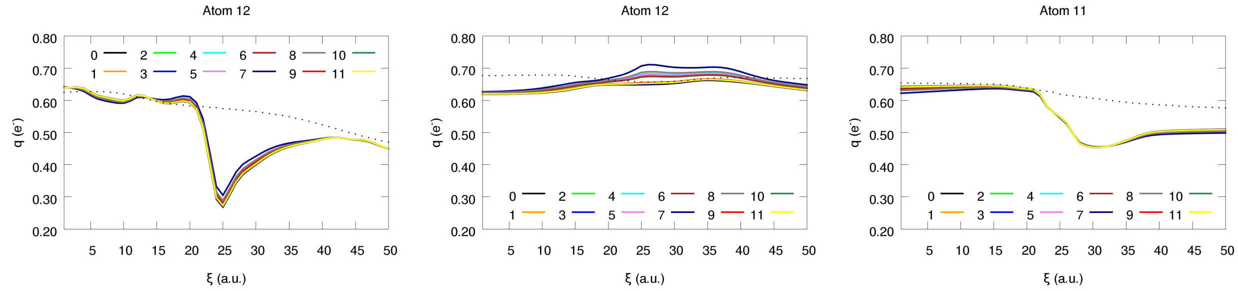

Figure S20: Evolution of the NNAIMQ equilibrated atomic charges of the H atom along the tautomerism reactions of 2-hydroxypyridine (Rx 4, 5 and 6, respectively).

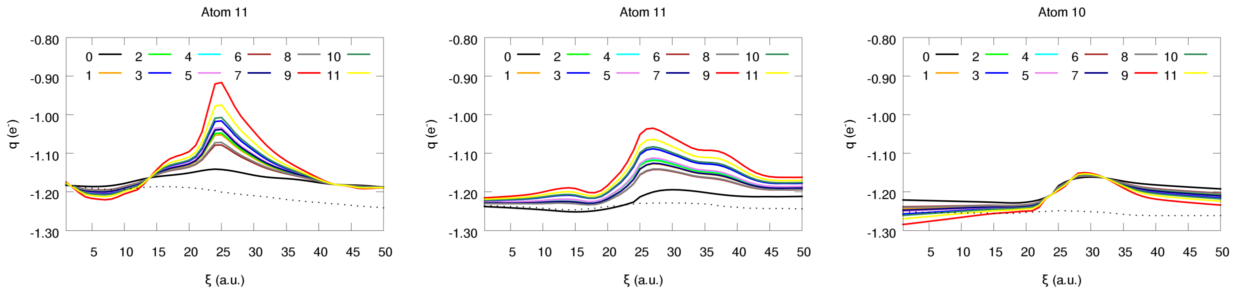

Figure S21: Evolution of the NNAIMQ equilibrated atomic charges of the O atom along the tautomerism reactions of 2-hydroxypyridine (Rx 4, 5 and 6, respectively).

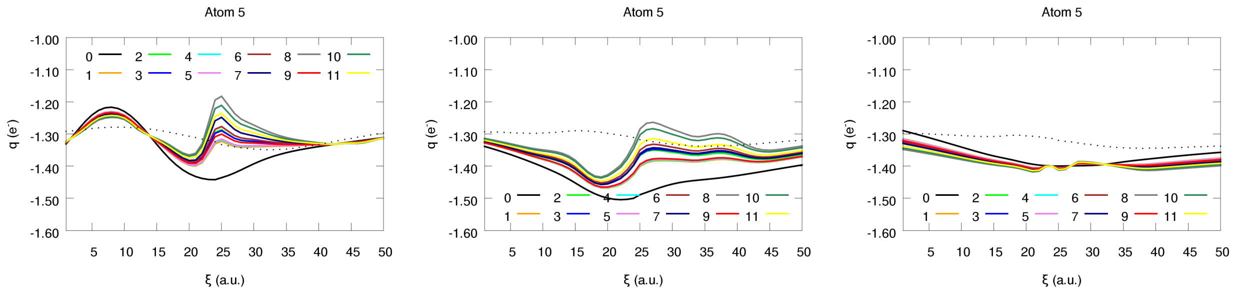

Figure S22: Evolution of the NNAIMQ equilibrated atomic charges of the N atom along the tautomerism reactions of 2-hydroxypyridine (Rx 4, 5 and 6, respectively).

3 Molecular Dynamics simulations

3.1 Cyclopamine molecule

The following table gathers the error committed by the different charge equilibration schemes in the prediction of the atomic charges of the Cyclopamine molecule, computed with respect to the quantum chemically estimated values.

Table S4: Global errors, as given by the L1 (MAE) and L2 (RMSE) norms, and Pearson correlation coefficient (R) for the NNAIMQ predictions with different charge equilibration schemes.

CEQ	MAE (e^-)	RMSE (e^-)	R (a.u.)	R^2 (a.u.)
0	0.0095	0.0127	0.9989	0.9979
1	0.0097	0.0129	0.9988	0.9976
2	0.0101	0.0143	0.9987	0.9975
3	0.0099	0.0138	0.9988	0.9975
4	0.0098	0.0129	0.9988	0.9976
5	0.0098	0.0131	0.9988	0.9976
6	0.0096	0.0127	0.9988	0.9976
7	0.0099	0.0135	0.9988	0.9976
8	0.0099	0.0141	0.9988	0.9975
9	0.0104	0.0156	0.9986	0.9972
10	0.0102	0.0148	0.9988	0.9976
11	0.0103	0.0151	0.9988	0.9975

On the other hand, and for the sake of convenience, the remaining metrics will be reported using the non-equilibrated values (CEQ= 0) as ground-truth data. This provides a particularly suitable way of measuring the resemblance between the equilibrated and the raw ML predictions.

The following tables gather the L1 and L2 error metrics along with the Pearson correlation coefficient (as R^2) for the different charge equilibration schemes when applied throughout the MD simulations of the Cyclopamine molecule. All metrics have been computed with respect to the uncorrected values.

Table S5: Mean Absolute Errors (MAE) for the NNAIMQ equilibrated atomic charges of the Cyclopamine molecule as corrected by different charge equilibration schemes. All values are reported in electrons.

CEQ	C	H	O	N
1	0.002	0.001	0.025	0.023
2	0.001	0.000	0.042	0.036
3	0.002	0.000	0.038	0.031
4	0.002	0.001	0.030	0.024
5	0.002	0.001	0.031	0.025
6	0.003	0.001	0.020	0.037
7	0.001	0.001	0.030	0.040
8	0.001	0.001	0.025	0.066
9	0.000	0.000	0.064	0.027
10	0.001	0.000	0.047	0.056
11	0.000	0.000	0.055	0.046

Table S6: Root Mean Squared Errors (RMSE) for the NNAIMQ equilibrated atomic charges of the Cyclopamine molecule as corrected by different charge equilibration schemes. All values are reported in electrons.

CEQ	C	H	O	N
1	0.005	0.003	0.027	0.025
2	0.003	0.002	0.045	0.039
3	0.004	0.000	0.040	0.033
4	0.004	0.002	0.032	0.026
5	0.004	0.002	0.033	0.027
6	0.005	0.002	0.021	0.040
7	0.001	0.005	0.032	0.043
8	0.001	0.003	0.027	0.071
9	0.001	0.001	0.068	0.029
10	0.001	0.000	0.051	0.060
11	0.001	0.000	0.059	0.050

Table S7: Pearson correlation coefficient between the equilibrated and raw atomic charges of the Cyclopamine molecule for different electron redistribution approaches.

CEQ	C	H	O	N
1	1.000	1.000	0.960	0.951
2	1.000	1.000	0.899	0.901
3	1.000	1.000	0.914	0.921
4	1.000	1.000	0.943	0.947
5	1.000	1.000	0.941	0.944
6	1.000	1.000	0.974	0.894
7	1.000	1.000	0.944	0.880
8	1.000	1.000	0.960	0.757
9	1.000	1.000	0.789	0.936
10	1.000	1.000	0.871	0.807
11	1.000	1.000	0.834	0.851

The following figure shows the evolution of the atomic charges of a collection of relevant atoms of the Cyclopamine molecule during the progress of the MD simulation.

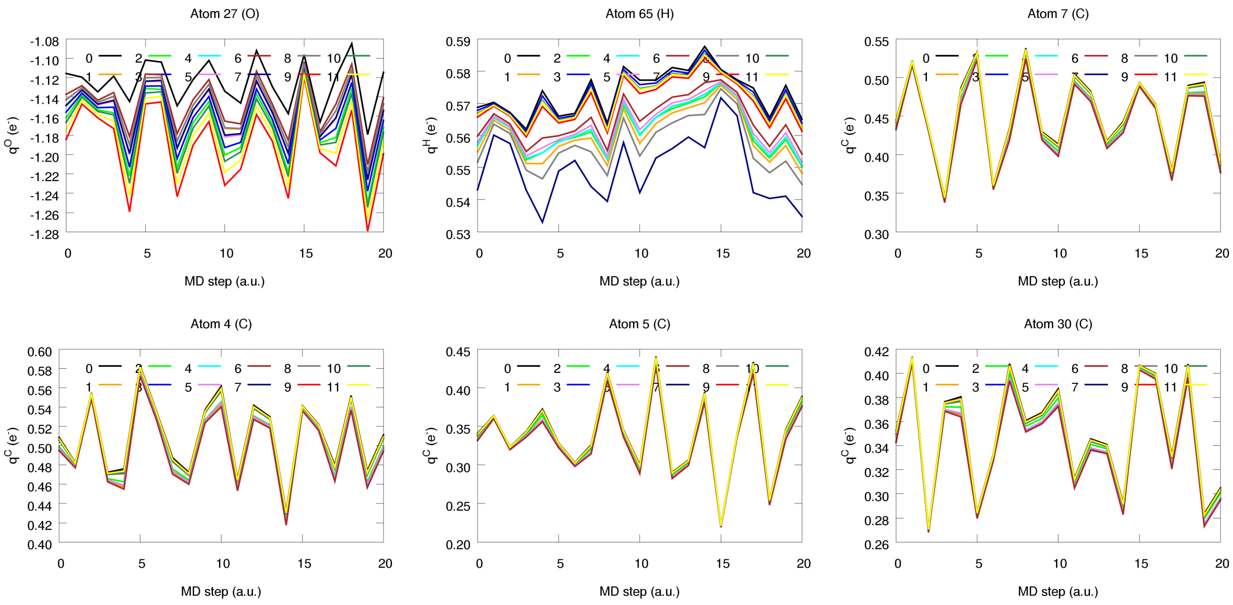

Figure S23: Evolution of the NNAIMQ equilibrated partial charges of some of the atoms of the Cyclopamine molecule throughout the MD simulation.

The following table collect the Cartesian coordinates of the Cyclopamine molecule, given in Å.

Atom	X	Y	Z
C	0.393039	-1.676930	3.138768
C	1.775483	-1.054063	3.066369
C	2.759408	-1.958722	2.338691
C	4.188841	-1.465245	2.381729
C	4.648608	-0.909614	3.728524
C	5.849428	-0.035118	3.338435
C	5.674909	0.213786	1.792475
O	4.380357	-0.381032	1.478913
C	6.741082	-0.498940	0.958802
C	6.727151	-0.059843	-0.491466
C	7.083969	1.406205	-0.538091
C	6.928662	2.112756	-1.884122
C	8.030501	1.799730	-2.904478
C	8.544496	2.999294	-3.630944
C	8.570745	4.218166	-3.094081
C	9.060809	5.425699	-3.815719
C	7.885168	6.371756	-4.084809
C	7.006052	6.525845	-2.826810
C	7.651861	5.960877	-1.571349
C	8.015688	4.467745	-1.703602
C	6.743866	3.606986	-1.541118
C	6.111178	3.608944	-0.136063
C	6.214384	2.200683	0.390157
C	5.549043	1.683477	1.427349
C	4.564090	2.495427	2.209550
C	9.104550	4.128833	-0.681417
O	7.126721	5.922176	-5.207404
C	7.171691	-0.630101	3.789939
N	3.646985	-0.116780	4.434255
C	2.330111	-0.737838	4.452542
H	0.001537	-1.894074	2.144889

H	-0.316628	-1.016111	3.637304
H	0.415392	-2.616354	3.694580
H	1.704463	-0.111096	2.515549
H	2.462722	-2.104992	1.298717
H	2.738378	-2.946363	2.809076
H	4.851745	-2.298022	2.102070
H	4.950268	-1.728556	4.387240
H	5.745729	0.913233	3.860500
H	6.594530	-1.575079	1.044008
H	7.729591	-0.290069	1.367271
H	5.732280	-0.209573	-0.915171
H	7.427670	-0.655392	-1.079750
H	8.129381	1.512966	-0.221397
H	5.977133	1.776768	-2.302711
H	7.684400	1.052924	-3.619285
H	8.870759	1.321781	-2.386859
H	8.964740	2.845003	-4.619993
H	9.533070	5.170767	-4.762761
H	9.807684	5.950327	-3.213714
H	8.274957	7.341075	-4.394995
H	6.064401	6.011960	-3.022386
H	6.741290	7.572428	-2.676956
H	6.991758	6.102075	-0.715092
H	8.553604	6.530682	-1.339492
H	6.027040	4.020405	-2.249102
H	5.071939	3.932130	-0.189661
H	6.602264	4.318736	0.531305
H	4.366201	3.454403	1.741223
H	4.900073	2.706326	3.226116
H	3.616282	1.962813	2.279657
H	9.977691	4.760037	-0.843708
H	9.430990	3.096155	-0.767160
H	8.767109	4.294020	0.341448
H	6.914714	4.992774	-5.042003
H	7.350082	-1.619254	3.370166
H	7.170389	-0.728996	4.874648
H	8.014833	0.005310	3.523963
H	3.570749	0.781286	3.975536
H	1.648928	-0.080627	4.996154
H	2.397566	-1.663860	5.033193

3.2 Steroid supramolecular complex

The following tables gather the L1 and L2 error metrics along with the Pearson correlation coefficient (as R^2) for the different charge equilibration schemes when applied throughout the MD simulations of the steroid supramolecular complex. All metrics have been computed with respect to the uncorrected values.

Table S8: Mean Absolute Errors (MAE) for the NNAIMQ equilibrated atomic charges of the steroid supramolecular complex as corrected by different charge equilibration schemes. All values are reported in electrons.

CEQ	C	H	O	N
1	0.007	0.002	0.031	0.027
2	0.009	0.001	0.033	0.026
3	0.006	0.000	0.047	0.036
4	0.007	0.002	0.036	0.028
5	0.007	0.002	0.037	0.029
6	0.008	0.001	0.025	0.045
7	0.002	0.004	0.039	0.049
8	0.002	0.003	0.034	0.085
9	0.001	0.001	0.070	0.028
10	0.001	0.000	0.058	0.064
11	0.001	0.000	0.064	0.051

Table S9: Root Mean Squared Errors (RMSE) for the NNAIMQ equilibrated atomic charges of the steroid supramolecular complex as corrected by different charge equilibration schemes. All values are reported in electrons.

CEQ	C	H	O	N
1	0.017	0.005	0.033	0.029
2	0.030	0.002	0.035	0.027
3	0.012	0.000	0.050	0.038
4	0.015	0.004	0.039	0.030
5	0.015	0.004	0.039	0.030
6	0.020	0.003	0.027	0.047
7	0.004	0.009	0.041	0.052
8	0.005	0.006	0.036	0.090
9	0.001	0.001	0.074	0.029
10	0.003	0.001	0.062	0.068
11	0.002	0.001	0.068	0.054

Table S10: Pearson correlation coefficient between the equilibrated and raw atomic charges of the steroid supramolecular complex for different electron redistribution approaches.

CEQ	C	H	O	N
1	1.000	1.000	0.960	0.992
2	0.999	1.000	0.955	0.993
3	1.000	1.000	0.911	0.986
4	1.000	1.000	0.945	0.992
5	1.000	1.000	0.943	0.991
6	1.000	1.000	0.972	0.979
7	1.000	0.999	0.937	0.975
8	1.000	1.000	0.950	0.925
9	1.000	1.000	0.815	0.992
10	1.000	1.000	0.865	0.957
11	1.000	1.000	0.839	0.972

The following figures show the evolution of the atomic charges of a collection of relevant atoms of the steroid supramolecular complex during the progress of the MD simulation. Given the large number of steps visited along the MD simulation, the average of every 15 steps is shown.

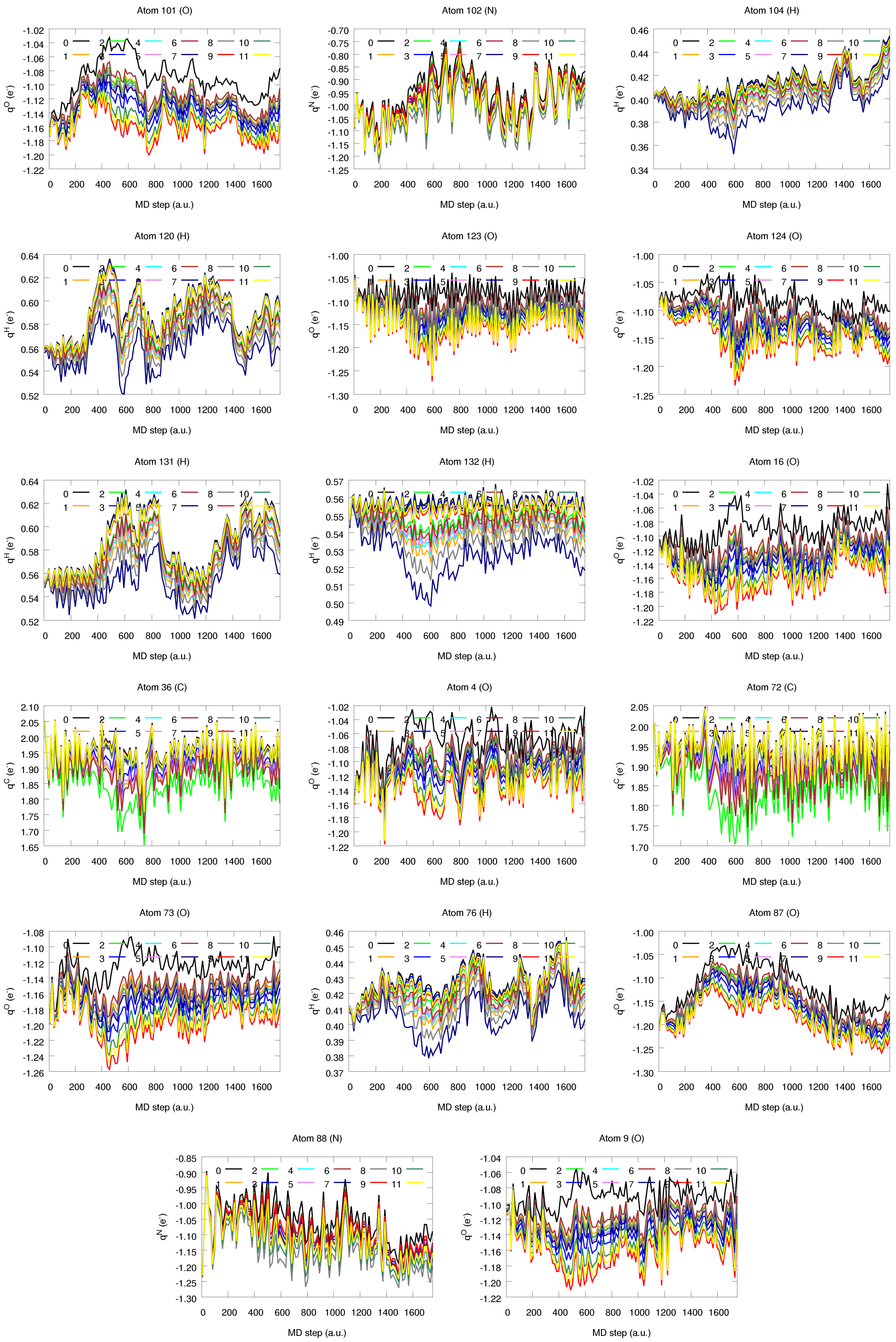

Figure S24: Evolution of the NNAIMQ equilibrated partial charges of some of the atoms of the steroid supramolecular complex throughout the MD simulation.

The following table collect the Cartesian coordinates of the steroid supramolecular complex, given in Å.

Atom	X	Y	Z
C	-10.348600	41.946010	54.698530
C	-9.419810	43.079900	55.098040
C	-7.986430	42.594280	55.340370
O	-7.265560	43.757170	55.760900
C	-8.006250	41.491370	56.405120
C	-8.940050	40.317210	56.030160
C	-8.889160	39.193190	57.087510
C	-9.649210	39.486380	58.395880
O	-8.927950	40.466760	59.156530
C	-11.102350	39.947830	58.078410
C	-11.144900	41.141630	57.069180
C	-10.404060	40.770140	55.716280
C	-11.142220	39.623100	54.967800
C	-12.596130	41.669840	56.857060
C	-13.413220	41.930930	58.158950
O	-12.852210	43.045680	58.871300
C	-13.414250	40.650170	59.049850
C	-14.191930	39.521560	58.314680
C	-11.921940	40.284660	59.335410
C	-12.039450	39.175760	60.370240
C	-13.194050	39.619120	61.263590
C	-13.942770	40.771740	60.529150
C	-15.480810	40.700990	60.823710
C	-16.239530	41.823180	60.110400
C	-15.759250	40.771960	62.356780
C	-17.193730	40.409350	62.818030
C	-17.619550	38.948430	62.694560
O	-18.856010	38.746580	62.844130
O	-16.558150	38.068350	62.567490
C	-16.957720	36.730320	62.310120
H	-9.998510	41.564820	53.728370
H	-11.349430	42.346190	54.503730
H	-9.414800	43.846310	54.312230
H	-9.802260	43.584280	55.992690
H	-7.575800	42.216570	54.394120
C	-5.895400	43.747880	55.672310
H	-8.292850	41.938070	57.358280
H	-6.992680	41.105540	56.560760
H	-8.518800	39.889310	55.107710
H	-7.840820	38.958740	57.303420
H	-9.310330	38.281680	56.644910
H	-9.713370	38.541160	58.953510
C	-8.220030	39.919210	60.213730
H	-11.584790	39.084580	57.608720
H	-10.594580	41.969510	57.534740
H	-10.581000	39.316760	54.076390
H	-12.131000	39.939370	54.619870
H	-11.277280	38.724910	55.573430
H	-13.161230	40.972740	56.232160
H	-12.544600	42.603610	56.287870
H	-14.444640	42.163700	57.871400
H	-14.298130	38.618800	58.924300
H	-13.718070	39.211910	57.380310
H	-15.203700	39.851370	58.056730
H	-11.435590	41.140300	59.828380

H	-11.133480	39.033330	60.957030
H	-12.265590	38.211880	59.899600
H	-12.811680	39.977720	62.226380
H	-13.842260	38.758880	61.464610
H	-13.570110	41.717430	60.948560
H	-15.864840	39.742520	60.456150
H	-16.191520	41.712360	59.026060
H	-15.833410	42.804380	60.376970
H	-17.302390	41.819440	60.366340
H	-15.065560	40.128900	62.908790
H	-15.542110	41.793160	62.698600
H	-17.252820	40.675650	63.884040
H	-17.910800	41.062630	62.306380
H	-17.548480	36.659990	61.390150
H	-17.505750	36.309070	63.159940
H	-16.052070	36.131880	62.170110
C	-13.191690	44.265780	58.313320
O	-14.271550	44.896010	58.377350
N	-12.097440	44.954210	57.853160
C	-11.820600	46.140560	57.176120
H	-11.271950	44.403420	58.060760
C	-10.523200	46.463440	56.925800
C	-12.744910	47.021640	56.721120
C	-12.408750	48.150650	56.065150
C	-11.116670	48.435220	55.836920
C	-10.170620	47.583750	56.271750
H	-9.118130	47.783500	56.106330
H	-9.729390	45.799240	57.262630
H	-13.795530	46.811710	56.893390
H	-13.192370	48.819610	55.729840
H	-10.834320	49.338920	55.311130
O	-5.041310	42.835650	55.618100
N	-5.279060	44.927330	55.959010
C	-5.675460	46.234890	55.737510
H	-4.367840	44.771560	56.365570
C	-6.382700	46.597620	54.639190
C	-5.336700	47.222680	56.600680
C	-5.697170	48.503640	56.389440
C	-6.389370	48.834450	55.284130
C	-6.729930	47.876490	54.403630
H	-7.296770	48.118470	53.511580
H	-6.665060	45.822170	53.931240
H	-4.793710	46.960360	57.504440
H	-5.437800	49.260280	57.121430
H	-6.686890	49.862530	55.111780
O	-8.612330	39.664430	61.374330
N	-6.906700	39.607110	59.949600
C	-5.828080	39.247410	60.737110
H	-6.686060	39.781020	58.979940
C	-4.821880	38.372560	60.138470
C	-3.755800	37.967810	60.852590
C	-3.606380	38.402200	62.118840
C	-4.508480	39.229460	62.682490
C	-5.598560	39.642180	62.008260
H	-4.989130	38.034910	59.123020
H	-3.030200	37.298040	60.407950
H	-2.747700	38.082560	62.698560

H	-4.373560	39.574860	63.701850
H	-6.318350	40.305760	62.481430
C	-7.813970	45.438680	62.028530
O	-7.075280	46.478710	61.399690
H	-7.164930	44.575610	62.190490
H	-8.665170	45.164790	61.401930
H	-8.182340	45.800780	62.991330
H	-6.782680	46.113240	60.532810
C	-11.862220	47.531570	63.807400
C	-3.967300	44.281610	61.732930
O	-13.069060	48.153970	63.394920
O	-3.015330	44.384130	60.682060
H	-3.441520	44.322560	62.690140
H	-4.502110	43.332840	61.653110
H	-4.665390	45.118520	61.673300
H	-12.027680	46.462650	63.963500
H	-11.538100	47.983140	64.748430
H	-11.087070	47.692310	63.054720
H	-3.565650	44.285990	59.855250
H	-13.339530	47.622970	62.593410

3.3 Reconstructed molecular charge

The following figures show the evolution of the molecular charge throughout the MD simulations of the Cyclopamine and steroid-complex systems, as reconstructed by the NNAIMQ model. For the steroid, the average of every 15 steps is shown.

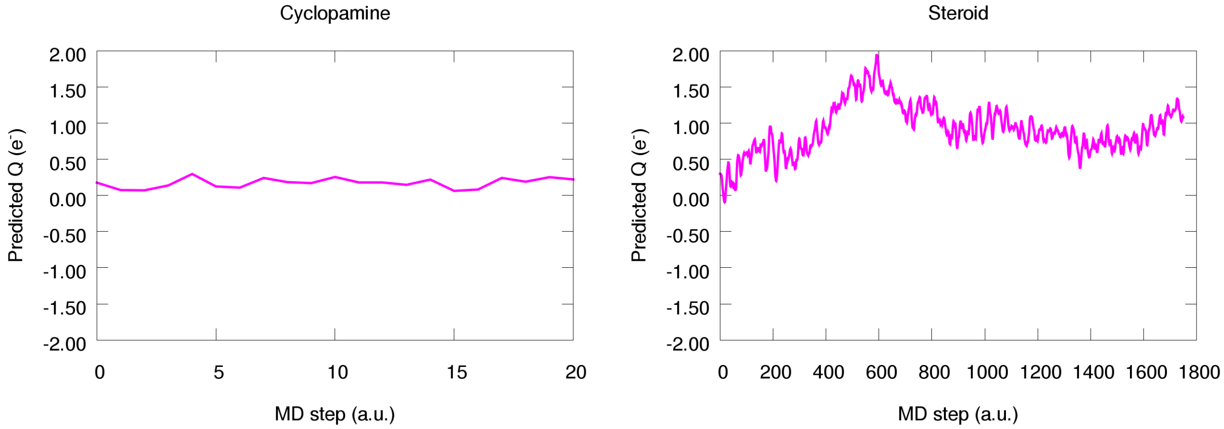

Figure S25: Evolution of the reconstructed molecular charge throughout the MD simulations of the Cyclopamine and steroid scaffolds.

4 Large systems

The following figures show the local partial charges, along with their respective errors, of the constituting atoms of the Chignolin and TC5b proteins, respectively, as reconstructed by the different charge equilibration schemes. All values will be given in electrons. On the other hand, the prediction errors are reported as the difference between the predicted and the quantum-chemically computed values.

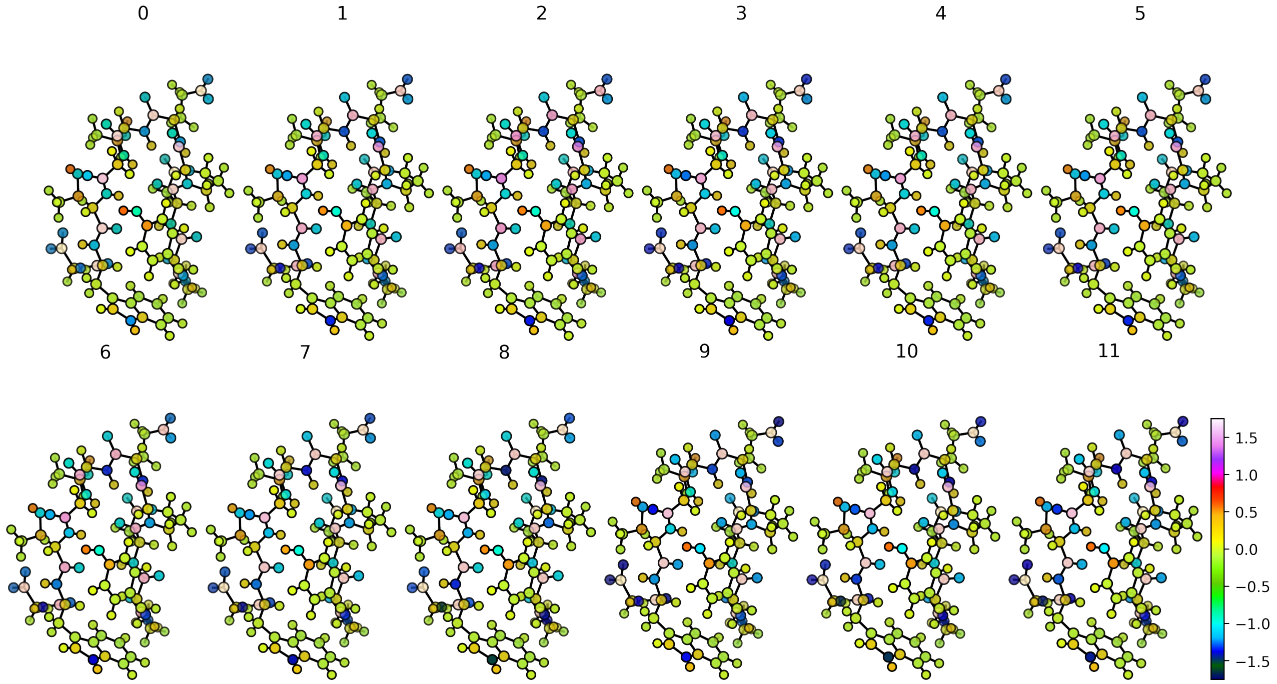

Figure S26: Atomic charges of the Chignolin protein as estimated by NNAIMQ in combination with different charge equilibration schemes. Images generated with the NNAIMGUI code.

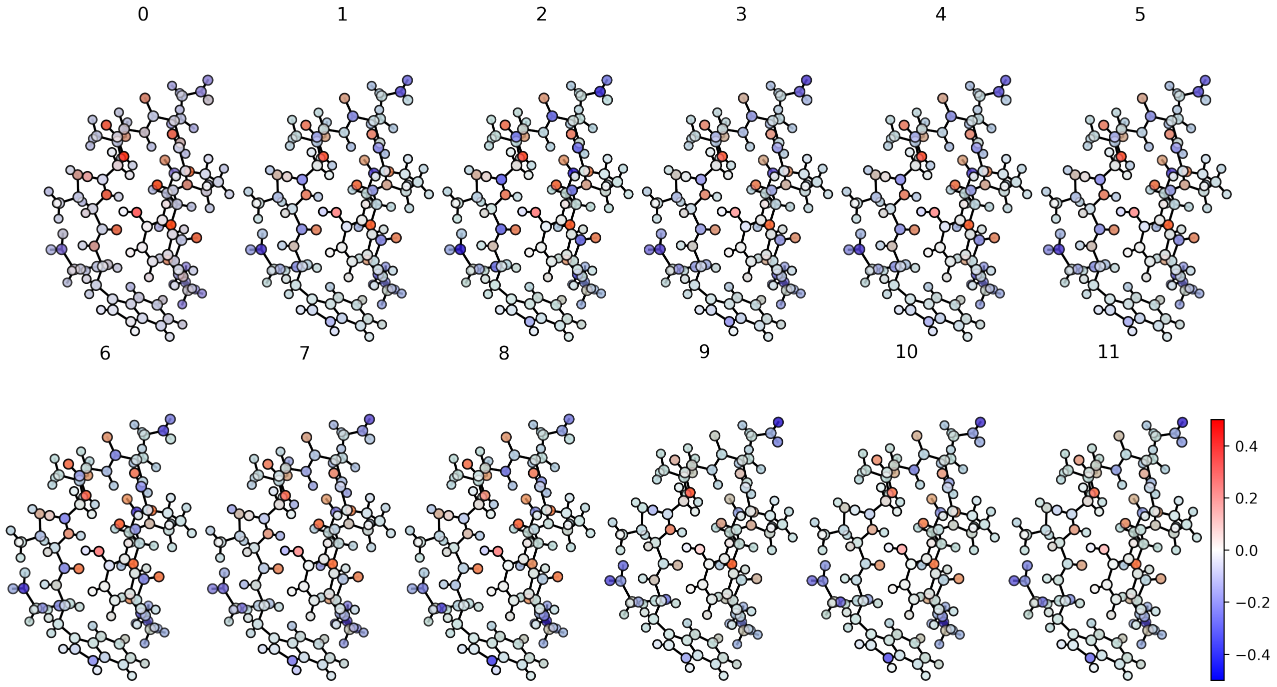

Figure S27: Errors in the predicted atomic charges of the Chignolin protein. Images generated with the NNAIMGUI code.

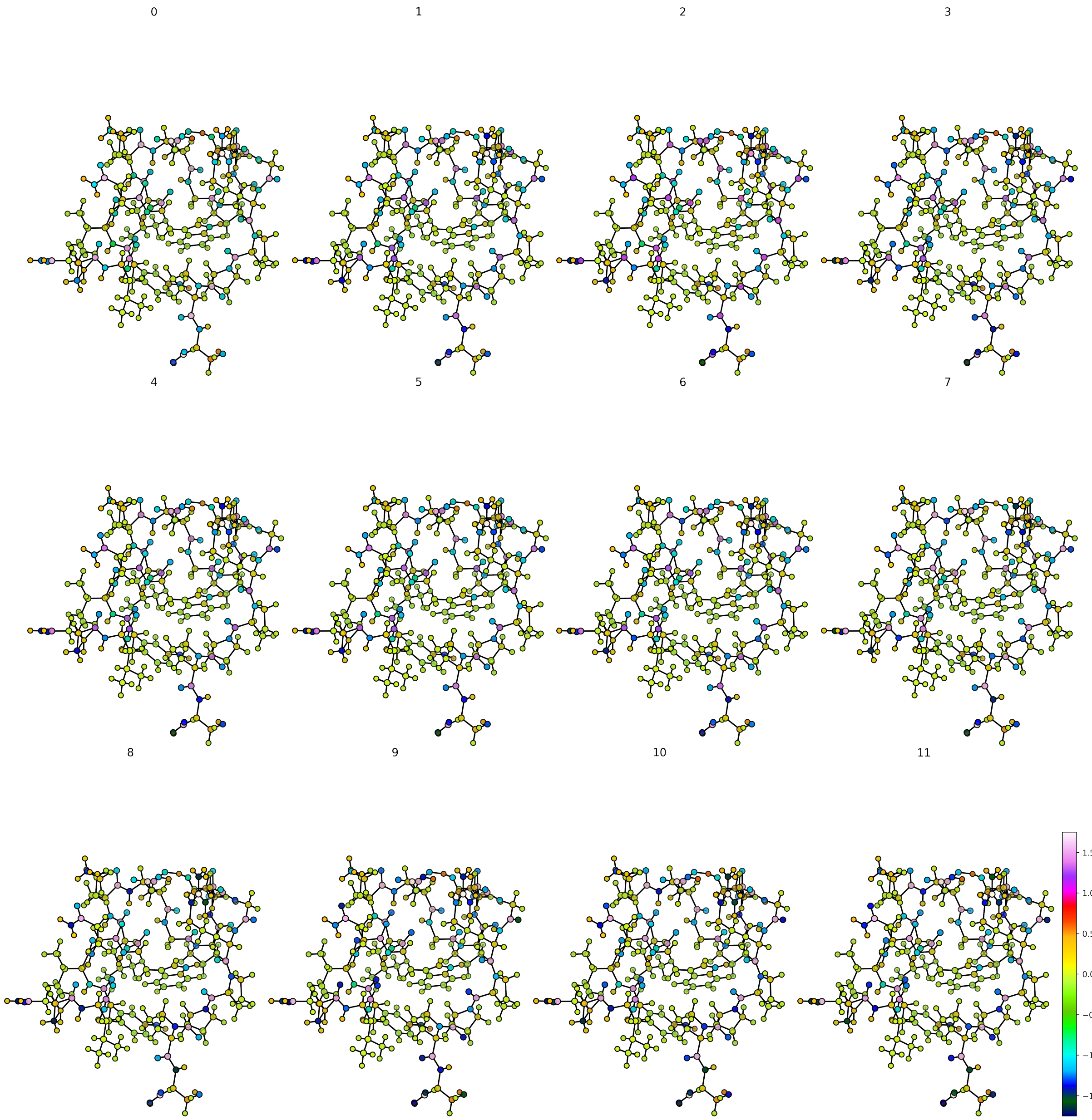

Figure S28: Atomic charges of the TC5b protein as estimated by NNAIMQ in combination with different charge equilibration schemes. Images generated with the NNAIMGUI code.

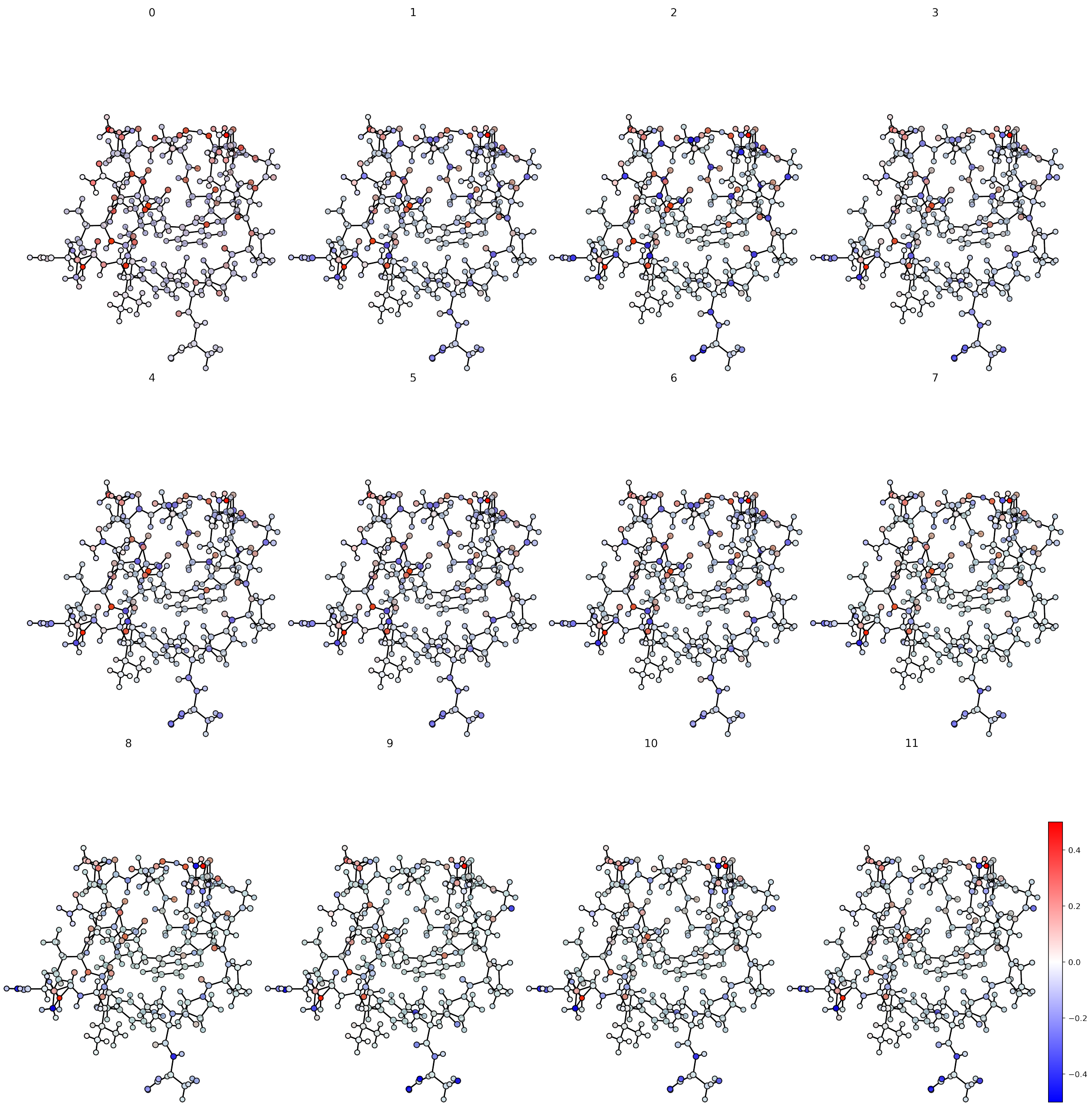

Figure S29: Errors in the predicted atomic charges of the TC5b protein. Images generated with the NNAIMGUI code.

The following tables collect the Cartesian coordinates of the Chignolin and TC5b mini proteins used in this work.

Atom	X	Y	Z
N	-6.46100	-0.24500	4.44300
C	-6.70200	-0.20000	2.97400
C	-5.36700	-0.27700	2.23300
O	-4.35500	-0.65000	2.79200
H	-7.26800	0.17900	4.94200
H	-5.59500	0.28600	4.66800
H	-6.35000	-1.23300	4.74700
H	-7.32400	-1.03500	2.68600
H	-7.19800	0.72400	2.71800
N	-5.35600	0.07000	0.97500
C	-4.08600	0.01400	0.19600
C	-3.15600	1.14100	0.64700
O	-3.33500	2.28900	0.29300
C	-4.39400	0.17600	-1.29200
C	-3.13100	-0.03400	-2.09400
C	-2.10900	0.92300	-2.05500
C	-2.98300	-1.18500	-2.87800
C	-0.93800	0.72800	-2.80000
C	-1.81300	-1.37800	-3.62300
C	-0.79000	-0.42200	-3.58300
O	0.36200	-0.61500	-4.31700
H	-6.18400	0.36600	0.54200
H	-3.60400	-0.93700	0.36200
H	-5.13300	-0.55300	-1.58600
H	-4.77400	1.17000	-1.47600
H	-2.22300	1.81000	-1.45000
H	-3.77100	-1.92200	-2.90900
H	-0.15000	1.46600	-2.76900
H	-1.69800	-2.26700	-4.22700
H	0.69200	-1.49600	-4.12600
N	-2.15900	0.81800	1.42200
C	-1.21200	1.86700	1.89300
C	-0.13900	2.09500	0.82100
O	0.60800	1.19000	0.50300
C	-0.54200	1.40200	3.18800
C	0.34800	2.52100	3.73200
O	0.12500	3.66200	3.36400
O	1.23700	2.21700	4.50900
H	-2.03200	-0.11400	1.69100
H	-1.75200	2.78200	2.07700
H	-1.30100	1.15600	3.91700
H	0.06200	0.52900	2.98700
N	-0.08800	3.29300	0.29300
C	0.89300	3.65400	-0.74500
C	2.26900	3.88200	-0.11600
O	3.27900	3.89000	-0.79300
C	0.33100	4.94800	-1.33800
C	-0.60500	5.55000	-0.26500
C	-0.99300	4.39600	0.67800
H	0.94200	2.89000	-1.50500
H	1.13800	5.63300	-1.56100
H	-0.23200	4.73300	-2.23400
H	-0.08800	6.32500	0.28500
H	-1.49100	5.95300	-0.73000
H	-0.83000	4.68000	1.70800

H	-2.02100	4.10700	0.52100
N	2.31800	4.05900	1.17500
C	3.63000	4.27600	1.84700
C	4.41600	2.96400	1.83600
O	5.61200	2.94200	2.05000
C	3.39600	4.72200	3.29200
C	4.60500	5.51800	3.78400
C	4.41300	6.99900	3.45200
O	3.88600	7.28500	2.39000
O	4.79700	7.82200	4.26700
H	1.49300	4.04300	1.70400
H	4.18600	5.03600	1.31800
H	2.51200	5.34200	3.33900
H	3.25900	3.85400	3.91900
H	4.70500	5.39700	4.85300
H	5.49800	5.15500	3.29600
N	3.74900	1.87100	1.58400
C	4.44800	0.55600	1.55300
C	4.35700	-0.03100	0.14300
O	5.35300	-0.36900	-0.46500
C	3.78100	-0.39600	2.54900
O	3.70300	0.22900	3.82300
C	4.60300	-1.68200	2.65900
H	2.78500	1.91600	1.41400
H	5.48500	0.69200	1.82300
H	2.78800	-0.63900	2.20600
H	4.46600	0.80400	3.91800
H	4.30800	-2.36600	1.87800
H	4.42800	-2.13800	3.62300
H	5.65200	-1.44800	2.55500
N	3.16800	-0.15200	-0.38300
C	3.01100	-0.71400	-1.75500
C	2.58400	-2.18200	-1.66900
O	3.19800	-3.05100	-2.25600
H	2.37800	0.12800	0.12400
H	2.25900	-0.15100	-2.28900
H	3.95100	-0.64600	-2.28100
N	1.53600	-2.46600	-0.94400
C	1.07200	-3.87900	-0.82700
C	-0.38500	-3.91000	-0.39500
O	-0.88000	-3.00600	0.24800
C	1.92600	-4.62500	0.20100
O	3.18400	-3.98000	0.32900
C	2.13100	-6.06900	-0.25800
H	1.05300	-1.75200	-0.48000
H	1.15400	-4.36600	-1.78500
H	1.42100	-4.62400	1.15600

Table S11: Cartesian coordinates, in Å, of the structure of the Chignolin molecule. The structure corresponds to the 11th model geometry provided in the PDB file of the original reference.

H	3.72900	-4.24400	-0.41600
H	2.96400	-6.50200	0.27500
H	2.33400	-6.08500	-1.31800
H	1.23700	-6.64200	-0.05500
N	-1.08000	-4.94900	-0.75800
C	-2.50400	-5.05500	-0.39200
C	-2.63400	-5.64200	1.01300
O	-2.96100	-4.95200	1.95800
C	-3.20900	-5.96300	-1.39800
C	-4.27100	-5.18200	-2.08800
C	-4.62900	-5.32200	-3.38100
C	-5.11300	-4.13700	-1.53700
N	-5.65200	-4.43000	-3.65900
C	-5.98500	-3.67600	-2.54900
C	-5.20200	-3.55500	-0.26200
C	-6.91800	-2.66800	-2.30400
C	-6.13700	-2.53900	-0.00900
C	-6.99500	-2.09700	-1.02800
H	-0.66100	-5.65700	-1.28200
H	-2.94400	-4.07800	-0.42200
H	-2.49300	-6.32100	-2.12500
H	-3.65200	-6.80000	-0.88200
H	-4.18900	-6.01700	-4.08100
H	-6.09800	-4.33200	-4.52700
H	-4.53800	-3.89100	0.52400
H	-7.57700	-2.33200	-3.09000
H	-6.19800	-2.09700	0.97500
H	-7.71200	-1.31500	-0.82700
N	-2.38000	-6.91000	1.15400
C	-2.48500	-7.54800	2.49700
C	-1.08500	-7.87200	3.02000
O	-0.97300	-8.77100	3.83700
O	-0.14900	-7.21400	2.59600
H	-2.11800	-7.44400	0.37600
H	-2.97900	-6.87000	3.17900
H	-3.05600	-8.46100	2.41800

Atom	X	Y	Z
N	-8.90100	4.12700	-0.55500
C	-8.60800	3.13500	-1.61800
C	-7.11700	2.96400	-1.89700
O	-6.63400	1.84900	-1.75800
C	-9.43700	3.39600	-2.88900
C	-10.91500	3.13000	-2.61100
O	-11.26900	2.70000	-1.52400
N	-11.80600	3.40600	-3.54300
H	-8.33000	3.95700	0.26100
H	-8.74000	5.06800	-0.88900
H	-9.87700	4.04100	-0.29300
H	-8.93000	2.16200	-1.23900
H	-9.31000	4.41700	-3.19300
H	-9.10800	2.71900	-3.67900
H	-11.57200	3.79100	-4.44400
H	-12.75700	3.18300	-3.29400
N	-6.37900	4.03100	-2.22800
C	-4.92300	4.00200	-2.45200
C	-4.13600	3.18700	-1.40400
O	-3.39100	2.27400	-1.76000
C	-4.41100	5.45000	-2.61900
C	-4.79500	6.45000	-1.49500
C	-3.61200	6.80300	-0.59900
C	-5.35100	7.74800	-2.08400
H	-6.82100	4.92300	-2.39400
H	-4.75000	3.49400	-3.40300
H	-3.34000	5.41400	-2.67200
H	-4.81300	5.81700	-3.56400
H	-5.56800	6.02200	-0.85800
H	-3.20700	5.90500	-0.14600
H	-2.84100	7.30400	-1.18300
H	-3.92900	7.47700	0.19700
H	-4.60700	8.20900	-2.73600
H	-6.25500	7.54400	-2.65700
H	-5.59200	8.44500	-1.28100
N	-4.35400	3.45500	-0.11100
C	-3.69000	2.73800	0.98100
C	-4.10200	1.25600	1.07400
O	-3.29100	0.40900	1.44200
C	-3.96400	3.47200	2.30200
C	-2.82400	3.33900	3.29000
C	-2.74600	2.21700	4.13800
C	-1.82000	4.32600	3.33200
C	-1.65700	2.07600	5.01800
C	-0.72500	4.18500	4.20500
C	-0.63900	3.05300	5.04300
O	0.43300	2.88100	5.86100
H	-4.93400	4.24500	0.12000
H	-2.61500	2.76800	0.79600
H	-4.11700	4.51300	2.09100
H	-4.88600	3.09600	2.75000
H	-3.51300	1.45600	4.10100
H	-1.87700	5.20000	2.69500

H	-1.57600	1.22100	5.66900
H	0.03300	4.95200	4.23300
H	1.18700	3.39500	5.56700
N	-5.34200	0.92500	0.68900
C	-5.85700	-0.44900	0.61300
C	-5.08900	-1.22100	-0.47000
O	-4.62100	-2.33400	-0.22600
C	-7.38600	-0.46600	0.34300
C	-8.19700	0.54000	1.19700
C	-7.95900	-1.88400	0.50100
C	-8.01900	0.41200	2.71500
H	-5.90600	1.65600	0.28300
H	-5.67000	-0.94100	1.56800
H	-7.55400	-0.19200	-0.69700
H	-7.90000	1.53100	0.91200
H	-9.25700	0.42400	0.96400
H	-7.50900	-2.55500	-0.23200
H	-7.75900	-2.27100	1.50100
H	-9.03600	-1.87100	0.33200
H	-8.30600	-0.58500	3.04900
H	-6.98300	0.60600	2.99500
H	-8.65600	1.14400	3.21300
N	-4.90700	-0.60100	-1.64500
C	-4.12200	-1.16700	-2.74300
C	-2.62900	-1.32100	-2.39000
O	-1.98600	-2.24000	-2.88400
C	-4.29200	-0.31300	-4.01300
C	-4.24400	-1.17100	-5.29000
C	-5.57600	-1.86000	-5.58500
O	-5.76900	-3.04400	-5.33500
N	-6.53200	-1.14600	-6.15200
H	-5.32700	0.31800	-1.76300
H	-4.51700	-2.16200	-2.94000
H	-5.23800	0.19100	-3.96900
H	-3.49200	0.42900	-4.05300
H	-3.99300	-0.53900	-6.12000
H	-3.45800	-1.92300	-5.20500
H	-6.38900	-0.18400	-6.40800
H	-7.39200	-1.63500	-6.33500
N	-2.07400	-0.45900	-1.52800
C	-0.71600	-0.63100	-0.99300
C	-0.63100	-1.76600	0.04400
O	0.29500	-2.57900	-0.00400
C	-0.22100	0.70300	-0.41700
C	1.14800	0.65200	0.19400
C	2.31900	0.66400	-0.48200
C	1.50800	0.56400	1.60600
N	3.37100	0.56000	0.41100
C	2.92800	0.51500	1.71000
C	0.77900	0.52400	2.81200
C	3.59900	0.44500	2.93800
C	1.43900	0.43300	4.05300
C	2.84200	0.40700	4.12000

H	-2.62400	0.34300	-1.24200
H	-0.05200	-0.90800	-1.81300
H	-0.20600	1.42500	-1.21100
H	-0.92100	1.04400	0.34400
H	2.41200	0.73300	-1.55800
H	4.36000	0.53600	0.15600
H	-0.29900	0.57100	2.77300
H	4.67900	0.41800	2.96100
H	0.86200	0.40000	4.96600
H	3.33400	0.36000	5.08100
N	-1.60000	-1.86000	0.96700
C	-1.64100	-2.93200	1.96300
C	-1.84700	-4.31900	1.34200
O	-1.14400	-5.24800	1.74200
C	-2.71000	-2.64500	3.03300
C	-2.30100	-1.57900	4.06900
C	-3.47500	-1.32300	5.01800
C	-1.09300	-2.00700	4.91400
H	-2.31600	-1.13700	0.99400
H	-0.66600	-2.97800	2.44500
H	-3.60000	-2.30800	2.53700
H	-2.92100	-3.57100	3.57200
H	-2.06100	-0.64900	3.56000
H	-4.34300	-0.99200	4.44900
H	-3.72500	-2.23700	5.56000
H	-3.21100	-0.54900	5.73900
H	-1.27000	-2.98900	5.35400
H	-0.19500	-2.04500	4.30000
H	-0.92200	-1.28600	5.71200
N	-2.75300	-4.48100	0.36000
C	-3.02400	-5.79100	-0.26900
C	-1.79600	-6.42700	-0.93700
O	-1.71900	-7.64800	-1.03000
C	-4.22400	-5.69700	-1.23200
C	-3.93000	-5.00900	-2.57700
C	-3.68200	-5.98600	-3.73600
C	-3.49400	-5.19900	-5.03900
N	-4.56300	-5.48300	-6.02300
H	-3.32100	-3.67500	0.09700
H	-3.30900	-6.47800	0.52900
H	-4.56500	-6.69400	-1.43600
H	-5.01900	-5.14300	-0.73100
H	-4.76900	-4.39000	-2.83000
H	-3.06200	-4.36800	-2.46900
H	-2.79900	-6.56200	-3.53600
H	-4.52400	-6.67400	-3.81800
H	-3.50200	-4.15000	-4.81300
H	-2.51100	-5.43900	-5.45700
H	-4.62100	-6.47400	-6.21100
H	-5.44200	-5.12400	-5.65700
H	-4.38200	-4.98300	-6.88100
N	-0.82800	-5.60700	-1.35500

C	0.46600	-6.01600	-1.90500
C	1.48100	-6.46400	-0.83200
O	2.54500	-6.97100	-1.19400
C	1.03300	-4.83900	-2.72400
C	0.67200	-4.90600	-4.21000
O	-0.53200	-5.05100	-4.52200
O	1.62700	-4.81500	-5.01700
H	-1.01000	-4.61600	-1.29100
H	0.31900	-6.86700	-2.57400
H	0.64400	-3.92400	-2.32000
H	2.11600	-4.83700	-2.65000
N	1.18500	-6.27800	0.46400
C	2.06000	-6.61800	1.59300
C	2.62800	-5.41200	2.35300
O	3.49600	-5.59400	3.20800
H	0.26500	-5.90800	0.69300
H	1.48600	-7.21400	2.30400
H	2.89700	-7.22800	1.25200
N	2.17200	-4.18700	2.05500
C	2.62600	-2.96700	2.72300
C	4.15700	-2.80200	2.65400
O	4.71000	-2.82900	1.55100
H	1.48100	-4.08900	1.31900
H	2.16400	-2.10900	2.23700
H	2.28000	-2.99700	3.75300
N	4.87100	-2.65100	3.79400
C	6.33300	-2.53300	3.80600
C	7.05800	-3.72900	3.16500
O	8.13900	-3.56200	2.60100
C	6.74000	-2.38700	5.27900
C	5.46000	-1.95200	5.98700
C	4.36200	-2.61500	5.16000
H	6.61100	-1.62600	3.26700
H	7.09100	-3.32300	5.67000
H	7.53100	-1.64700	5.40300
H	5.44300	-2.30200	7.00100
H	5.35800	-0.86700	5.92900
H	4.17300	-3.60900	5.51600
H	3.44000	-2.04200	5.24600
N	6.46300	-4.92900	3.20500
C	7.04900	-6.17900	2.70400
C	6.89700	-6.36900	1.18500
O	7.02500	-7.48800	0.69700
C	6.45800	-7.37100	3.47200
O	6.76300	-7.26400	4.85000
H	5.53500	-4.99900	3.61300
H	8.12100	-6.15900	2.90300
H	5.39300	-7.38200	3.34400
H	6.88000	-8.30200	3.09300
H	7.70700	-7.39400	4.97000
N	6.63700	-5.29000	0.43400
C	6.38900	-5.31500	-1.01500

C	7.33200	-4.40500	-1.82300
O	7.08200	-4.12300	-2.99300
C	4.91400	-4.99300	-1.26500
O	4.43100	-5.74300	-2.35800
H	6.50900	-4.41500	0.93000
H	6.56200	-6.32900	-1.37800
H	4.34400	-5.23600	-0.38900
H	4.77800	-3.93400	-1.45700
H	3.71400	-6.32400	-1.98700
N	8.41900	-3.92000	-1.20200
C	9.45100	-3.11600	-1.87000
C	8.98400	-1.72500	-2.31600
O	9.53900	-1.17700	-3.26700
H	8.57300	-4.21000	-0.24600
H	10.29700	-2.98700	-1.19400
H	9.80500	-3.65200	-2.75200
N	7.95600	-1.16400	-1.66000
C	7.28900	0.08400	-2.05400
C	6.85500	0.91600	-0.82900
O	6.22200	0.36600	0.07600
C	6.11000	-0.24300	-2.99400
C	5.04600	-1.17100	-2.37800
C	3.92300	-1.59200	-3.33800
N	4.25100	-2.81100	-4.10000
C	4.85900	-2.91400	-5.27400
N	5.28900	-1.86400	-5.93700
N	5.03500	-4.09500	-5.80900
H	7.57900	-1.67600	-0.87400
H	8.00900	0.66300	-2.63000
H	5.63400	0.67800	-3.26900
H	6.52400	-0.72000	-3.88000
H	5.53800	-2.05900	-2.03100
H	4.57900	-0.65200	-1.54900
H	3.03300	-1.77400	-2.76600
H	3.66900	-0.76500	-4.00300
H	3.96300	-3.69400	-3.69800
H	5.15000	-0.96200	-5.52100
H	5.76100	-1.96200	-6.81500
H	4.64900	-4.89400	-5.32700
H	5.50800	-4.20500	-6.68400
N	7.15600	2.23000	-0.78000
C	6.78200	3.08800	0.34500
C	5.26100	3.33100	0.39500
O	4.58600	3.16500	-0.62400
C	7.55400	4.39400	0.11900
C	7.67700	4.47400	-1.40100
C	7.82000	3.01000	-1.81600
H	7.10700	2.62800	1.27900
H	7.00900	5.23400	0.50500
H	8.54800	4.30800	0.56100
H	6.80000	4.91400	-1.83600
H	8.54000	5.06600	-1.70700

Table S12: Cartesian coordinates, in Å, of the structure of the TC5b molecule. The structure corresponds to the first model geometry provided in the PDB file of the original reference.

H	7.34900	2.84400	-2.76600
H	8.87600	2.73900	-1.85500
N	4.71000	3.73900	1.55500
C	3.28700	4.03100	1.68600
C	2.90100	5.30500	0.91300
O	3.68400	6.25600	0.87100
C	3.03500	4.19000	3.18700
C	4.38500	4.65500	3.72900
C	5.39300	3.94900	2.82300
H	2.71900	3.18100	1.31600
H	2.27400	4.92400	3.37200
H	2.78100	3.22300	3.61800
H	4.48200	5.72100	3.65400
H	4.51800	4.37700	4.77500
H	6.26200	4.56200	2.68200
H	5.66200	2.98300	3.25300
N	1.68800	5.36000	0.33600
C	1.18500	6.54300	-0.35300
C	0.71500	7.60700	0.65500
O	-0.12400	7.32400	1.51300
C	0.04800	6.01400	-1.22900
C	-0.51900	4.85200	-0.41200
C	0.71600	4.27500	0.27200
H	1.96100	6.96600	-0.99100
H	-0.69700	6.77000	-1.38900
H	0.46300	5.63000	-2.16200
H	-1.23200	5.20100	0.31000
H	-1.01900	4.11400	-1.04100
H	0.47000	3.93700	1.26000
H	1.12100	3.46100	-0.32900
N	1.27100	8.82200	0.54900
C	0.85200	10.02700	1.28500
C	-0.40600	10.65700	0.68300
O	-0.38700	10.91600	-0.54000
C	1.97200	11.07100	1.28400
O	3.12000	10.54100	1.91100
O	-1.34100	10.90300	1.47300
H	1.96900	8.96100	-0.16500
H	0.60100	9.76000	2.31000
H	2.21000	11.33800	0.27200
H	1.63600	11.95900	1.82400
H	2.83100	10.04000	2.67600
H	-8.20850	3.50700	-0.97510
H	-3.71620	-5.01100	-6.34000
H	2.50140	-4.70760	-4.69790
H	5.58150	-3.68370	-6.51710
H	-1.26360	10.67050	2.37740

5 Further algorithmic details

This section shows some additional details about the NNAIMGUI code.

5.1 Non-tunable hyper-parameters

As mentioned in the main work, when dealing with partial charges, the NNAIMGUI code employs a collection of fixed hyper-parameters to assign the weight of every atomic correction. These are collected in the GitHub repository of the code.[3]

The following table gathers the electronegativity values employed by the NNAIMGUI code, as given by the Pauling and Sanderson scales, for C, H, O and N atoms. All values were obtained from the literature[4–6].

Table S13: Sanderson (S) and Pauling (P) electronegativity values employed by the NNAIMGUI code for some of the implemented charge equilibration schemes.

χ	C	H	O	N
S	2.746	2.592	3.654	3.194
P	2.550	2.200	3.440	3.040

Similarly, the following table gathers the mean (μ) and the standard deviation (σ) of the error distribution of the NNAIMQ NN models evaluated with the external validation dataset. Additionally, the range of values visited by the predicted atomic charges (r) is also shown.

Table S14: Mean (μ) and standard deviation (σ) of the error distributions for the validation data-sets, along with the range (r) of values visited by the atomic charges. All values are reported in electrons.

	C	H	O	N
μ	$3.77\cdot 10^{-5}$	$-2.98 \cdot 10^{-4}$	$-1.97 \cdot 10^{-4}$	$-2.83 \cdot 10^{-4}$
σ	$1.54 \cdot 10^{-2}$	$8.98 \cdot 10^{-3}$	$1.11 \cdot 10^{-2}$	$2.21 \cdot 10^{-2}$
r	2.60	0.77	0.93	2.58

5.2 Weight distribution schemes

The current section gathers a brief description of the different flavors proposed to assign the atomic weights in the redistribution of the molecular charge.

- 1. Homogeneous weight distribution: the same weight is given to all the atoms.

$$w_A = 1/N. \tag{1}$$

- 2. Charge-based weight distribution: the weight is proportional to the absolute value of the atomic charge (q^{pred}).

$$w_A = \frac{|q_A^{pred}|}{\sum_{B=1,N} |q_B^{pred}|}. \tag{2}$$

- 3. Atomic Population-based weight distribution: the weight is proportional to the atomic population of the atom ($Z - q^{pred}$).

$$w_A = \frac{[Z_A - q_A^{pred}]}{\sum_{B=1,N} [Z_B - q_B^{pred}]}. \tag{3}$$

- 4. Electronegativity (Sanderson)-based weight distribution: the weight is proportional to the Sanderson Electronegativity of the atom (χ^S).

$$w_A = \frac{\chi_A^S}{\sum_{B=1,N} \chi_B^S}. \tag{4}$$

- 5. Electronegativity (Pauling)-based weight distribution: the weight is proportional to the Pauling Electronegativity of the atom (χ^P).

$$w_A = \frac{\chi_A^P}{\sum_{B=1,N} \chi_B^P}. \tag{5}$$

- 6. Standard Deviation (Error)-based weight distribution: the weight is proportional to the standard deviation of the error distribution of the corresponding NNAIMQ model (σ).

$$w_A = \frac{\sigma_A}{\sum_{B=1,N} \sigma_B}. \tag{6}$$

- 7. Mean (Error)-based weight distribution: the weight is proportional to the absolute value of the mean of the error distribution of the corresponding NNAIMQ model (μ).

$$w_A = \frac{|\mu_A|}{\sum_{B=1,N} |\mu_B|}. \tag{7}$$

- 8. The weight is proportional to both the mean (μ) and the standard deviation (σ) of the error distributions of the corresponding NNAIMQ model.

$$w_A = \frac{|\mu_A| \cdot \sigma_A}{\sum_{B=1,N} [|\mu_B| \cdot \sigma_B]}. \tag{8}$$

- 9. The weight is directly proportional to the electron population of the atom ($[Z_A - q_A^{pred}]$) and the mean of the prediction error ($|\mu|$) normalized to the range (r) of values visited by such an element throughout the NNAIMQ dataset.

$$w_A = \frac{\frac{|\mu_A| \cdot [Z_A - q_A^{pred}]}{r_A}}{\sum_{B=1,N} \frac{|\mu_B| \cdot [Z_B - q_B^{pred}]}{r_B}}. \tag{9}$$

- 10. The same strategy as in 9 is applied, but without accounting for the normalization factor.

$$w_A = \frac{|\mu_A| \cdot [Z_A - q_A^{pred}]}{\sum_{B=1,N} |\mu_B| \cdot [Z_B - q_B^{pred}]}. \tag{10}$$

- 11. The same strategy as in 9 is applied, but the weight is also made proportional to the standard deviation of the errors (σ).

$$w_A = \frac{\frac{|\mu_A| \cdot \sigma_A \cdot [Z_A - q_A^{pred}]}{r_A}}{\sum_{B=1,N} \frac{|\mu_B| \cdot \sigma_B \cdot [Z_B - q_B^{pred}]}{r_B}}. \tag{11}$$

Besides the aforementioned schemes, all of them sharing a common functional form, two additional approaches were implemented. The latter, referred to as IRAES and IREES, are based on an iterative strategy:

- 12. Iterative Random Atomic Error Sampling (IRAES): as already mentioned, the error of each atomistic network follows, almost perfectly, a normal distribution. Thus, an equivalent collection of data points (centered at μ and with a width given by σ) can be reproduced through a Gaussian kernel function:

$$f(x, \mu, \sigma) = \frac{1}{\sigma\sqrt{2\pi}} e^{\frac{-(x-\mu)^2}{2\sigma^2}}. \tag{12}$$

  In this way, the random noise inherent to the NNAIMQ values becomes readily available, paving the way towards the iterative correction of the partial charges of the system. In the IRAES method, the correction of each atom is independently drawn form the error distribution and the atomic charges are updated accordingly. This process is repeated iteratively until the ΔQ value becomes lower than a certain threshold (`Qtoler`). It should be noticed that, for the sake of convenience, several scaling factors are employed: `Sigma` (to control the number of points drawn from the immediate vicinity of the center of the Gaussian distribution) and the local electron population N_A (so that larger corrections are applied to atoms with higher electron counts). Additionally, the updating step is proportionally scaled according to the total error, as given by ΔQ, so that more subtle corrections are applied near convergence.

- 13. Iterative Random Elemental Error Sampling (IREES): this method is equivalent to the aforementioned IRAES approach however, the same starting random error bias is used for all the atoms corresponding to a particular chemical element. It is worth pointing out though that, as the actual correction is scaled with local dependent properties, the effective shift made to each atomic charge will still be almost unique for each particle.

It should be noticed that, given the iterative nature of the process, special care should be taken with the updating step near convergence as excessively large values can result in the divergence of the result. Additionally, these methods should only be used when dealing with low ΔQ values, otherwise iteratively redistributing a large excess of molecular charge could heavily increase the noise of the predictions. Something which can even increase the non-physical character of the resultant atomic charges, for instance, the IRAES method could easily break the atomic equivalence exhibited by certain systems as a result of the symmetry of the latter.

Please, do notice that equilibration schemes 6-13 contain model specific values and thus can only be used in combination with the built-in NNAIMQ model.

5.3 Performance metrics

With the aim of measuring the performance of the code, different error and statistical metrics will be used, and in particular:

- MAE (Mean Absolute Error): defined as the average of the difference, in absolute value, between the real and the observed values.

$$MAE = \frac{1}{N} \sum_{i=1,N} |x_i - x_i^*|. \tag{13}$$

- MSE (Mean Squared Error): defined as the average of the square of the difference between the real and the observed vales.

$$MSE = \frac{1}{N} \sum_{i=1,N} (x_i - x_i^*)^2. \tag{14}$$

- Pearson correlation coefficient (R): measuring the extent of the linear dependence between two given variables (i and j).

$$R_{ij} = \frac{S_{ij}}{\sqrt{S_{ii} \cdot S_{jj}}}, \tag{15}$$

  where S_{ij} and S_{ii} represent the covariance and variance, respectively.

5.4 NNAIMQ dataset

To create the original NNAIMQ models[7], a total of 45865 CHON molecules were employed: 42000 structures were used for training whereas the remaining 3865 molecules comprised the external validation dataset, used to test the actual performance of the neural networks (i.e testing). All the structures corresponded to neutral singlet-spin molecules belonging to the near-equilibrium CHON chemical space. With the aim of adequately sampling the latter, most of the compounds were randomly chosen from well-known, robust and validated chemical databases of lead-like compounds such as the GDB13[8], ZINC[9] or ChEMBL[10]. Additional species, including some simple inorganic molecules, such as H_2O or H_2O_2, or CHON based aminoacids were also included for the sake of completeness. Geometry optimizations, molecular dynamics and conformational search studies were performed to provide detailed information about the change of the atomic charges induced by a small perturbation in the corresponding AEVs featurization vectors. Such an approach is particularly convenient as it is crucial to enhance the interpolation abilities of the resultant models. It should be pointed out that the chemical composition and diversity of the testing and training data-sets were shown to be nearly indistinguishable[7] among both data-sets, something which validates the reliability of the train-test-val split employed. Further details about the database employed can be found in the original reference of our work[7].

5.5 Main work-flow

This section gathers some details about the main work-flow to be followed in order to predict QTAIM atomic properties, including equilibrated partial charges, with the NNAIMGUI code. We will focus our attention, primarily, on QTAIM charges, although the same general procedure is followed for any other atomic property.

Upon execution, the code proceeds according to the following work-flow step-by-step.

- 0) The main execution directory, used as the default standard input and output folder for the code, is determined.
- 1) Relevant information is read from the starting input geometry file: name and number of atoms along with the atomic identity and positions.
- 2) The FFNN models (either built-in or external) are loaded and from these the available chemical diversity (the element types that the models have been trained for) is determined.
- 3) If the atomistic information is fine and all the atoms belong to the the current chemical space, the raw values of the atomic property are predicted. This entails the calculation of the chemical featurization descriptors to characterize the local environment of each particle with the internal SFC module, followed by the NN estimation of the individual atomic properties.
- 4) When dealing with atomic charges, the charge equilibration algorithm is applied to the raw predictions, if requested by the user. For such a purpose, the statistical/chemical parameters used in the equilibration schemes are first defined for each atom type and the corresponding equilibration is then applied. For schemes 1-11, the weights are determined as given by the expressions gathered in Section 5.2 and the charges are corrected as:

$$q_A^{pred*} = q_A^{pred} - \frac{w_A \cdot \Delta Q \cdot |q_A^{pred}|}{\sum_{A=1,N} w_A \cdot |q_A^{pred}|}. \tag{16}$$

  It should be noticed that this is also the currently available strategy to be used when employing a custom charge equilibration algorithm. On the other hand, for schemes 12-13, a slightly different approach, based on an iterative kernel, is used. For instance, in scheme 12, a random number is generated for each atom by drawing it from a normal distribution with mean μ and standard deviation σ, corresponding to the main parameters of the error distributions of the NNAIMQ model, as collected in Section 5.1. To bias this distribution, the standard deviation is scaled with the aid of the `Sigma` parameter. In this way, larger values of `Sigma` would result in narrower distributions, forcing the random numbers to be sampled from the immediate vicinity of μ. Since the error distributions are usually symmetrically centered around zero, increasing the value of `Sigma` generally results in smaller corrections at every step, increasing the number of iterations required to achieve convergence. This resultant correction (qcorr) is then scaled with the absolute value of the current molecular charge in such a way that larger corrections are made initially but milder ones are applied nearby convergence. Each atomic charge is then corrected according to the expression:

$$q_A^{pred*} = q_A^{pred} + qcorr \cdot N_A^0 \cdot |\mu|, \tag{17}$$

  where N_A^0 is the uncorrected electron population of the atom, obtained from the raw partial charge ($q_A^{pred,0}$) as $Z_A - q_A^{pred,0}$. After correcting the atomic charge of all the constituting atoms of the system, the resultant molecular charge is computed again and the process is repeated iteratively while the latter is larger than a given threshold, as set by the `Qtoler` variable. This can be represented by the following block of code:

```
while abs(excs_q) > abs(Qtoler):
    for i in np.arange(natoms):
        qcorr= (random.gauss(emean_val[elements[i]],esdev_val[elements[i]]/Sigma))
        qcorr=qcorr*(abs(excs_q))*10
        charges_iter[i]=charges_iter[i]+qcorr*(atomic_number[elements[i]]-charges[i])*
                        (abs(emean_val[elements[i]]))
    excs_q=sum(charges_iter)
```

  On the other hand, scheme 13 uses the same approach, however the correcting factor (qcorr) is set the same for all the atoms belonging to a given element type, as given by:

```
while abs(excs_q) > abs(Qtoler):
    qcorr = {}
    qcorr.clear()
    for key in atomic_number:
        qcorr[key] = (random.gauss(emean_val[key],esdev_val[key]/Sigma))*abs(excs_q)*10
    for i in np.arange(natoms):
        charges_iter[i]=charges_iter[i]+qcorr[elements[i]]*(atomic_number[elements[i]]-charges[i])
                    *(abs(emean_val[elements[i]]))
    excs_q=sum(charges_iter)
```

It is worth mentioning that, owing to the scaling factors included in the algorithm, the actual correction is still specific for each particular atom (e.g the original electron count of the atom is used to scale the correcting offsets, which is likely to be different for multiple atoms belonging to a common element type).

Additionally, it is trivial to realize that there is no guarantee that convergence will be achieved for the iterative procedures as, for instance, a combination of a large molecular charge and a small `Qtoler` value can easily result in a diverging trend. To ameliorate such a problem, the product between the current molecular charge and the initial one is computed. Then, if the resultant value is negative (corresponding to the divergence of the iterative procedure), the loop is broken to prevent a never-ending execution of the code.

- 5) The resultant atomic properties, either equilibrated or not, can be finally printed to the standard output or saved to an output `.nnaim` file.

5.5.1 Command-line execution

As a general rule, the code is usually run throughout command-line executions, which takes, at least, the following execution flags. For the sake of simplicity sval, ival and fval are used to indicate string, integer and float like variables, respectively.

```
python main.py -f filename -gui (yes/no) -fsave (yes/no) -model (NNAIMQ/Custom)
```

- The `-f` (sval) flag is used to specify the name of the file containing the Cartesian coordinates (XYZ) in Å, with an `.xyz` extension.
- The `-gui` (sval) flag controls whether the graphical user interface is launched or not.
- The `-fsave` (sval) option is used to determine whether the results should be saved or not into a standard `.nnaim` file. If set to no, the results will be printed on the standard output (screen).
- The `-model` (sval) flag is used to indicate whether the built-in NNAIMQ or an external model (`Custom`) should be used.

If a `Custom` model is selected, the following flags must be specified:

- The `-model_folder` (sval) is used to set the absolute path the model folder, containing the chemical featurization and the FFNN model files.
- The `-prop` (sval) is used to set the name of the target property. Charge equilibration will only be available if the target property is `QTAIM charges`.
- The `-units` (sval) sets the name of the units of the output target property.

Further details about how to load tailor-made FFNN models are shown in upcoming sections. On the other hand, if charge equilibration is to be applied when dealing with atomic charges, the following execution flags must be specified:

- The `-ceq` (ival), taking as input the integer numbers from -1 to 13, sets the type of charge equilibration scheme to be used. If 0 is chosen, no charge equilibration will be applied to the raw predictions. On the other hand, the -1 option can be used to apply a tailor-made recipe for the equilibration procedure.

- The `-ceq_file` (sval), if a custom charge equilibration is used, this flag is used to set the path to the module containing the atomic weight calculator function.
- The `-sigma` (ival) keyword defines the value used to bias the standard deviation of the error distributions employed in the iterative approaches (`Sigma`). This parameter takes a default value of 1, corresponding to a fully unbiased scenario.
- The `-qtoler` (fval) flag determines the maximum allowed residual molecular charge, in electrons, to be used in the iterative charge equilibration schemes. A default value of 1E-3 electrons is used.

Further details on how to use a tailor-made charge equilibration scheme will be provided in the upcoming sections. Besides these keywords, the `--help` flag can be invoked to print some useful information related to the code and the possible arguments that can be taken during execution.

5.5.2 GUI-interfaced execution

If desired, the estimation and analysis of the atomic properties can be achieved throughout the intuitive Graphical User Interface (GUI) implemented in the code. The aforementioned GUI can be invoked by setting the corresponding gui argument during the command line execution:

```
python main.py -gui yes
```

Or simply by importing the `NNAIMGUI.gui` module within a Python environment:

```
>>> from NNAIMGUI import gui
```

Upon execution, the following window should pop-up on the standard output (screen).

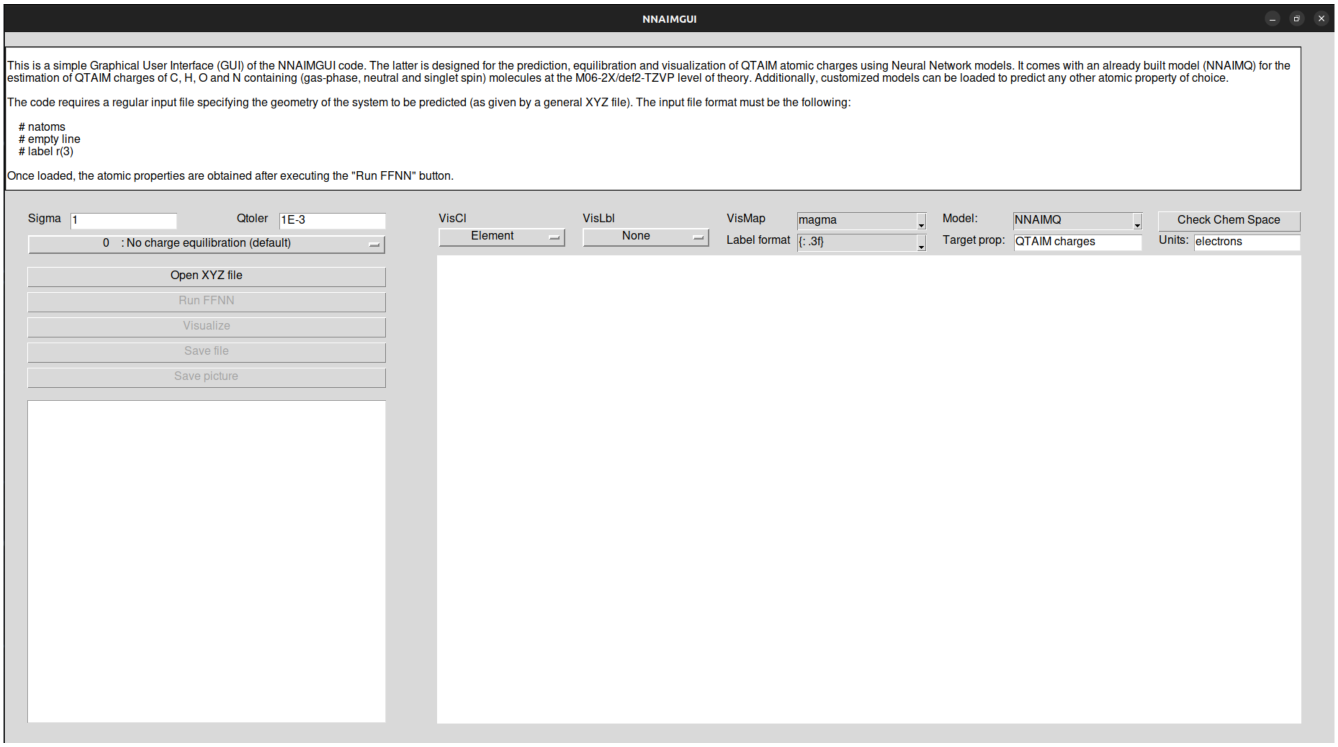

Figure S30: Main dialog of the NNAIMGUI code.

This is the main dialog interface of the NNAIMGUI code with which the atomic properties can be predicted, equilibrated, visualized and saved. Although the code itself is pretty much self-explanatory, a brief overview of the main steps to be followed will be provided. We note in passing that despite the code is written in English, the automatic pop-up dialogues may appear in different languages, as given by the default LANG environment variable set in the computer used to execute the code. Furthermore, it is worth noticing that the GUI has been specifically designed to keep a 16:9 relative aspect ratio, so the actual appearance may change slightly with the configuration of the screen in which the graphical interface is being displayed.

Naturally, the first step is loading the corresponding geometry file (given as the XYZ Cartesian coordinates in Å). Upon clicking the `Open XYZ file` button, the following dialog appears on the screen. The geometry file can be then selected and loaded into the code.

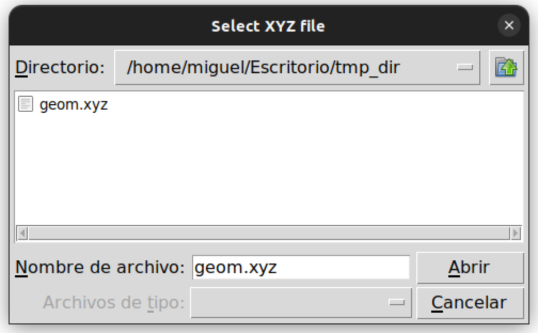

Figure S31: Open file dialog of the NNAIMGUI code.

It should be noticed that the default directory to look for the geometry files will be automatically set to the current execution path. Nevertheless the user is free to surf throughout their folders with the built-in utilities of the dialog box. If the input geometry file is fine the `Run FFNN` button gets enabled. At this point, the model type (`Model`), which will be set to NNAIMQ by default, should be specified by the user. Furthermore, if an equilibration procedure will be applied, the `Sigma` and `Qtoler` parameters along with the `Charge Equilibration Schemes` should be set, otherwise the default values will be used instead. Clicking the `Run FFNN` starts the prediction of the atomic properties, which entails four main steps: (I) loading the model and chemical featurization files (II) computation of the ACSF descriptors, (III) obtaining the raw predictions of the models and (IV) equilibration of the latter values (if requested). Then, the results are shown on the main notification box, appearing on the left side of the main window. The latter can be saved into an output file (`.nnaim`) in the current execution directory with the `Save file` button. Besides the predictions, the output file will contain some additional information about the parameters involved in the current calculation.

Alternatively, the results can be visualized for a more intuitive analysis. This can be done with the `Visualize` button. Upon clicking, a simple three-dimensional representation of the system pops-up in the right panel (plot frame) of the window using the CPK coloring scheme.

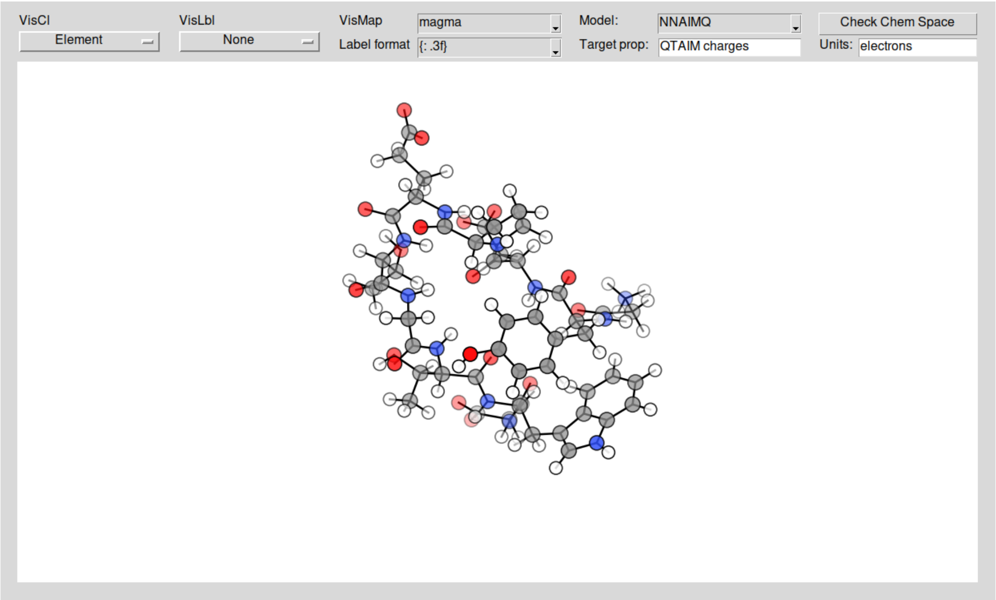

Figure S32: Visualization frame of the NNAIMGUI code showing the molecular framework.

This plot is fully interactive, meaning that rotations and magnifications of the latter can be achieved by clicking and dragging on it as with any other Matplotlib plot.[11] It is also worth noticing that, as a way of representing the depth

of the plot, the color of the particles is slightly faded away based on their distance to the relative origin of the Z-axis. Instead of the default representation code (given by the chemical nature of each atom), the atoms can be colored according to the local values by setting the `VisCl` variable to `Atomic property`, leading to a result similar to that shown in the following figure. Moreover, different color maps can be set with the aid of the `VisMap` drop-down menu.

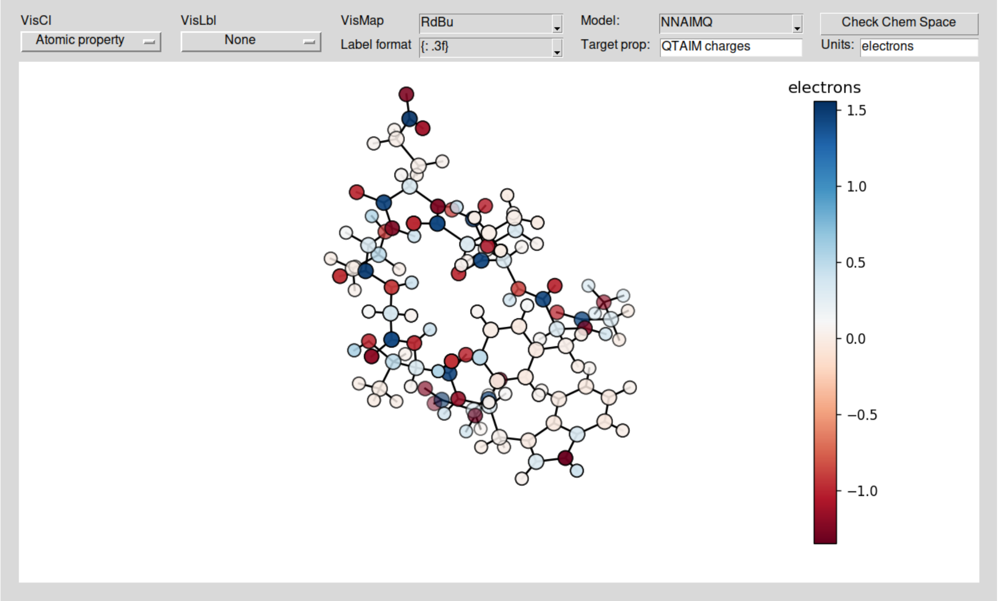

Figure S33: Representation of the estimated atomic properties on top of the main molecular skeleton.

As an additional feature, labels can be included into the molecular representation by selecting one of the options of the `VisLbl` drop-down menu, which can be set either to `Element` or to `Atomic Property`. In the case of the former, the label is comprised by the chemical element of the atom along with its relative numbering in the XYZ file. Whereas for the latter, the actual value of the predicted atomic properties is shown with as many decimal places as that indicated in the `Label format` field. Examples of these two different labeling types are provided in the following figures.

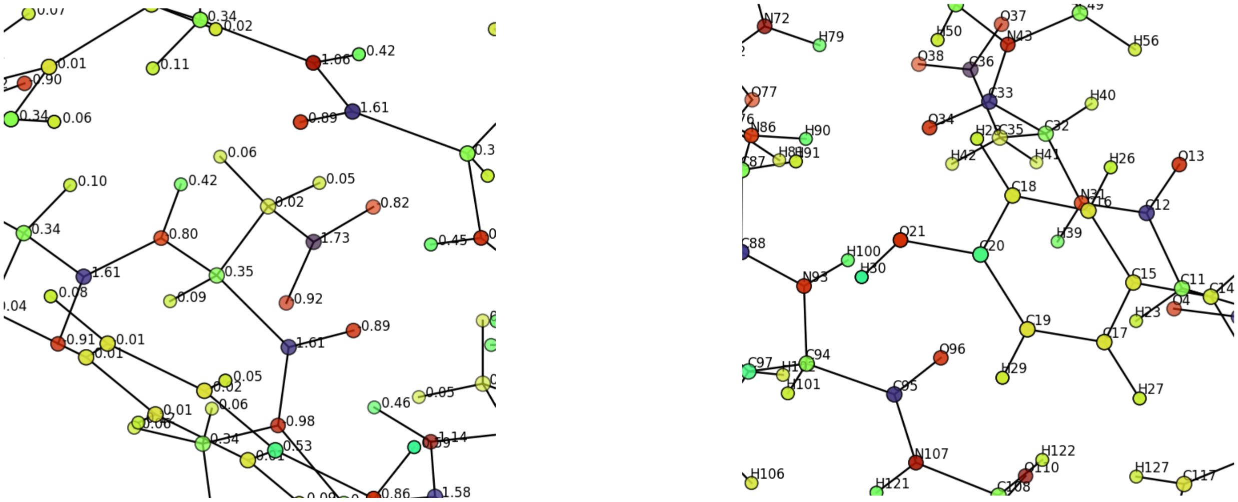

Figure S34: Closeup view of the molecular skeleton showing different labeling: atomic properties (left) and elemental identification (right).

For production purposes, these molecular representations can be rendered and further saved as an image file through the `Save picture` button (it should be noticed that the latter becomes enabled only after the execution of the visualization functionalities of NNAIMGUI). Upon clicking, the code will ask the user to provide an output name for the picture (with a `.png` extension).

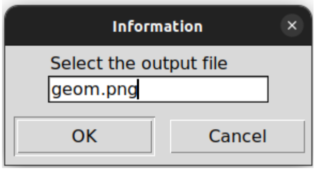

Figure S35: Save output representation dialog of the NNAIMGUI code.

Giving an adequate name for the file will yield the following dialog, where different pieces of information regarding the rendering of the plot can be specified.

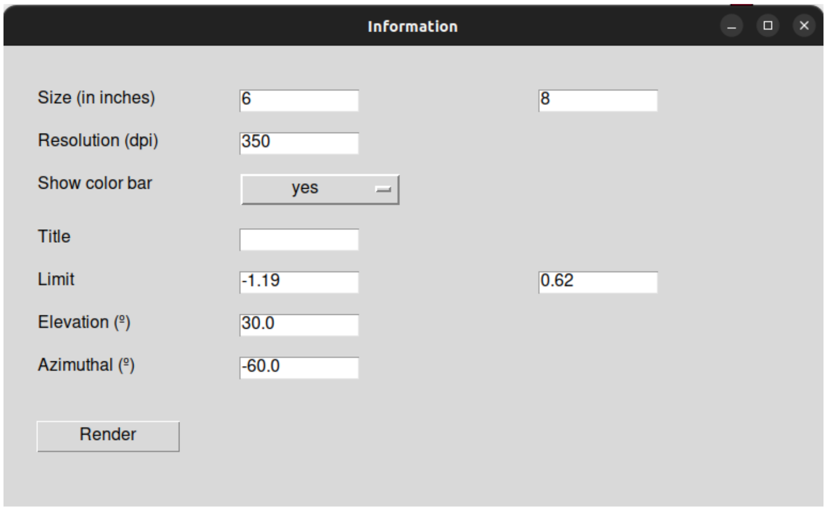

Figure S36: Render plot dialog of the NNAIMGUI code

`Size` is used to specify the size of the resultant figure in inches (Width, Height) and `Resolution` determines the actual resolution of the plot given in dots-per-inch. Additional information such as the title (`Title`) or the inclusion of the color-bar reference scale (`Show color bar`) can be input. Furthermore, the range of values used to define the latter along with the orientation of the plot can be specified with the aid of the `Limit` and `Elevation` or `Azimuthal` variables. It is worth mentioning that, unless otherwise specified, the code uses the values previously set during the live-view visualization of the molecular skeleton. The `Render` button creates and subsequently saves the resultant plot in the previously given output file.

5.6 Using custom models in NNAIMGUI

As mentioned in the main manuscript, our NNAIMGUI code offers the possibility of using custom FFNN models tailor-made for the prediction of any atomic property of choice and within any chemical space.

5.6.1 Loading FFNN models

Either when executed interactively or through the command line, NNAIMGUI will just require the user to provide information about the path to the model folder, name of the target property along with its units. As an example, the following block of code shows how to load a FFNN model trained to predict the QTAIM localization index (can be found in the GitHub repository[3] at /examples/LIAIM).

```
python main.py -f geom.xyz -gui no -fsave no -model Custom -model_folder ./examples/LIAIM -prop
    "Localization index" -units "electrons"
```

On the other hand, if NNAIMGUI is run interactively, after clicking the ”Run FFNN” button the code will ask the user to provide information about the model folder along with the property name and units.

The model folder must contain two different types of files:

- ACSF files: `input.type`, `input.rad` and `input.ang`. These files contain the information required to compute the ACSF features as handled by the internal SFC module.
  - `input.type`: in this file, the id of each element type in the ACSF feature space must be specified. These id numbers will be then used to set the element-specific contributions to the ACSF environment of a given atom. Let's imagine, for instance, a model for water clusters, where the chemical diversity is limited to H and O atoms, then the input.type file must contain the following information:

```
2     # Number of different element types
H     # H will be element type 1
O     # O will be element type 2
```

  - `input.rad`: in this file, the type and parameters of the radial contributions of each neighboring element to the ACSF features of a given atom must be detailed. Two different radial symmetry function kernels are currently implemented in NNAIMGUI:
  [1] Normal Radial Symmetry Function, [12,13] as given by:

$$G_i^{rad} = \sum_{j \neq i}^{N} e^{-\eta(r_{ij}-r_s)^2} \cdot fc(r_{ij}). \tag{18}$$

  [2] Atomic Weighted Radial Symmetry Functions [14], as given by:

$$W_i^{rad} = \sum_{j \neq i}^{N} g(Z_j) \cdot e^{-\eta(r_{ij}-r_s)^2} \cdot fc(r_{ij}), \tag{19}$$

  where $g(Z_j)$ is the atomic factor, depending on the atomic number of each neighboring atom Z_j. In this case, a very simple atomic factor ($g(Z_j) = Z_j$) is employed. On the other hand, fc is a standard cutoff function of the form:

$$f_c(r_{ij}) = \begin{cases} 0.5 \cdot \left[cos(\frac{\pi r_{ij}}{r_c}) + 1\right], r_{ij} \leq r_c \\ 0.0, r_{ij} > r_c, \end{cases} \tag{20}$$

  where r_{ij} is the distance between any two atoms i and j, and r_c defines the cutoff radius.

```
1      # Type of radial sym function (1 normal, 2 Z-weighted)
10.0   # Rcut in Angstroms
5      # Maximum number of radial functions for a given pair
1 1 5  # (atom type, neighboring atom type, number of functions) followed by the Rs and Eta
    values of each function
0.0000000000000000   0.50000000000000000
0.0000000000000000   0.13224489795918368
0.0000000000000000   5.9911242603550297E-002
0.0000000000000000   3.4026465028355393E-002
0.0000000000000000   2.1903731746890212E-002
1 2 5  # O environment for H
0.0000000000000000   0.50000000000000000
0.0000000000000000   0.13224489795918368
0.0000000000000000   5.9911242603550297E-002
0.0000000000000000   3.4026465028355393E-002
0.0000000000000000   2.1903731746890212E-002
2 1 5  # H environment for O
0.0000000000000000   0.50000000000000000
0.0000000000000000   0.13224489795918368
0.0000000000000000   5.9911242603550297E-002
0.0000000000000000   3.4026465028355393E-002
0.0000000000000000   2.1903731746890212E-002
2 2 5  # O environment for O
0.0000000000000000   0.50000000000000000
0.0000000000000000   0.13224489795918368
```

```
0.0000000000000000   5.9911242603550297E-002
0.0000000000000000   3.4026465028355393E-002
0.0000000000000000   2.1903731746890212E-002
```

The file will contain as many radial function blocks as possible atomic pairs, in our case 4: (1,1), (1,2) (2,1), (2,2). Notice that (1,2) and (2,1) are not equivalent as the former refers to the radial contribution of neighboring O atoms to the ACSF features of H atoms whereas the latter is the contribution of neighboring H atoms to a given O atom in the molecule.

– `input.ang`: in this file, the type and parameters of the angular contributions of each neighboring pair to the ACSF features of a given atom must be specified. Currently, NNAIMGUI accounts for a total of 4 different angular symmetry function kernels:

[1] Normal Angular Symmetry function,[12,13] as given by:

$$G_i^{ang} = 2^{1-\xi} \sum_{j\neq i}^{N} \sum_{k\neq i,j}^{N} [(1+\lambda \cdot cos\theta_{ijk})^{\xi} \cdot e^{-\eta[(r_{ij}-r_s)^2+(r_{ik}-r_s)^2+(r_{jk}-r_s)^2]} \cdot f_c(r_{ij}) \cdot f_c(r_{ik}) \cdot f_c(r_{jk})]. \quad (21)$$

[2] Modified Angular Symmetry function,[13] as given by:

$$G_i^{ang} = 2^{1-\xi} \sum_{j\neq i}^{N} \sum_{k\neq i,j}^{N} [(1+\lambda \cdot cos\theta_{ijk})^{\xi} \cdot e^{-\eta[(r_{ij}-r_s)^2+(r_{ik}-r_s)^2]} \cdot f_c(r_{ij}) \cdot f_c(r_{ik})]. \quad (22)$$

[3] Heavily Modified Angular Symmetry Function,[15] as given by:

$$G_i^{ang} = 2^{1-\xi} \sum_{j,k\neq i}^{N} (1+cos(\theta_{ijk}-\theta_s))^{\xi} \cdot exp\left[-\eta\left(\frac{r_{ij}+r_{ik}}{2}-r_s\right)^2\right] \cdot f_c(r_{ij}) \cdot f_c(r_{ik}). \quad (23)$$

[4] Atomic Weighted Angular Symmetry Functions,[14] as given by:

$$W_i^{ang} = 2^{1-\xi} \cdot h(Z_j,Z_k) \cdot \sum_{j\neq i}^{N} \sum_{k\neq i,j}^{N} [(1+\lambda \cdot cos\theta_{ijk})^{\xi} \cdot e^{-\eta[(r_{ij}-r_s)^2+(r_{ik}-r_s)^2+(r_{jk}-r_s)^2]} \cdot f_c(r_{ij}) \cdot f_c(r_{ik}) \cdot f_c(r_{jk})], \quad (24)$$

where $h(Z_j, Z_k)$ represents the so-called atomic pair factor, taking the following functional form:

$$h(Z_j, Z_k) = Z_j \cdot Z_k, \quad (25)$$

with Z_j and Z_k being the atomic numbers of the neighboring atoms j and k constituting the atomic triplet centered at an atom i. In all cases, the previously shown cutoff function is used.

```
3      # Angular sym function type (1 normal, 2 modified, 3 heavily modified, 4 Z-weighted)
10.0   # Rcut in Angstroms
5      # Maximum number of angular functions for a given pair
1 1 5 # (atom, neighboring pair, number of functions) followed by the Rs, Xi, Eta, Lambda/Theta
    values
0.0000000000000000     1.0000000000000000     1.0000000000000002E-002 0.0000000000000000
0.0000000000000000     1.0000000000000000     1.9036541768875849E-002 0.0000000000000000
0.0000000000000000     1.0000000000000000     3.6238984729102551E-002 0.0000000000000000
0.0000000000000000     1.0000000000000000     6.8986482119689760E-002 0.0000000000000000
0.0000000000000000     1.0000000000000000     0.13132640382981661     0.0000000000000000
1 2 5 # HO/OH environment of H atoms
0.0000000000000000     1.0000000000000000     1.0000000000000002E-002 0.0000000000000000
0.0000000000000000     1.0000000000000000     1.9036541768875849E-002 0.0000000000000000
0.0000000000000000     1.0000000000000000     3.6238984729102551E-002 0.0000000000000000
0.0000000000000000     1.0000000000000000     6.8986482119689760E-002 0.0000000000000000
0.0000000000000000     1.0000000000000000     0.13132640382981661     0.0000000000000000
1 4 5 # OO environment of H atoms
0.0000000000000000     1.0000000000000000     1.0000000000000002E-002 0.0000000000000000
```

```
0.0000000000000000      1.0000000000000000      1.9036541768875849E-002 0.0000000000000000
0.0000000000000000      1.0000000000000000      3.6238984729102551E-002 0.0000000000000000
0.0000000000000000      1.0000000000000000      6.8986482119689760E-002 0.0000000000000000
0.0000000000000000      1.0000000000000000      0.13132640382981661     0.0000000000000000
2 1 5 # HH environment of O atoms
0.0000000000000000      1.0000000000000000      1.0000000000000002E-002 0.0000000000000000
0.0000000000000000      1.0000000000000000      1.9036541768875849E-002 0.0000000000000000
0.0000000000000000      1.0000000000000000      3.6238984729102551E-002 0.0000000000000000
0.0000000000000000      1.0000000000000000      6.8986482119689760E-002 0.0000000000000000
0.0000000000000000      1.0000000000000000      0.13132640382981661     0.0000000000000000
2 2 5 # HO/OH environment of O atoms
0.0000000000000000      1.0000000000000000      1.0000000000000002E-002 0.0000000000000000
0.0000000000000000      1.0000000000000000      1.9036541768875849E-002 0.0000000000000000
0.0000000000000000      1.0000000000000000      3.6238984729102551E-002 0.0000000000000000
0.0000000000000000      1.0000000000000000      6.8986482119689760E-002 0.0000000000000000
0.0000000000000000      1.0000000000000000      0.13132640382981661     0.0000000000000000
2 4 5 # OO environment of O atoms
0.0000000000000000      1.0000000000000000      1.0000000000000002E-002 0.0000000000000000
0.0000000000000000      1.0000000000000000      1.9036541768875849E-002 0.0000000000000000
0.0000000000000000      1.0000000000000000      3.6238984729102551E-002 0.0000000000000000
0.0000000000000000      1.0000000000000000      6.8986482119689760E-002 0.0000000000000000
0.0000000000000000      1.0000000000000000      0.13132640382981661     0.0000000000000000
```

It should be noticed that now, the second id number in the heading of each block does not refer to an atom type but to an atomic pair type. These ids are obtained following a very simple recipe:

```
ind  = np.zeros(telem, dtype=int)
for i in range(1,telem+1):
    ind[i-1] = (i-1)*(2*telem-i)
for i in range(telem):
    for j in range(i, telem):
        pair_id= ind[i] + j + 1
```

The user can quickly check the corresponding id numbers with the aid of the following built-in function, which receives as input the number of element types:

```
>>> from NNAIMGUI import SFC
>>> SFC.show_neighmat(2)
Element i 1 and Element j 1, id : 1
Element i 1 and Element j 2, id : 2
Element i 2 and Element j 2, id : 4
```

The input.ang will contain then as many angular function blocks as possible atomic trios, formed from the combination of a given atom type and all the possible different neighboring atomic pair. Notice that now, the heteroatomic neighboring pairs are equivalent owing to the kernel used to compute the ACSF functions (e.g H+O or O+H neighboring pairs have the same id (2)).

- model files: `nn(name)X.h5`, `nn(name)X.std`, `nn(name)X.mean`, `nn(name)X.min` and `nn(name)X.max`. Where `X` is the chemical symbol of the element for which each atomistic model has been trained for and name is any name of your choice. The `.h5` file is the actual Tensorflow model in an h5 format. The `.std`, `.mean`, `.min` and `.max` are plain text files containing the standard deviation, mean, minimum and maximum value taken by each ACSF feature throughput the training, which will be used to homogeneously standardize the data. The model folder must contain as many sets of model files (`.h5, .std, .mean, .min, .max`) as element specific atomistic models are present. For further details, check the example given in the /src/examples/LIAM/ path.

The available chemical diversity will be determined based on the elements (`X`) appearing in each sets of model files, so the use of standard chemical symbols is mandatory.

5.6.2 Loading tailor-made charge equilibration schemes

If desired, the user is free to use a tailor-made charge equilibration, different from those provided by default with the NNAIMGUI code. The latter will require the user to specify the path to the corresponding Python file (with `.py` extension) which must containing a function (`weight_calc`) which receives as input the charges and the chemical symbols (stored in the elements list) and returns the weights (`w`). As an example, the following block of code shows a charge equilibration scheme where the correction is made proportional to the Van der Waals radii of the atoms:

```
import numpy as np
from NNAIMGUI.dictionaries import *

def weight_calc(charges,elements):
   w = []
   w.clear()
   natoms=int(len(elements))
   size=[]
   for i in np.arange(natoms):
      size.append(radii[elements[i]])
   tot_size=sum(size)
   for i in np.arange(natoms):
      w.append(size[i]/tot_size)
   w=np.asarray(w,dtype=float)
   return w
```

After computing the weights, the charges are corrected as shown in Eq. 16.

5.7 Training models in NNAIMGUI

As an additional feature, NNAIMGUI implements a built-in module (`trainer`) for building and training new atomistic models. In this way the module allows non-experienced users to train FFNN models in a simple and effective way, which can be later used to run predictions on NNAIMGUI. The first step is transforming the standard XYZ coordinates into a ML database comprising the target property and the ACSF features of each atom. This can be done with the aid of the `xyz2dtbase` built-in function:

```
from NNAIMGUI import trainer
database=trainer.xyz2dtbase(datafile="test.xyz",itype="input.type",rtype="input.rad",atype="input.ang",
        fsave='yes')
```

Where `datafile` is the database in extended XYZ format and `itype`, `rtype` and `atype` are the input.type, input.rad and input.ang files gathering the main parameters of the chemical featurization. If the `fsave` option is set to "yes", element-specific database files will be saved in the local directory. The extended XYZ file employs the following format:

```
natom                # number of atoms of geom 1
label x y z prop     # Chemical symbol, R(:) coordinates, atomic property of a given atom
. . . .
. . . .
natom                # number of atoms of geom 2
label x y z prop
. . . .
. . . .
```

Once the database has been created, the models can be trained from the previously stored files (if saved), as:

```
trainer.train_from_csv(datafile="test.xyz_H.dtbse",ftra=0.8,vsplit=0.2,nepochs=100000,patnc=25,lr=0.000001,
      loss='mse',optimizer='RMSprop',neurons=(10,10,10),activations=('tanh','tanh','linear'))
```

Taking as parameters:

- `ftra` : fraction of data used for training
- `vsplit` : validation split factor
- `nepochs` : maximum number of EPOCHS allowed for training
- `patnc` : patience of the early-stopping approach
- `lr` : learning rate
- `loss` : loss metric used to track the progress of the training
- `optimizer` : model optimizer
- `neurons` : list containing the number of neurons of the hidden layers
- `activations`: list containing the activation functions of the model

Or alternatively, by parsing the database directly to the `train` function while specifying the element for which the model will be built:

```
trainer.train(name="test",database=database,elem="C",ftra=0.8,vsplit=0.2,nepochs=100000,patnc=25,
        lr=0.000001,loss='mse',optimizer='RMSprop',neurons=(10,10,10),
        activations=('tanh','tanh','linear'))
```

In this way, all possible atomistic models for the chemical diversity of the database can be easily built in a few lines of code:

```
from NNAIMGUI import trainer
database=trainer.xyz2dtbase(datafile="test.xyz",itype="input.type",rtype="input.rad",atype="input.ang",
            fsave='yes')
for elem in set([item[0] for item in database]):
    trainer.train(database=database,elem=elem,ftra=0.8,vsplit=0.2,nepochs=100,patnc=25,
            lr=0.000001,loss='mse',optimizer='RMSprop',neurons=(10,10,10),
            activations=('tanh','tanh','linear'))
```

For the sake of simplicity, the current NNAIMGUI trainer module uses deeply connected FFNN models.

References

[1] Frisch, M. J. *et al.* "Gaussian 09 Revision E.01", Gaussian Inc. Wallingford CT 2009.

[2] Martín Pendás, A.; Francisco, E. "Promolden. A QTAIM/IQA code", .

[3] Gallegos, M. "NNAIMGUI, https://github.com/m-gallegos/NNAIMGUI", 2023.

[4] Allred, A. "Electronegativity values from thermochemical data", *J. Inorg. Nucl. Chem.* **1961,** *17,* 215-221.

[5] Huheey, J. E.; Keiter, E. A.; Keiter, R. L. *Inorganic chemistry;* HarperCollins College: 4 ed.; 1993.

[6] Sanderson, R. T. "Principles of electronegativity Part I. General nature", *J. Chem. Educ.* **1988,** *65,* 112.

[7] Gallegos, M.; Guevara-Vela, J. M.; Pendás, A. M. "NNAIMQ: A neural network model for predicting QTAIM charges", *J. Chem. Phys.* **2022,** *156,* 014112.

[8] Blum, L. C.; Reymond, J.-L. "970 Million Druglike Small Molecules for Virtual Screening in the Chemical Universe Database GDB-13", *J. Am. Chem. Soc.* **2009,** *131,* 8732-8733.

[9] Irwin, J. J.; Shoichet, B. K. "ZINC: A Free Database of Commercially Available Compounds for Virtual Screening", *J. Chem. Inf. Model.* **2005,** *45,* 177-182.

[10] Gaulton, A.; Bellis, L. J.; Bento, A. P.; Chambers, J.; Davies, M.; Hersey, A.; Light, Y.; McGlinchey, S.; Michalovich, D.; Al-Lazikani, B.; Overington, J. P. "ChEMBL: a large-scale bioactivity database for drug discovery", *Nucleic Acids Res.* **2011,** *40,* 1100–1107.

[11] Hunter, J. D. "Matplotlib: A 2D graphics environment", *Comput. Sci. Eng.* **2007,** *9,* 90–95.

[12] Behler, J.; Parrinello, M. "Generalized Neural-Network Representation of High-Dimensional Potential-Energy Surfaces", *Phys. Rev. Lett.* **2007,** *98,*.

[13] Behler, J. "Atom-centered symmetry functions for constructing high-dimensional neural network potentials", *J. Chem. Phys.* **2011,** *134,* 074106.

[14] Gastegger, M.; Schwiedrzik, L.; Bittermann, M.; Berzsenyi, F.; Marquetand, P. "wACSF—Weighted atom-centered symmetry functions as descriptors in machine learning potentials", *J. Chem. Phys.* **2018,** *148,* 241709.

[15] Smith, J. S.; Isayev, O.; Roitberg, A. E. "ANI-1: an extensible neural network potential with DFT accuracy at force field computational cost", *Chem. Sci.* **2017,** *8,* 3192–3203.
